# Characterization of pharmacogenomic variants in a Brazilian admixed cohort of elderly individuals based on whole-genome sequencing data

**DOI:** 10.3389/fphar.2023.1178715

**Published:** 2023-05-10

**Authors:** Luciana Bertholim-Nasciben, Marilia O. Scliar, Guilherme Debortoli, Bhooma Thiruvahindrapuram, Stephen W. Scherer, Yeda A. O. Duarte, Mayana Zatz, Guilherme Suarez-Kurtz, Esteban J. Parra, Michel S. Naslavsky

**Affiliations:** ^1^ School of Public Health, University of São Paulo, São Paulo, Brazil; ^2^ Human Genome and Stem Cell Research Center, University of São Paulo, São Paulo, Brazil; ^3^ Department of Anthropology, University of Toronto at Mississauga, Mississauga, ON, Canada; ^4^ The Centre for Applied Genomics, The Hospital for Sick Children, Toronto, ON, Canada; ^5^ Department of Molecular Genetics, Faculty of Medicine, University of Toronto, Toronto, ON, Canada; ^6^ Medical-Surgical Nursing Department, School of Nursing, University of São Paulo, São Paulo, Brazil; ^7^ Department of Genetics and Evolutionary Biology, Biosciences Institute, University of São Paulo, São Paulo, Brazil; ^8^ Divisão de Pesquisa Clínica e Desenvolvimento Tecnológico, Instituto Nacional de Câncer, Rio de Janeiro, Brazil; ^9^ Hospital Israelita Albert Einstein, São Paulo, Brazil

**Keywords:** pharmacogenomics, admixture, population cohort, whole-genome sequencing, PharmGKB, CPIC guidelines

## Abstract

**Introduction:** Research in the field of pharmacogenomics (PGx) aims to identify genetic variants that modulate response to drugs, through alterations in their pharmacokinetics (PK) or pharmacodynamics (PD). The distribution of PGx variants differs considerably among populations, and whole-genome sequencing (WGS) plays a major role as a comprehensive approach to detect both common and rare variants. This study evaluated the frequency of PGx markers in the context of the Brazilian population, using data from a population-based admixed cohort from Sao Paulo, Brazil, which includes variants from WGS of 1,171 unrelated, elderly individuals.

**Methods:** The Stargazer tool was used to call star alleles and structural variants (SVs) from 38 pharmacogenes. Clinically relevant variants were investigated, and the predicted drug response phenotype was analyzed in combination with the medication record to assess individuals potentially at high-risk of gene-drug interaction.

**Results:** In total, 352 unique star alleles or haplotypes were observed, of which 255 and 199 had a frequency < 0.05 and < 0.01, respectively. For star alleles with frequency > 5% (*n* = 97), decreased, loss-of-function and unknown function accounted for 13.4%, 8.2% and 27.8% of alleles or haplotypes, respectively. Structural variants (SVs) were identified in 35 genes for at least one individual, and occurred with frequencies >5% for CYP2D6, CYP2A6, GSTM1, and UGT2B17. Overall 98.0% of the individuals carried at least one high risk genotype-predicted phenotype in pharmacogenes with PharmGKB level of evidence 1A for drug interaction. The Electronic Health Record (EHR) Priority Result Notation and the cohort medication registry were combined to assess high-risk gene-drug interactions. In general, 42.0% of the cohort used at least one PharmGKB evidence level 1A drug, and 18.9% of individuals who used PharmGKB evidence level 1A drugs had a genotype-predicted phenotype of high-risk gene-drug interaction.

**Conclusion:** This study described the applicability of next-generation sequencing (NGS) techniques for translating PGx variants into clinically relevant phenotypes on a large scale in the Brazilian population and explores the feasibility of systematic adoption of PGx testing in Brazil.

## Introduction

The concept of precision medicine is based on identifying individuals at risk of developing diseases, their trajectories, and their individual response to treatments. Pharmacogenomics (PGx) plays an important role in precision medicine and deals with interindividual variation of drug response due to genetic variants across the genome, which affect the efficacy and toxicity of drugs through alterations in their pharmacokinetics (PK)—absorption, bioavailability, distribution, metabolism, and excretion—or pharmacodynamics (PD) (Relling and Evans, 2015).

The Pharmacogenomics Knowledgebase (PharmGKB^1^) is a major partner in PGx research and implementation in clinical practice, through the collection of primary PGx data, curation, and annotation of peer-reviewed literature on gene–drug associations (Barbarino et al., 2018; Whirl-Carrillo et al., 2021). Presently, there are more than 800 drugs in the PharmGKB database, for which associations with genetic variants have been reported^2^. However, only a limited fraction of these associations, comprising 148 drugs, has been translated into genotype-based dosing recommendations by PGx-focused independent initiatives such as the Clinical Pharmacogenetics Implementation Consortium (CPIC) (Relling et al., 2020).

Several factors play a role in the translation of PGx findings into the clinic and objective criteria such as “levels of evidence” from PharmGKB which are considered by the CPIC as the main standard to classify these findings. The level of evidence of each gene–drug relationship is determined by the parameters of the study, such as strength of association, effect size, cohort size, and the reproducibility of the results^3^. It is important to note that genomic diversity and variation play a major role, since the distribution of PGx variants (both occurrence of rare variants and frequency of common polymorphisms) and the extent of linkage disequilibrium (LD) differ considerably among populations, with important implications for design of clinical trials and genome-wide association studies (GWASs) (Suarez-Kurtz and Parra, 2018).

For Brazilians and other Latin American (LA) populations, distinct patterns of admixture between different continental groups create additional challenges to PGx implementation in clinical practice (Suarez-Kurtz and Pena, 2007; Suarez-Kurtz and Parra, 2018). Accordingly, standardized guidelines to inform and adjust prescriptions based on PGx data require deeper knowledge of the frequency and effect size of PGx variants across Latin American populations. In this context, whole-genome sequencing (WGS) plays a major role as a comprehensive approach to detect both common and rare variants, and in the development of algorithms to predict the functionality of rare variants (Suarez-Kurtz and Parra, 2018).

This study assessed PGx markers in the Brazilian population, using data from the “Health, Well-Being, and Aging Study” (SABE—*Saúde, Bem-estar e Envelhecimento*), a population-based cohort from Sao Paulo, Brazil, which includes variants from whole-genome sequences of 1,171 unrelated individuals. The frequency of star alleles of pharmacogenes was investigated, and the predicted phenotypes were analyzed in combination with medication records for the assessment of individuals potentially at high-risk for gene–drug interactions.

## Materials and methods

### SABE project and the study population

The SABE study cohorts comprise population-based probability samples of individuals aged 60 years and older and were designed to provide information on health indicators of the elderly population in the city of São Paulo, Brazil, through comprehensive in-home interviews and biological sample collection every 5 years. This cross-sectional study included individuals who participated in the third wave of data collection in 2010. A detailed description of the project under the coordination of the School of Public Health at the University of São Paulo (FSP-USP) can be found elsewhere (Lebrão and Laurenti, 2005; Naslavsky et al., 2022). Individuals responded to a long questionnaire which included questions related to self-reported health conditions, such as hypertension, heart and cardiovascular conditions, diabetes, cancer, chronic pulmonary disorders, joint conditions, osteoporosis, anemia, and depression. One section of the questionnaire is devoted to collect information about medication and supplement intake. Biological samples were collected, including peripheral blood for DNA biobanking.

The SABE project was approved by the FSP-USP Institutional Review Board and the National Committee of Ethics in Research. All participants signed consent forms according to the Brazilian regulatory requirements human research.

### Variant discovery

A total of 1,335 SABE participants were enrolled in the 2010 round of data collection. DNA was extracted from these individuals, and 1,200 DNA samples met the quality criteria and were submitted to WGS. Further details on the adopted methodology can be found in the WGS flagship publication (Naslavsky et al., 2022). Briefly, Illumina HiSeqX sequencers were used with a 30X target coverage and a 150 base paired-end single index read format. Relatedness was assessed by the KING toolset (Manichaikul et al., 2010), and to avoid inflating population frequencies for rare alleles, only one individual (proband) was maintained when identifying siblings, duos, or other pairs of up to three degrees of relationships. GATK flags (Auwera et al., 2013) and an in-house genotype and variant flagging algorithm were applied to filter out low-quality variants, as described in Naslavsky et al. (2022). The variants and allelic frequencies of the aggregated sample of 1,171 unrelated individuals submitted to WGS are publicly available on the ABraOM (*Arquivo Brasileiro Online de Mutações*) platform^4^. The frequency of clinically important variants in *CACNAS1S*, *CFTR*, *DPYD*, *IFNL3*, and *RYR1* were extracted from the ABraOM dataset since these genes are not represented in the Stargazer pipeline.

### Pharmacogenes star allele assessment

WGS data had been previously mapped to human reference GRCh38 using ISIS analysis software (Raczy et al., 2013), and reads overlapping with the 38 pharmacogenes of interest regions were extracted from BAM files, converted to FastQ files, and realigned to hg19 using BWA-MEM v0.7.12 (Li and Durbin, 2009). Duplicate reads were removed using MarkDuplicates from Picard v1.79. GATK3.7 (Auwera et al., 2013) was used for indel realignment, base quality score recalibration, and variant joint calling. The following hard filters were applied to exclude low-quality SNPs (QD < 2.0, FS > 60.0, MQ < 40.0, MQRankSum < −12.5, ReadPosRankSum < −8.0, and SOR >3.0) and indels (QD < 2.0, FS > 200.0, ReadPosRankSum < -20.0, and SOR >10.0). Read-depth files were obtained through the GATK’s DepthOfCoverage function with mapping and base quality thresholds of 20 or greater.

The star alleles of 38 pharmacogenes were called using the tool “genotype” from Stargazer v1.0.7 genotyping pipeline (Lee et al., 2019a; Lee et al., 2019b), which uses the hard-filtered vcf and coverage files as input, the program Beagle (Browning et al., 2018), and the 1000 Genomes Project (Auton et al., 2015) haplotype as a reference panel for phasing. Phased SNVs and indels were then matched to star alleles. Stargazer used the read depth from the coverage file to convert to copy number by performing intrasample normalization using read depth from a control GDF file (Lee et al., 2019a). Diplotypes are defined as two alleles or haplotypes carried by a given individual.

### Predicted phenotype assignment

For pharmacogenes with PharmGKB evidence level 1A, PharmGKB’s diplotype–phenotype translation tables^5^ were used to map each individual diplotype to a predicted phenotype and calculate phenotype frequencies (Supplementary Figure S1). Diplotypes that were not listed in translation tables were assigned as unknown functions, with the exception of *CYP2D6*, for which several diplotypes present in our sample were missing in PharmGKB’s translation tables, and the sum of the activity score (AS) for each allele was used to assign the predicted metabolic phenotype according to the CPIC guidelines (Caudle et al., 2020).

Furthermore, predicted phenotypes were matched to the Electronic Health Record (EHR) Priority Result Notation (risk)^5^, and the frequency of individuals at high-risk of gene–drug interaction was verified.

Since diplotype–phenotype translation tables have not been reported for *CYP4F2* and *VKORC1*, the assignment of predicted phenotypes was interpreted from the Warfarin dosing guideline (Johnson et al., 2017). CYP4F2*3 (c.1297G>A; p. Val433Met; rs2108622) is listed as a decreased function allele and individuals with one or two copies of CYP4F2*3 were assigned as higher warfarin dose phenotype. *VKORC1* (c.-1639G>A, rs9923231) is associated with warfarin sensitivity, and patients with one or two –1639A require progressively lower warfarin doses. Thus, individuals with one or two copies of VKORC1*2 (rs9923231, and the linked rs9934438 and rs235961) were assigned as decreased warfarin dose phenotype.

### Medication analysis

The SABE in-home interview includes the collection of information about the medication that respondents were taking at the time of the interview. The medication section from the 2010 round of data collection was first checked against a list of 45 PharmGKB evidence level 1A drug–gene pairs (Supplementary Figure S1) to obtain the fraction of the cohort who has taken one or more of the drugs listed. Then, potential PGx interactions were verified by checking the individuals that had any predicted phenotype designated as high-risk following EHR Priority Result Notation and had also taken any specific drug associated with the high-risk phenotype.

## Results

### Samples

A total of 1,171 unrelated, elderly Brazilian individuals from the SABE 2010 cohort passed sample-level QC criteria, including 427 (36.5%) men and 744 (63.5%) women, with a median age of 71 years (IQR = 64–80). The distribution of self-reported color/race categories, according to the Brazilian census was “White,” 58.1% (*n* = 680); “Brown” (*Pardo* in Brazilian Portuguese), 28.2% (*n* = 330); “Black,” 6.4% (*n* = 75); “Yellow” (referring to Asian extraction), 2.7% (*n* = 32); others, 2.1% (*n* = 25); and no answer, 2.5% (*n* = 29). It is important to note that these census “color/race” classifications are sociopolitical and not biological constructs. In Brazil, multiple studies have highlighted the complex relationships between “color/race” and genetic ancestry (e.g., estimates of the geographical origin of the ancestors of the individuals in a sample based on statistical inferences made from genomic data) (Pena et al., 2011; Naslavsky et al., 2022), and we provide a relevant example when discussing the distribution of *CYP2C9* variants in one of the sections of this article. We would like to note that when writing self-reported “White or Black” persons, we are referring to the Brazilian census categories.

### Star alleles assessment

**FIGURE 1:**
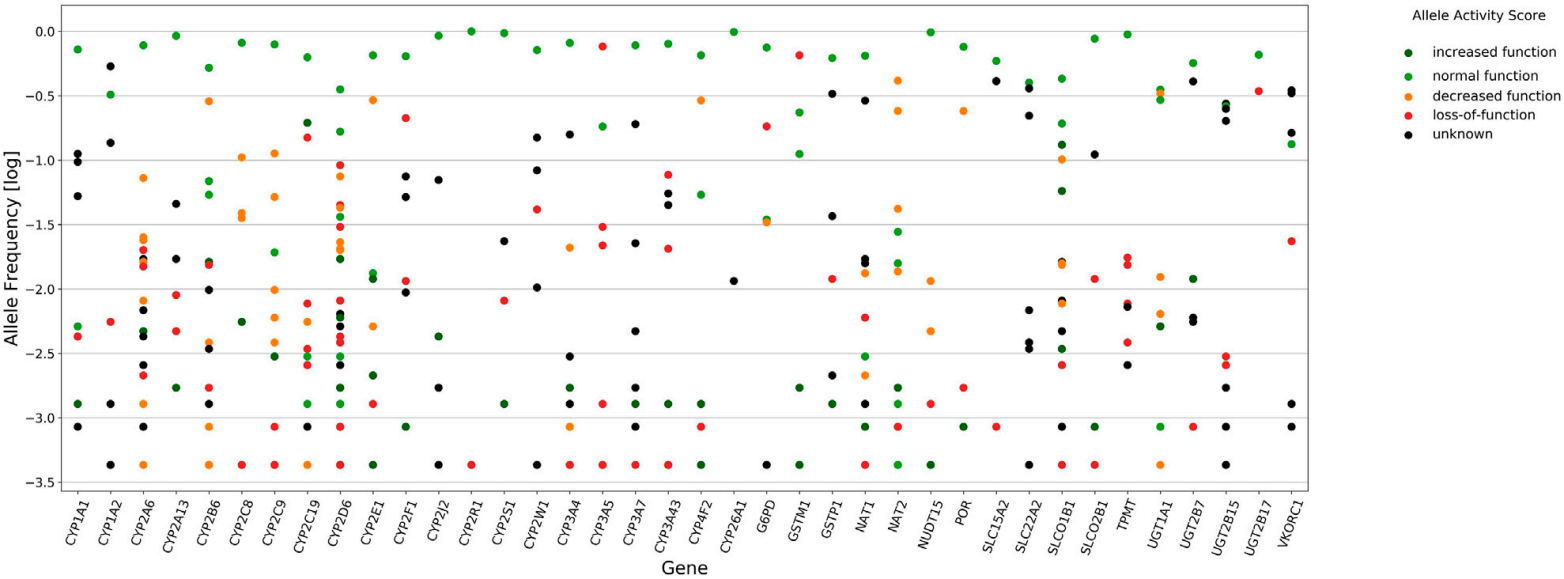
Star alleles frequency of 38 pharmacogenes called by Stargazer 1.0.7. Circles in vertical line denote frequency distribution of star alleles for each gene. Mark colors represent allele functions as attributed by Stargazer, dark green (increased function, AS of 1.5–3.0), light green (normal function, AS of 1.0), orange (decreased function, AS of 0.5), red (loss-of-function, AS of zero), and black (unknown function).

Stargazer 1.0.7 was used to call star alleles in 38 pharmacogenes in the study cohort, and the frequency of haplotypes and diplotypes are presented in Supplementary Tables S1, 2, respectively. In total, 352 unique star alleles or haplotypes were observed in all pharmacogenes assessed. Among these, 255 and 199 had a frequency <5% and <1%, respectively. For star alleles with frequency >5% (*n* = 97), decreased function, loss-of-function, and unknown function accounted for 13.4%, 8.2%, and 27.8% of alleles or haplotypes, respectively (Figure 1). Structural variants (SVs) were identified in 35 genes for at least one individual, although the allele frequencies of SVs were >5% only for *CYP2D6*, *CYP2A6*, *GSTM1*, and *UGT2B17*. A total of 103 different duplications/deletions of star alleles and 16 different rearrangements in *CYP2A6*, *CYP2B6*, *CYP2D6*, *CYP2E1*, *SLC22A2*, and *UGT2B15* were identified. The allele frequency of complete deletion of *GSTM1* and *UGT2B17* was 65.2% and 34.3%, respectively (Supplementary Table S1).

*CYP2D6* showed the highest levels of polymorphism among all genes, with 43 identified unique star alleles (Figure 1), and 23.9% of individuals carrying SVs including rearrangements with loss-of-function (*68+*4, *4N+*4, *13), decreased function (*36+*10) and normal function (*S1+*1 and *83+*2), and copy number variation (CNV) ranging from zero to three gene copies. Among alleles that have not been reported in PharmVar, we detected *CYP2D6* *21 × 2 and *83+*2 and two new star alleles referred to as *S1+*1 and *S2+*1 (Supplementary Figure S2).

A total of 21 *CYP* genes were analyzed, and the highest frequency of loss-of-function and decreased function alleles, considering the AS of 0 or 0.5 designated by Stargazer, occurred in *CYP3A5* (81.7%), followed by *CYP2D6* (38.6%), *CYP2B6* (30.9%), *CYP2E1* (29.8%), *CYP4F2* (29.2%), *CYP2F1* (22.3%), *CYP2C9* (18.6%), *CYP2A6*, and *CYP2C8* (18.5%). The frequency of loss-of-function and decreased function alleles was less than 10% in *CYP2W1*, *CYP3A4*, *CYP2A13*, *CYP2S1*, *CYP1A2*, *CYP1A1*, *CYP2J2*, *CYP3A7*, *CYP2R1*, and *CYP3A43*.

The frequency of *NAT2* decreased function star alleles was 71.0%. In the case of *NAT1*, normal function and unknown function alleles accounted for 64.6% and 32.5%, respectively. POR*28 (decreased function) had a frequency of 24.0%. The combined frequency of decreased function NUDT15*3 and NUDT15*4 was 1.6%.

Four solute carrier (SLC) transporter genes were studied (*SLCO1B1*, *SLCO2B1*, *SLC15A2*, and *SLC22A2*), and *SLCO1B1* showed the highest degree of polymorphism with 20 identified star alleles, 15.5% alleles with loss-of-function or decreased function, and 19.3% of alleles with increased function.

The combined frequency of *TPMT* loss-of-function alleles (*2, *3A, *3B, and *3C) was 4.4%. Four UDP-glucuronosyltransferase (UGT) phase II metabolism enzymes were evaluated. UGT1A1*28 is the most common decreased function allele and had a frequency of 32.9%. The decreased function allele *UGT1A1* *37 had a frequency of 1.2%, while UGT1A1*6 and UGT1A1*7 were detected at a frequency <0.1%.

Most of the star alleles from *VKORC1* were designated by Stargazer as “unknown function” (84.4%), including VKORC1*2 defined by the variant rs9923231 (and the linked rs9934438 and rs235961), related to warfarin dosage, which has a frequency of 33.1%.

### Variant analysis of *CACNAS1S*, *CFTR*, *DPYD*, *IFNL3*, and *RYR1*

In addition to the evaluation of star alleles, the SABE cohort WGS dataset (deposited in ABraOM) was used to verify the frequency of clinically important variants in *CACNAS1S*, *CFTR*, *DPYD*, *IFNL3*, and *RYR1* (Supplementary Table S3). No *CACNA1S* or *RYR1* actionable variants were found, and three individuals were identified with the rare loss-of-function *DPYD* variant rs3918290 in heterozygosis.

### Clinically actionable pharmacogenes

**FIGURE 2:**
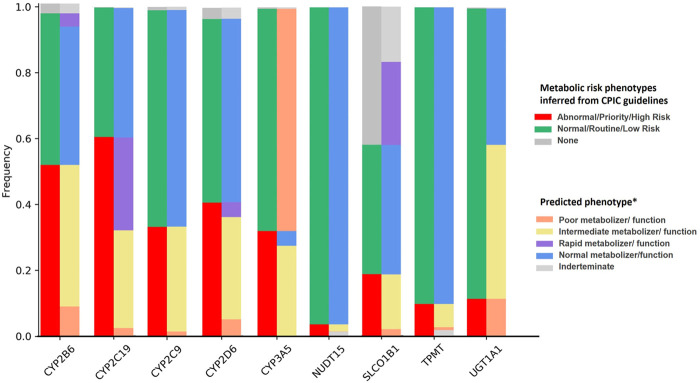
Frequency of predicted metabolizer/function phenotypes and gene–drug priority risk categories based on the CPIC guidelines for pharmacogenes with PharmGKB level of evidence 1A for drug interaction. Bars on the left represent phenotype risk using Electronic Health Record Priority Result Notation. Bars on the right indicate the predicted phenotype obtained by matching diplotypes assigned by Stargazer to phenotypes using PharmGKB/CPIC diplotype–phenotype reference tables [Poor/Intermediate/Normal/Rapid] function refers to SLCO1B1 phenotypes. *Predicted phenotype categories were grouped as follows: Intermediate metabolizer/function also includes: CYP2C19 Likely Intermediate Metabolizer (CYP3A5/NUDT15/TPMT) Possible Intermediate Metabolizer, SLCO1B1 Decreased function, and SLCO1B1 Possible Decreased Function; poor metabolizer/function also includes: CYP2C19 Likely Poor Metabolizer, and SLCO1B1 Possible Poor Function; and rapid metabolizer/function also includes SLCO1B1 Possible Increased Function, and CYP2D6 Ultrarapid Metabolizer.

To estimate the possible clinical impact of the findings, the predicted phenotype was obtained by analyzing the genotype data of 1,171 individuals for pharmacogenes with PharmGKB evidence level 1A. For this, PharmGKB reference translation tables^5^ were used to map each individual diplotype to a predicted phenotype and then to individual EHR priority result notation (Supplementary Figure S1), which is used to predict individuals at potential risk of an adverse or untoward response to medications due to the gene–drug interaction (Figure 2). Overall, 98.0% of the individuals carried at least one high-risk genotype in nine genes analyzed.

A total of 49 diplotypes were identified in *CYP2B6*, half of which were associated with intermediate or poor metabolizer phenotypes and classified as high risk for an adverse or poor response to medications that are metabolized by *CYP2B6* (51.6% of the individuals). The frequency of diplotypes with one or two copies of CYP2B6*6, associated with lower protein expression and activity, was 45.8%.

In the case of *CYP2C19*, carriers of one or two copies of *17, *3, and *2 are considered at potential risk of an adverse or poor response to medications metabolized by the gene. Due to the high frequency of those diplotypes in the cohort, mainly *CYP2C19* *1/*17 (24.3%), *1/*2 (19.6%), and *2/*17 (6.4%), *CYP2C19* had the highest frequency of genotypes associated with actionable phenotypes.

According to the CPIC, individuals with intermediate and poor metabolizer CYP2C9 phenotypes are considered to be at high risk for adverse reactions to medications that are affected by CYP2C9. The intermediate metabolizer diplotypes *CYP2C9* *1/*2 and *1/*3 were present in 17.5% and 8.4% of individuals, respectively, and a total of 33.2% were classified into the high-risk EHR annotation group.

The frequency of individuals at high risk for an adverse or untoward response to medication metabolized by CYP2D6 was 40.8%, including ultrarapid (4.6%), intermediate (31.3%), and poor metabolizer phenotypes (5.0%). The most frequent high-risk diplotypes were *CYP2D6* *1/*4 (6.15%), *1/*68+*4 (3.59%), *1/*5 (2.56%), and *2/*4 (2.56%), all intermediate metabolizers.

CYP3A5 high-risk phenotypes, which are normal and intermediate metabolizers, were verified in 4.5% and 27.4% of our cohort, respectively, and consist of diplotypes of one or two copies of *CYP3A5* *1.

Less than 20% of the individuals were found to be at high risk of gene–drug interactions due to variants in *NUDT15*, *SLCO1B1*, *TPMT*, and *UGT1A1*. For *NUDT15*, intermediate metabolizer (*1/*3) is considered high-risk phenotypes and occurred in 2.1% of individuals. The frequency of individuals with *SLCO1B1* high-risk phenotypes (decreased function or poor function) was 18.7%, including individuals with one or two copies of *SLCO1B1* *5, *15, or *17. *TPMT* intermediate metabolizer, poor metabolizer, and indeterminate metabolizer phenotypes are considered to be at high risk of gene–drug interactions and consisted of 9.8% of individuals. The frequency of individuals with *UGT1A1* *28/*28 (poor metabolizer predicted phenotype) was 11.3%, which is the only high-risk *UGT1A1* diplotype identified in our cohort.

**FIGURE 3:**
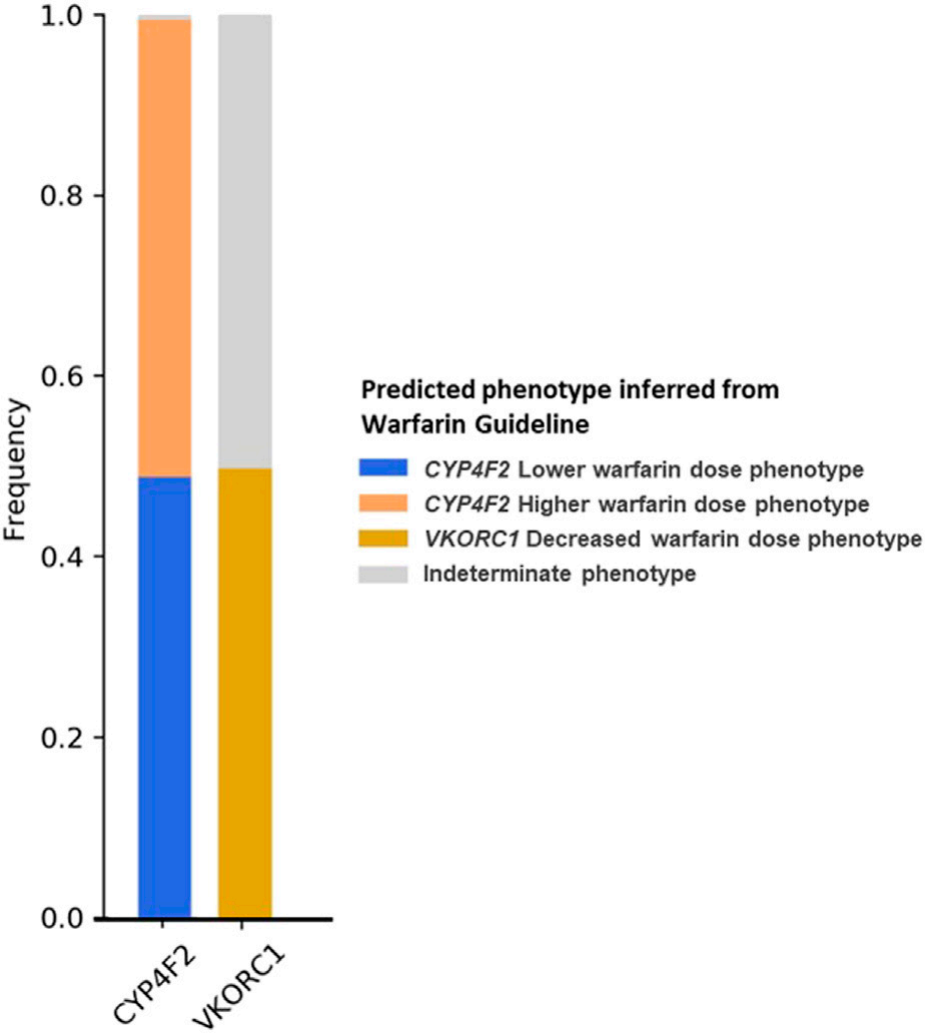
Frequency of predicted metabolizer/function phenotypes for *CYP4F2* and *VKORC1* based on the warfarin dosing guideline (Johnson et al., 2017).

*CYP4F2* and *VKORC1* had the EHR Priority Result Notation designated according to warfarin guidelines. Therefore, the *CYP4F2* lower warfarin dose phenotype individuals (carriers of one or two copies of CYP4F2*3) were 48.8% of our cohort, and *VKORC1* decreased warfarin dose phenotype (carriers of one or two copies of VKORC1*2), which accounted for 49.8% of our cohort (Figure 3).

### Real-world medication use

The analysis of medication use was performed to predict the clinical impact of the findings in a real-world scenario. For this, the PharmGKB evidence level 1A medication used in outpatient settings was verified in the SABE questionnaire. The median number of drugs used was four (IQR = 2–6). In general, 42.0% of the individuals used at least one PharmGKB level 1A drug, 18.9% were at potentially high risk for adverse effects, and 2.7% had more than one PGx interaction. Simvastatin and omeprazole were in use by 20.1 and 19.5% of the cohort, amytriptiline by 3.3%, sertraline by 2.3%, and ibuprofen by 2.2%. All other drugs were taken by <1.5% of the cohort (Table 1 and Supplementary Table S4).

**TABLE 1:** Cohort medication use and predicted high-risk individuals by pharmacogene.

Gene	Drugs^a^	% Taking drugs (n)	% Taking drugs at high risk (n)^b^
***CYP2C19***	Amitriptyline, citalopram, clomipramine, clopidogrel, escitalopram, imipramine, lansoprazole, omeprazole, pantoprazole, and sertraline	25.1 (278)	14.5 (161)
***SLCO1B1***	Simvastatin	20.1 (223)	3.0 (33)
***CYP2D6***	Amitriptyline, clomipramine, codeine, fluvoxamine, imipramine, metoprolol, nortriptyline, paroxetine, propafenone, risperidone, tramadol, and venlafaxine	6.7 (74)	2.4 (27)
***CYP2C9***	Celecoxib, ibuprofen, meloxicam, phenytoin, piroxicam, tenoxicam, and warfarin	4.7 (52)	1.5 (17)
***CYP4F2/VKORC1***	Warfarin	1.4 (15)	0.5 (5)
***CYP2B6***	Efavirenz	0.2 (2)	0.1 (1)

^a^ Drugs identified in the cohort among 45 PharmGKB evidence level 1A drug–gene pairs (Supplementary Figure S1).

^b^ Individuals whose predicted phenotype was designated as high-risk following the EHR Priority Result Notation and has also taken any specific drug associated with the high-risk phenotype.

## Discussion

Genetic variation plays an important role on drug response, and the scientific community has been making efforts in the last few decades to translate PGx advances into the clinic. A better understanding of human genomic diversity is necessary to broaden the scope of clinical guidelines and implementation of PGx-informed prescription and also has important implications for the design of clinical trials. The present study uncovers the frequency of star alleles and predicted phenotypes of 38 pharmacogenes from WGS data for a population-based sample containing more than 1,000 individuals of admixed ancestry from Brazil (Naslavsky et al., 2022), with a distinctive demographic history as compared to other Latin American populations (Popejoy and Fullerton, 2016; Sirugo et al., 2019). Available medication data were used to assess potential pharmacogenes–drug interactions.

In general, the frequencies of the star alleles identified in the SABE WGS cohort were in accordance with previous studies in the Brazilian population (Rodrigues-Soares et al., 2018; Rodrigues-Soares et al., 2020), although a higher number of star alleles with a frequency <5% (*n* = 255) and <1% (*n* = 199) have been identified in our study. Genotyping technologies are able to identify the most frequent variants but lack the ability to reveal rare and deleterious ones that have been proven to play a significant role in the field of PGx (Ingelman-Sundberg et al., 2018; De Mattia et al., 2022; Gray et al., 2022). An analysis of the UK Biobank sample, which included nearly 50,000 subjects with both imputed data from genotyping arrays and exome sequencing, compared their ability to call haplotypes and phenotypes in 14 clinically important pharmacogenes. Despite the high concordance between techniques for most genes, the analysis revealed extremely low concordance for highly polymorphic pharmacogenes such as *CYP2D6*, where imputed data from genotyping arrays may not capture the wide range of variation (McInnes et al., 2021).

Several initiatives have used NGS to analyze PGx markers in diverse populations, using large genomic databases (Reisberg et al., 2019; Luo et al., 2021; McInnes et al., 2021; Taliun et al., 2021) or databases from specific populations (Cohn et al., 2017; Al-Mahayri et al., 2020; Mauleekoonphairoj et al., 2020). The advances in genomic analyses by NGS have enabled scientists to investigate the contribution of rare variants to complex diseases and PGx. Recently, Ingelman-Sundberg et al. (2018) analyzed the distribution of rare and common variants in 208 pharmacogenes by analyzing the exome sequencing data from 60,706 unrelated individuals from ExAC. They reported that the vast majority of variants were rare (98.5%; MAF <1%) or very rare (96.2%; MAF <0.1%), and for those variants, there was a strong enrichment in consequences predicted to cause functional alterations, suggesting that a substantial part of the unexplained interindividual differences in drug metabolism phenotypes can be attributed to rare genetic variants.

NGS-based techniques have also facilitated the large-scale detection of SVs (i.e., microscopic or submicroscopic genomic alterations comprising DNA segments larger than 50 bp), including CNVs (duplications or deletions of DNA segments), translocations, inversions, and combinations of the same (Feuk et al., 2006; Trost et al., 2018). Numerous algorithms have been developed for detecting SVs from large-scale sequencing data, and they differ in both sensitivity and specificity mainly due to differences in SV-related and sequencing library-related properties, leading to variable depth of coverage (Guan and Sung, 2016). This heterogeneity motivated the development of best practices for the detection of germline CNVs, which represent the majority and the most clinically significant type of SVs, from short-read WGS data (Trost et al., 2018), less prone to variability in sequencing depth of coverage.

SVs have been shown to play a clear role in the field of PGx (Johansson and Ingelman-Sundberg, 2009; He et al., 2011; Santos et al., 2018) and were originally described in *CYP2D6*, which metabolizes around 25% of all drugs in clinical use, and has been extensively studied to uncover the functionality of its multiple variants and haplotypes including CNVs and other complex SVs that are not detected by conventional techniques. Its homology to the pseudogenes, *CYP2D7* and *CYP2D8*, becomes a challenge for the interrogation by short-read NGS (Schwarz et al., 2019; Luo et al., 2021). Recently, specific bioinformatics algorithms have been developed for pharmacogenes genotyping based on high-throughput sequencing data, most of them using *CYP2D6* as a model, such as Stargazer (Lee et al., 2019a; Lee et al., 2019b), Astrolabe (formerly Constellation) (Twist et al., 2016), Aldy (Numanagi et al., 2018), and StellarPGx (Twesigomwe et al., 2021). In our analysis with the Stargazer pipeline, *CYP2D6* showed a high degree of polymorphism with a long tail of low-frequency alleles; 33 out of 43 star alleles were identified with a frequency <1%, and 19 out of 33 rare alleles were classified as abnormal function alleles (loss-of-function, decreased function, or increased function alleles, Supplementary Table S1). Several low-frequency alleles have not been reported in the Brazilian population (Rodrigues-Soares et al., 2018), including the loss-of-function rearrangement *CYP2D6* *68+*4 (4.5%) and decreased function rearrangement CYP2D6*36+*10 (frequency <1%). The frequency of the CYP2D6*5 gene deletion was comparable to the frequency described by Rodrigues-Soares et al. (2020) in 98 Brazilians genotyped for *CYP2D6* polymorphisms. The frequency of *CYP2D6* functional allele multiplication (extensive metabolizers) was 2.5%, corroborating the pattern identified in LLerena et al. (2014) in Latin American populations. SVs were also identified in other 34 of 38 genes analyzed by Stargazer, although they are rare for most of the genes, corroborating Santos et al. (2018), who described novel exonic deletions and duplications in 201 of 208 pharmacogenes analyzed (97%) in the ExAC dataset. Testing the accuracy of SVs identified in PGx-specific pipelines as compared to the best practices available for WGS-based SV and CNV pipeline (Trost et al., 2018) will be followed up in the future.

We verified that 98.0% of the individuals carried at least one high-risk genotype in the nine genes analyzed, and 24.8% of the individuals were predicted to be at a high risk for PGx interactions for both CYP2C19 and CYP2D6, for example*.* In terms of analysis of real-world medication data, 42.0% of the individuals used at least one drug with PGx recommendation (PharmGKB level 1A). An important aspect is that our cohort is made up of census-sampled individuals aged 60 years and older with a high rate of polypharmacy, considering that 41.1% of the individuals reported taking five or more medications regularly.

A recent systematic review (O’Shea et al., 2022) indicated that, mainly in a multi-drug scenario, panel-based tests have provided optimistic estimates of long-term cost savings, especially due to a reduction in the number of emergency department (ED) visits and the number of rehospitalizations in patients submitting to PGx tests (Brixner et al., 2016; Elliott et al., 2017). Although an economic analysis is beyond the scope of this study, a previous study conducted in a public hospital in Brazil showed that 14.6% of ED visits were associated with drug-related morbidity, with an estimated annual cost of approximately USD 7.5 million (Freitas et al., 2017).

More research involving economic evaluations of PGx implementation in Brazil is needed and should also include a discussion of the genotyping methodology. Although the idea of using NGS-based techniques in the clinics has been challenged due to the current costs and the complexity involved in the interpretation of results (O’Shea et al., 2022), as we have discussed previously, several advances in terms of techniques have been made in recent years and targeted genotyping tends to continuously be replaced by NGS-based approaches, including WGS (Caspar et al., 2021).

In our real-world in-home medication usage evaluation, *CYP2C19*, *SLCO1B1*, *CYP2D6*, *CYP2C9*, and *CYP4F2* e *VKORC1* were the most important genes from a PGx perspective. *CYP2C19* contributes to the metabolism of a wide range of drugs, including the platelet aggregation inhibitor clopidogrel, proton pump inhibitors, antidepressants, carisoprodol, and diazepam, in addition to endogenous substances, such as melatonin and progesterone (Aquilante et al., 2013; Botton et al., 2021). In our study, *CYP2C19* was the gene with the highest frequency of predicted phenotypes at a potential high risk for an adverse or poor response to medications metabolized by the gene, in agreement with studies in other populations (Biswas, 2021; Luo et al., 2021). Although medications used in the hospital setting were not considered in this analysis, we could envisage the potential clinical impact of *CYP2C19* in terms of real-world medication usage, as drugs that potentially interact with CYP2C19 had the highest frequency of use among all the gene–drugs pairs analyzed. In total, 25.1% of the cohorts were taking medications with potential *CYP2C19* gene–drug interactions, and 14.5% of the cohorts were high-risk individuals who have actually taken one or more of the drugs at home. The frequency of use of omeprazole was 19.5%, while other drugs were 8.8%, including amitriptyline, sertraline, citalopram, and clopidogrel.

The frequency of individuals at high-risk for *SLCO1B1* gene–drug interaction was 18.7% (with one or two copies of SLCO1B1*5, *15, or *17), and the real-world in-home medication usage analysis showed that simvastatin was the medication taken with the highest frequency (20.1%) and 3% of the cohort individuals were taking simvastatin with a high risk of adverse drug reactions. The frequency of individuals reporting statin-related skeletal muscle toxicity, such as myalgias, myopathy, and rhabdomyolysis, is considered low (1–5%), but the high frequency of prescription results in an important absolute number of events (Ramsey et al., 2014).

CYP2C9 is involved in the oxidative metabolism of up to 15–20% of all drugs undergoing phase I metabolisms, such as warfarin, non-steroidal anti-inflammatories, and phenytoin (Lee et al., 2002; Van Booven et al., 2010). In our analysis, ibuprofen, warfarin, and phenytoin were the most commonly used drugs metabolized by CYP2C9. The frequency of decreased function CYP2C9*2 and *3 varies among populations, and in the Brazilian population, both alleles showed significant differences between self-reported “White” and “Black” individuals (Rodrigues-Soares et al., 2018). CYP2C9*5, *6, *8, and *11 are decreased function alleles that are found with the highest frequency in individuals of African ancestry^6^. Based on the significant difference in allele frequencies in populations of diverse ancestry, the warfarin guidelines have distinct dosing algorithms in patients who self-identify as African, which includes CYP2C9*5, *6, *8, *11, *vs.* non-African. In the SABE sample, the allele frequencies of CYP2C9*2 and *3 were 11.3% and 5.2%, respectively, and the allele frequencies of CYP2C9*5, *6, *8, and *11 were <1%. However, the percentage of individuals with at least one copy of CYP2C9*5, *6, *8, or *11 was 3.92%, and 19.0% of these were self-identified as “White,” 59.5% as “Brown,” and 19.0% as “Black” individuals, corroborating previous studies that demonstrated a tenuous correlation between self-reported “race” and biogeographical ancestry (Pena et al., 2011; Suarez-Kurtz and Parra, 2018).

In addition to *CYP2C9*, warfarin dosing algorithms include VKORC1*2 (-1639G>A) as a dose-reduction factor, due to the increased sensitivity to warfarin associated with this variant (Johnson et al., 2017). The frequency of individuals with both *CYP2C9* intermediate/poor metabolism phenotypes and *VKORC1* alleles with increased warfarin sensitivity was 14.4% in our cohort. For these individuals, lower doses or an alternative oral anticoagulant might be considered (Johnson et al., 2017). Warfarin dosing algorithms also include the detection of CYP4F2*3 as optional and only for individuals of non-African ancestry. The carriers of one or two copies of CYP4F2*3 would require an increase in warfarin dose of 5–10% (Johnson et al., 2017).

The current study has some limitations. First, the initial cohort was composed of 1,343 individuals, but 143 did not have WGS performed because either they did not provide biological samples or the DNA quality did not reach WGS standards. In addition, 29 pairs of individuals had family relationships up to the third degree, which required exclusion of one of the individuals of the pair to avoid rare variant inflation in the population dataset (Naslavsky et al., 2022). Second, the collection of data regarding medication usage was taken in 2010, and we might not have access to all medications taken by individuals, including drugs taken in the hospital setting. The medication intake questionnaire aimed to collect information on drug usage at the moment of interview, and no dosage annotation was obtained. In addition, Stargazer v1.0.7 has a tool for predicting phenotypes that has not yet been systematically validated and was not used in this study. Other pipelines for pharmacogenes genotyping based on NGS data could be used in the future to validate the results of this same set of data.

## Conclusion

Although microarrays have been shown to be a cost-effective tool for the identification of pharmacogenetic variants and dosing adjustments (Reisberg et al., 2019), genome sequencing technologies continue to improve in terms of read length, data analysis, and variant interpretation. Our study used the Stargazer pipeline (Lee et al., 2019a) to call star alleles from a WGS database of 1,171 individuals from the city of Sao Paulo, Brazil. This study illustrates the feasibility of using such techniques on a large scale in the Brazilian population, with the advantage of unraveling complex pharmacogene structures, such as CYP2D6, rare variants, and other CNVs, that have been proved to play an important role in the field of pharmacogenetics and drug response (Santos et al., 2018). The investigation showed that 98.0% of the individuals carried at least one high-risk genotype in 9 PharmGKB evidence level 1A pharmacogenes, and a significant proportion of individuals were at a risk of interaction when the medication report was analyzed. These results call attention to the importance of the systematic adoption of PGx testing for our population and to the use of NGS data to extract pharmacogenetic variants. As important as the introduction of NGS technologies is the validation of such platforms for the call of pharmacogenetic variants. Studies on the economic evaluation of PGx implementation in Brazil are needed and should also include a discussion around the genotyping methodology.

## Data Availability

Publicly available datasets were analyzed in this study. These data can be found at https://abraom.ib.usp.br (genomic dataset published as a cohort and presented in short variants and frequencies); European Genome-phenome Archive (EGA) hosted by the EBI and the CRG, under EGA Study accession number EGAS00001005052 (individual-level sequence datasets and variant calling datasets).

## Footnotes

1
www.pharmgkb.org

2
www.pharmgkb.org/downloads

3
www.pharmgkb.org/page/varAnnScoring

4 abraom.ib.usp.br

5
www.pharmgkb.org/page/pgxGeneRef

6
www.pharmgkb.org/page/cyp2c19RefMaterials
