# Supplementary material for: Characterization of pharmacogenomic variants in a Brazilian admixed cohort of elderly individuals based on whole-genome sequencing data
Source: Front Pharmacol. 2023 May 10;14:1178715. doi: 10.3389/fphar.2023.1178715 (PMC10206227; doi:10.3389/fphar.2023.1178715)
Supplement: Supplementary file 1 [file Table1.DOCX]

***Supplementary Material***

**Characterization of pharmacogenomic variants in a Brazilian admixed cohort of elderly individuals** **based on whole-genome sequencing data**

**Luciana Bertholim-Nasciben, Marilia O. Scliar, Guilherme Debortoli, Bhooma Thiruvahindrapuram, Stephen W. Scherer, Yeda A. O. Duarte, Mayana Zatz, Guilherme Suarez-Kurtz, Esteban J. Parra, Michel S. Naslavsky^*^**

*** Correspondence:** Michel S. Naslavsky : mnaslavsky@usp.br

[**Supplementary Figure 1.** Study workflow.. 2](#_Toc128661282)

[**Supplementary Figure 2.** Examples of new star alleles with structural variation. 3](#_Toc128661283)

[**Supplementary Table 1**. Star alleles frequencies identified by Stargazer 1.0.7. 4](#_Toc128661284)

[**Supplemeyary Table 2.** Diplotypes frequencies identified by Stargazer 1.0.7. 12](#_Toc128661285)

[**Supplementary table 3.** Allele frequencies extracted from the WGS database (ABraOM). 28](#_Toc128661286)

[**Supplementary Table 4.** Cohort medication use and predicted high-risk individuals. 29](#_Toc128661287)

**
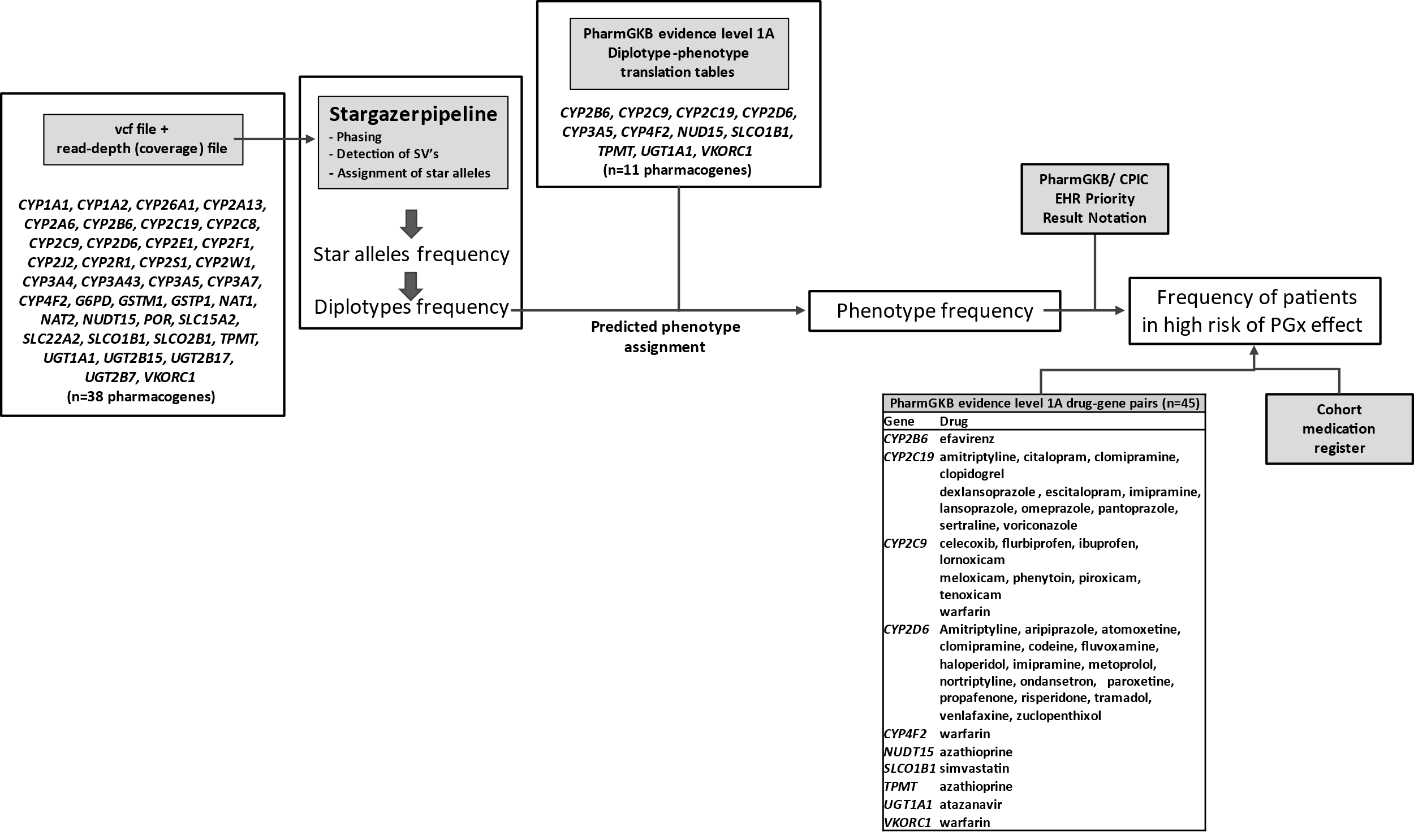
**

**Supplementary Figure 1.** Study workflow. Stargazer was used to call star alleles using the vcf and coverage files as input. Frequencies of star alleles and diplotypes were calculated. For eleven pharmacogenes with PharmGKB evidence level 1A, diplotypes-phenotype translation tables were used to map the individual predicted phenotype. Cohort medication register were checked against predicted phenotype for 45 gene-drug pairs to verify individuals at potential risk for gene-drug interaction.

**
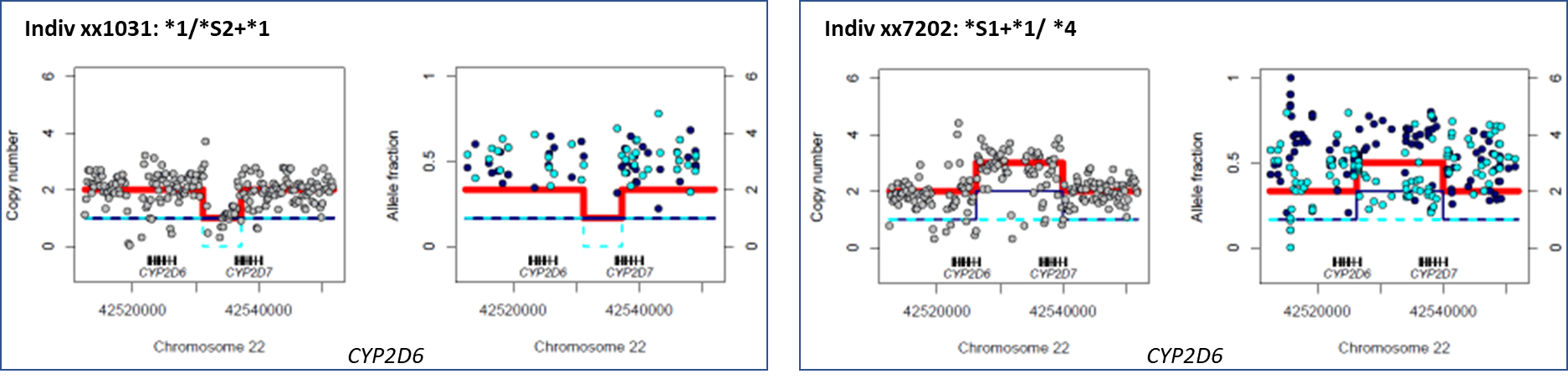
**

**Supplementary Figure 2.** Examples of new star alleles with structural variation. Gray points indicate the estimates of the number of copies of the sample calculated from read depth. The navy solid line and the cyan dotted line represent copy numbers profiles for each haplotype. The thick color lines represent copies of profiles of different genes for both haplotypes combined. Figures were output from Stargazer pipeline (Lee, Wheeler, Patterson, *et al.*, 2019; Lee, Wheeler, Thummel, *et al.*, 2019)**.**

**Supplementary Table 1**. Star alleles frequencies identified by Stargazer 1.0.7.

| **Gene** | **Star Allele** | **Presence of SVs** | **Activity Score^¥^** | **chr** | **Snv^£^** | **rsIDs** | **Star Allele Count** | **Star Allele Frequency** |
| --- | --- | --- | --- | --- | --- | --- | --- | --- |
| ***CYP1A1*** | *1 | no_sv | 1 | chr15 | ref |  | 1691 | 0.72203 |
| ***CYP1A1*** | *2A | no_sv | unknown | chr15 | 75011641:A>G | rs4646903 | 262 | 0.11187 |
| ***CYP1A1*** | *2B | no_sv | unknown | chr15 | 75011641:A>G, 75012985:T>C | rs4646903, rs1048943 | 227 | 0.09693 |
| ***CYP1A1*** | *4 | no_sv | unknown | chr15 | 75012987:G>T | rs1799814 | 123 | 0.05252 |
| ***CYP1A1*** | *2C | no_sv | 1 | chr15 | 75012985:T>C | rs1048943 | 12 | 0.00512 |
| ***CYP1A1*** | *13 | no_sv | unknown | chr15 | 75015305:C>T | rs4646422 | 10 | 0.00427 |
| ***CYP1A1*** | *DEL | cnv0 | 0 | chr15 | . |  | 10 | 0.00427 |
| ***CYP1A1*** | *1x2 | cnv2 | 2 | chr15 |  |  | 3 | 0.00128 |
| ***CYP1A1*** | *5 | no_sv | unknown | chr15 | 75012979:G>T, 75012979:G>T | rs41279188, rs41279188 | 2 | 0.00085 |
| ***CYP1A1*** | . | . | . | chr15 |  |  | 2 | 0.00085 |
| ***CYP1A2*** | *1F | no_sv | unknown | chr15 | 75041917:C>A | rs762551 | 1251 | 0.53416 |
| ***CYP1A2*** | *1 | no_sv | 1 | chr15 | ref |  | 754 | 0.32195 |
| ***CYP1A2*** | *1L | no_sv | unknown | chr15 | 75038220:G>A, 75039612:AT>A, 75041917:C>A, 75047426:T>C | rs2069514, rs35694136, rs762551, rs2470890 | 319 | 0.13621 |
| ***CYP1A2*** | *DEL | cnv0 | 0 | chr15 | . |  | 13 | 0.00555 |
| ***CYP1A2*** | *1C | no_sv | unknown | chr15 | 75038220:G>A | rs2069514 | 3 | 0.00128 |
| ***CYP1A2*** | *1x2 | cnv2 | 2 | chr15 |  |  | 1 | 0.00043 |
| ***CYP1A2*** | *1Fx2 | cnv2 | unknown | chr15 |  |  | 1 | 0.00043 |
| ***CYP2A6*** | *1 | no_sv | 1 | chr19 | ref |  | 1822 | 0.77797 |
| ***CYP2A6*** | *9 | no_sv | 0.5 | chr19 | 41356379:A>C | rs28399433 | 170 | 0.07259 |
| ***CYP2A6*** | *12 | gc_e1e2 | 0.5 | chr19 | . |  | 59 | 0.02519 |
| ***CYP2A6*** | *17 | no_sv | 0.5 | chr19 | 41351267:C>T | rs28399454 | 56 | 0.02391 |
| ***CYP2A6*** | *2 | no_sv | 0 | chr19 | 41354533:A>T | rs1801272 | 47 | 0.02007 |
| ***CYP2A6*** | *14 | no_sv | unknown | chr19 | 41356246:C>T | rs28399435 | 40 | 0.01708 |
| ***CYP2A6*** | *18 | no_sv | 0.5 | chr19 | 41350664:T>A | rs1809810 | 38 | 0.01623 |
| ***CYP2A6*** | *4 | cnv0 | 0 | chr19 | . |  | 35 | 0.01494 |
| ***CYP2A6*** | *21 | no_sv | 0.5 | chr19 | 41349759:T>C | rs6413474 | 19 | 0.00811 |
| ***CYP2A6*** | *1+*S2 | gc_e9 | unknown | chr19 |  |  | 16 | 0.00683 |
| ***CYP2A6*** | *1x2 | cnv2 | 2 | chr19 |  |  | 11 | 0.0047 |
| ***CYP2A6*** | *31 | no_sv | unknown | chr19 | 41356316:T>G | rs72549432 | 10 | 0.00427 |
| ***CYP2A6*** | *25 | no_sv | unknown | chr19 | 41354660:A>G | rs2839940 | 6 | 0.00256 |
| ***CYP2A6*** | *20 | no_sv | 0 | chr19 | 41354189:CTT>C | rs568811809 | 5 | 0.00213 |
| ***CYP2A6*** | *23 | no_sv | 0.5 | chr19 | 41354171:G>A, 41354171:G>A | rs56256500, rs56256500 | 3 | 0.00128 |
| ***CYP2A6*** | *1+*S6 | dup7b | unknown | chr19 |  |  | 2 | 0.00085 |
| ***CYP2A6*** | *25x2 | cnv2 | unknown | chr19 |  |  | 2 | 0.00085 |
| ***CYP2A6*** | *26 | no_sv | 0.5 | chr19 | 41354621:A>C, 41354629:C>A, 41354629:C>A, 41354660:A>G | rs59552350, rs4986891, rs4986891, rs2839940 | 1 | 0.00043 |
| ***CYP2A13*** | *1 | no_sv | 1 | chr19 | ref |  | 2159 | 0.92186 |
| ***CYP2A13*** | *2 | no_sv | unknown | chr19 | 41594450:G>A, 41597751:C>T | rs8192784, rs8192789 | 107 | 0.04569 |
| ***CYP2A13*** | *8 | no_sv | unknown | chr19 | 41596082:C>G | rs112337232 | 40 | 0.01708 |
| ***CYP2A13*** | *7 | no_sv | 0 | chr19 | 41594954:C>T | rs72552266 | 21 | 0.00897 |
| ***CYP2A13*** | *DEL | cnv0 | 0 | chr19 | . |  | 11 | 0.0047 |
| ***CYP2A13*** | *1x2 | cnv2 | 2 | chr19 |  |  | 4 | 0.00171 |
| ***CYP2B6*** | *1 | no_sv | 1 | chr19 | ref |  | 1220 | 0.52092 |
| ***CYP2B6*** | *6 | no_sv | 0.5 | chr19 | 41512841:G>T, 41515263:A>G | rs3745274, rs2279343 | 671 | 0.28651 |
| ***CYP2B6*** | *5 | no_sv | 1 | chr19 | 41522715:C>T, 41522715:C>T | rs3211371, rs3211371 | 161 | 0.06874 |
| ***CYP2B6*** | *2 | no_sv | 1 | chr19 | 41497274:C>T | rs8192709 | 126 | 0.0538 |
| ***CYP2B6*** | *4 | no_sv | 1.5 | chr19 | 41515263:A>G | rs2279343 | 38 | 0.01623 |
| ***CYP2B6*** | *22 | no_sv | 1.5 | chr19 | 41497129:T>C | rs34223104 | 38 | 0.01623 |
| ***CYP2B6*** | *18 | no_sv | 0 | chr19 | 41518221:T>C | rs28399499 | 36 | 0.01537 |
| ***CYP2B6*** | *10 | no_sv | unknown | chr19 | 41497272:A>T, 41497274:C>T, 41509950:G>C | rs34883432, rs8192709, rs2279341 | 23 | 0.00982 |
| ***CYP2B6*** | *9 | no_sv | 0.5 | chr19 | 41512841:G>T | rs3745274 | 9 | 0.00384 |
| ***CYP2B6*** | *15 | no_sv | unknown | chr19 | 41518598:T>A | rs35979566 | 8 | 0.00342 |
| ***CYP2B6*** | *13 | no_sv | 0 | chr19 | 41510282:A>G, 41512841:G>T, 41515263:A>G | rs12721655, rs3745274, rs2279343 | 4 | 0.00171 |
| ***CYP2B6*** | *11 | no_sv | unknown | chr19 | 41497346:A>G | rs35303484 | 3 | 0.00128 |
| ***CYP2B6*** | *29 | gc_i4e9 | 0.5 | chr19 | . |  | 2 | 0.00085 |
| ***CYP2B6*** | *8 | no_sv | 0 | chr19 | 41510282:A>G | rs12721655 | 1 | 0.00043 |
| ***CYP2B6*** | *22x2 | cnv2 | 3 | chr19 |  |  | 1 | 0.00043 |
| ***CYP2B6*** | *36 | no_sv | 0.5 | chr19 | 41497129:T>C, 41512841:G>T, 41515263:A>G | rs34223104, rs3745274, rs2279343 | 1 | 0.00043 |
| ***CYP2C8*** | *1 | no_sv | 1 | chr10 | ref |  | 1905 | 0.81341 |
| ***CYP2C8*** | *3 | no_sv | 0.5 | chr10 | 96798749:T>C, 96827030:C>T | rs10509681, rs11572080 | 246 | 0.10504 |
| ***CYP2C8*** | *4 | no_sv | 0.5 | chr10 | 96818119:G>C | rs1058930 | 91 | 0.03886 |
| ***CYP2C8*** | *2 | no_sv | 0.5 | chr10 | 96818106:T>A | rs11572103 | 83 | 0.03544 |
| ***CYP2C8*** | *1x2 | cnv2 | 2 | chr10 |  |  | 13 | 0.00555 |
| ***CYP2C8*** | *2x2 | cnv2 | 1 | chr10 |  |  | 1 | 0.00043 |
| ***CYP2C8*** | *3x2 | cnv2 | 1 | chr10 |  |  | 1 | 0.00043 |
| ***CYP2C8*** | *7 | no_sv | 0 | chr10 | 96824643:G>A, 96824643:G>A | rs72558195, rs72558195 | 1 | 0.00043 |
| ***CYP2C8*** | *DEL | cnv0 | 0 | chr10 | . |  | 1 | 0.00043 |
| ***CYP2C9*** | *1 | no_sv | 1 | chr10 | ref |  | 1852 | 0.79078 |
| ***CYP2C9*** | *2 | no_sv | 0.5 | chr10 | 96702047:C>T | rs1799853 | 264 | 0.11272 |
| ***CYP2C9*** | *3 | no_sv | 0.5 | chr10 | 96741053:A>C, 96748737:A>T | rs1057910, rs1057911 | 121 | 0.05167 |
| ***CYP2C9*** | *9 | no_sv | 1 | chr10 | 96708974:A>G | rs2256871 | 45 | 0.01921 |
| ***CYP2C9*** | *8 | no_sv | 0.5 | chr10 | 96702066:G>A, 96702066:G>A | rs7900194, rs7900194 | 23 | 0.00982 |
| ***CYP2C9*** | *11 | no_sv | 0.5 | chr10 | 96740981:C>T | rs28371685 | 14 | 0.00598 |
| ***CYP2C9*** | *5 | no_sv | 0.5 | chr10 | 96741058:C>G | rs28371686 | 9 | 0.00384 |
| ***CYP2C9*** | *1x2 | cnv2 | 2 | chr10 |  |  | 7 | 0.00299 |
| ***CYP2C9*** | *36 | no_sv | 0 | chr10 | 96698440:A>G | rs114071557 | 2 | 0.00085 |
| ***CYP2C9*** | *1x3 | cnv3 | 3 | chr10 |  |  | 1 | 0.00043 |
| ***CYP2C9*** | *3x2 | cnv2 | 1 | chr10 |  |  | 1 | 0.00043 |
| ***CYP2C9*** | *6 | no_sv | 0 | chr10 | 96709038:GA>G | rs9332131 | 1 | 0.00043 |
| ***CYP2C9*** | *12 | no_sv | 0.5 | chr10 | 96748777:C>T | rs9332239 | 1 | 0.00043 |
| ***CYP2C9*** | *DEL | cnv0 | 0 | chr10 | . |  | 1 | 0.00043 |
| ***CYP2C19*** | *1 | no_sv | 1 | chr10 | ref |  | 1471 | 0.6281 |
| ***CYP2C19*** | *17 | no_sv | 1.5 | chr10 | 96519061:C>T, 96521657:C>T, 96602623:A>G | rs11188072, rs12248560, rs3758581 | 456 | 0.19471 |
| ***CYP2C19*** | *2 | no_sv | 0 | chr10 | 96535124:A>G, 96541616:G>A | rs12769205, rs4244285 | 350 | 0.14944 |
| ***CYP2C19*** | *35 | no_sv | 0 | chr10 | 96535124:A>G | rs12769205 | 18 | 0.00769 |
| ***CYP2C19*** | *9 | no_sv | 0.5 | chr10 | 96535246:G>A | rs17884712 | 13 | 0.00555 |
| ***CYP2C19*** | *3 | no_sv | 0 | chr10 | 96540410:G>A | rs4986893 | 8 | 0.00342 |
| ***CYP2C19*** | *15 | no_sv | 1 | chr10 | 96522517:A>C | rs17882687 | 7 | 0.00299 |
| ***CYP2C19*** | *4 | no_sv | 0 | chr10 | 96522463:A>G | rs28399504 | 6 | 0.00256 |
| ***CYP2C19*** | *8 | no_sv | 0 | chr10 | 96535173:T>C | rs41291556 | 6 | 0.00256 |
| ***CYP2C19*** | *13 | no_sv | 1 | chr10 | 96602623:A>G, 96609752:C>T | rs3758581, rs17879685 | 3 | 0.00128 |
| ***CYP2C19*** | *12 | no_sv | 0.98 | chr10 | 96612671:A>C | rs55640102 | 2 | 0.00085 |
| ***CYP2C19*** | *5 | no_sv | 0 | chr10 | 96612495:C>T | rs56337013 | 1 | 0.00043 |
| ***CYP2C19*** | *10 | no_sv | 0.5 | chr10 | 96541615:C>T | rs6413438 | 1 | 0.00043 |
| ***CYP2D6*** | *1 | no_sv | 1 | chr22 | ref |  | 828 | 0.35354 |
| ***CYP2D6*** | *2 | no_sv | 1 | chr22 | 42522613:C>G, 42523943:G>A, 42525132:C>G | rs1135840, rs16947, rs1058164 | 390 | 0.16652 |
| ***CYP2D6*** | *4 | no_sv | 0 | chr22 | 42524947:C>T | rs3892097 | 214 | 0.09137 |
| ***CYP2D6*** | *41 | no_sv | 0.5 | chr22 | 42523805:C>T | rs28371725 | 175 | 0.07472 |
| ***CYP2D6*** | *68+*4 | gc_i1e9 | 0 | chr22 |  |  | 105 | 0.04483 |
| ***CYP2D6*** | *17 | no_sv | 0.5 | chr22 | 42525772:G>A, 42525772:G>A | rs28371706, rs28371706 | 100 | 0.0427 |
| ***CYP2D6*** | *35 | no_sv | 1 | chr22 | 42522613:C>G, 42523943:G>A, 42525132:C>G, 42526763:C>T | rs1135840, rs16947, rs1058164, rs769258 | 85 | 0.03629 |
| ***CYP2D6*** | *5 | cnv0 | 0 | chr22 | . |  | 71 | 0.03032 |
| ***CYP2D6*** | *10 | no_sv | 0.25 | chr22 | 42526694:G>A | rs1065852 | 54 | 0.02306 |
| ***CYP2D6*** | *29 | no_sv | 0.5 | chr22 | 42522613:C>G, 42523610:C>T, 42523943:G>A, 42525134:C>T | rs1135840, rs59421388, rs16947, rs61736512 | 48 | 0.0205 |
| ***CYP2D6*** | *9 | no_sv | 0.5 | chr22 | 42524175:CCTT>C | rs5030656 | 47 | 0.02007 |
| ***CYP2D6*** | *2x2 | cnv2 | 2 | chr22 |  |  | 40 | 0.01708 |
| ***CYP2D6*** | *36+*10 | gc_e9 | 0.25 | chr22 |  |  | 19 | 0.00811 |
| ***CYP2D6*** | *3 | no_sv | 0 | chr22 | 42524243:CT>C | rs35742686 | 19 | 0.00811 |
| ***CYP2D6*** | *6 | no_sv | 0 | chr22 | 42525085:CA>C | rs5030655 | 15 | 0.0064 |
| ***CYP2D6*** | *28 | no_sv | unknown | chr22 | 42522613:C>G, 42523943:G>A, 42525089:G>C, 42526775:C>T | rs1135840, rs16947, rs78482768, rs72549358 | 15 | 0.0064 |
| ***CYP2D6*** | *1x2 | cnv2 | 2 | chr22 |  |  | 14 | 0.00598 |
| ***CYP2D6*** | *43 | no_sv | unknown | chr22 | 42526717:C>T | rs28371696 | 12 | 0.00512 |
| ***CYP2D6*** | *4x2 | cnv2 | 0 | chr22 |  |  | 10 | 0.00427 |
| ***CYP2D6*** | *33 | no_sv | 1 | chr22 | 42524310:C>A | rs28371717 | 9 | 0.00384 |
| ***CYP2D6*** | *20 | no_sv | 0 | chr22 | 42524820:T>TC | rs72549354 | 9 | 0.00384 |
| ***CYP2D6*** | . | . | . | chr22 |  |  | 8 | 0.00342 |
| ***CYP2D6*** | *S1+*1 | gc_i1e9 | 1 | chr22 |  |  | 7 | 0.00299 |
| ***CYP2D6*** | *106 | no_sv | unknown | chr22 | 42522916:C>T, 42522916:C>T | rs28371733, rs28371733 | 6 | 0.00256 |
| ***CYP2D6*** | *4N+*4 | gc_e9 | 0 | chr22 |  |  | 4 | 0.00171 |
| ***CYP2D6*** | *2x3 | cnv3 | 3 | chr22 |  |  | 4 | 0.00171 |
| ***CYP2D6*** | *84 | no_sv | 0.5 | chr22 | 42524219:G>T | rs148769737 | 3 | 0.00128 |
| ***CYP2D6*** | *29x2 | cnv2 | 1 | chr22 |  |  | 3 | 0.00128 |
| ***CYP2D6*** | *59 | no_sv | 0.5 | chr22 | 42523854:C>T | rs79292917 | 3 | 0.00128 |
| ***CYP2D6*** | *46 | no_sv | 1 | chr22 | 42522613:C>G, 42523943:G>A, 42525077:C>T, 42526717:C>T | rs1135840, rs16947, rs28371710, rs28371696 | 3 | 0.00128 |
| ***CYP2D6*** | *41x2 | cnv2 | 1 | chr22 |  |  | 2 | 0.00085 |
| ***CYP2D6*** | *83+*2 | gc_e9 | 1 | chr22 |  |  | 2 | 0.00085 |
| ***CYP2D6*** | *2D | no_sv | 1 | chr22 | 42522613:C>G, 42523943:G>A | rs1135840, rs16947 | 2 | 0.00085 |
| ***CYP2D6*** | *41x3 | cnv3 | 1.5 | chr22 |  |  | 2 | 0.00085 |
| ***CYP2D6*** | *13B | gc_7to6_i1 | 0 | chr22 | . |  | 2 | 0.00085 |
| ***CYP2D6*** | *S2+*1 | del1 | 1 | chr22 |  |  | 2 | 0.00085 |
| ***CYP2D6*** | *31 | no_sv | 0 | chr22 | 42522751:C>T | rs267608319 | 2 | 0.00085 |
| ***CYP2D6*** | *35x2 | cnv2 | 2 | chr22 |  |  | 1 | 0.00043 |
| ***CYP2D6*** | *34 | no_sv | 1 | chr22 | 42523943:G>A | rs16947 | 1 | 0.00043 |
| ***CYP2D6*** | *7 | no_sv | 0 | chr22 | 42523858:T>G | rs5030867 | 1 | 0.00043 |
| ***CYP2D6*** | *1x3 | cnv3 | 3 | chr22 |  |  | 1 | 0.00043 |
| ***CYP2D6*** | *10x2 | cnv2 | 0.5 | chr22 |  |  | 1 | 0.00043 |
| ***CYP2D6*** | *13C | gc_e1e7 | 0 | chr22 | . |  | 1 | 0.00043 |
| ***CYP2D6*** | *21x2 | cnv2 | 0 | chr22 |  |  | 1 | 0.00043 |
| ***CYP2D6*** | *40 | no_sv | 0 | chr22 | 42524929:T>TGGGGCGAAAGGGGCGAAA, 42524929:T>TGGGGCGAAAGGGGCGAAA, 42525772:G>A, 42525772:G>A | rs553846709, rs553846709, rs28371706, rs28371706 | 1 | 0.00043 |
| ***CYP2E1*** | *1 | no_sv | 1 | chr10 | ref |  | 1527 | 0.65201 |
| ***CYP2E1*** | *7 | no_sv | 0.5 | chr10 | 135340567:T>A | rs2070673 | 684 | 0.29206 |
| ***CYP2E1*** | . | . | . | chr10 |  |  | 46 | 0.01964 |
| ***CYP2E1*** | *7x2 | cnv2 | 1 | chr10 |  |  | 31 | 0.01324 |
| ***CYP2E1*** | *1x2 | cnv2 | 2 | chr10 |  |  | 28 | 0.01196 |
| ***CYP2E1*** | *S1 | dup_e7e9 | 0.5 | chr10 | 135345675:G>A | rs6413419 | 12 | 0.00512 |
| ***CYP2E1*** | *4 | no_sv | 1 | chr10 | 135345675:G>A | rs6413419 | 5 | 0.00213 |
| ***CYP2E1*** | *7x3 | cnv3 | 1.5 | chr10 |  |  | 5 | 0.00213 |
| ***CYP2E1*** | *DEL | cnv0 | 0 | chr10 | . |  | 3 | 0.00128 |
| ***CYP2E1*** | *1x3 | cnv3 | 3 | chr10 |  |  | 1 | 0.00043 |
| ***CYP2F1*** | *1 | no_sv | 1 | chr19 | ref |  | 1499 | 0.64005 |
| ***CYP2F1*** | *2 | no_sv | 0 | chr19 | 41622107:G>GC | rs3833221 | 496 | 0.21178 |
| ***CYP2F1*** | *5 | no_sv | unknown | chr19 | 41631417:T>C | rs144315434 | 175 | 0.07472 |
| ***CYP2F1*** | *4 | no_sv | unknown | chr19 | 41622205:T>C, 41627868:G>A | rs58285195, rs305974 | 121 | 0.05167 |
| ***CYP2F1*** | *DEL | cnv0 | 0 | chr19 | . |  | 27 | 0.01153 |
| ***CYP2F1*** | *6 | no_sv | unknown | chr19 | 41622481:G>C | rs57670668 | 22 | 0.00939 |
| ***CYP2F1*** | *1x2 | cnv2 | 2 | chr19 |  |  | 2 | 0.00085 |
| ***CYP2J2*** | *1 | no_sv | 1 | chr1 | ref |  | 2163 | 0.92357 |
| ***CYP2J2*** | *7 | no_sv | unknown | chr1 | 60392494:C>A | rs890293 | 164 | 0.07003 |
| ***CYP2J2*** | *1x2 | cnv2 | 2 | chr1 |  |  | 10 | 0.00427 |
| ***CYP2J2*** | *5 | no_sv | unknown | chr1 | 60370710:C>T | rs56053398 | 4 | 0.00171 |
| ***CYP2J2*** | *7x2 | cnv2 | unknown | chr1 |  |  | 1 | 0.00043 |
| ***CYP2R1*** | *1 | no_sv | 1 | chr11 | ref |  | 2340 | 0.99915 |
| ***CYP2R1*** | *2 | no_sv | unknown | chr11 | 14907393:A>G | rs61495246 | 1 | 0.00043 |
| ***CYP2R1*** | *DEL | cnv0 | 0 | chr11 | . |  | 1 | 0.00043 |
| ***CYP2S1*** | *1 | no_sv | 1 | chr19 | ref |  | 2265 | 0.96712 |
| ***CYP2S1*** | *3 | no_sv | unknown | chr19 | 41712275:C>T | rs34971233 | 55 | 0.02348 |
| ***CYP2S1*** | *DEL | cnv0 | 0 | chr19 | . |  | 19 | 0.00811 |
| ***CYP2S1*** | *1x2 | cnv2 | 2 | chr19 |  |  | 3 | 0.00128 |
| ***CYP2W1*** | *1 | no_sv | 1 | chr7 | ref |  | 1674 | 0.71477 |
| ***CYP2W1*** | *6 | no_sv | unknown | chr7 | 1028448:C>T | rs3808348 | 350 | 0.14944 |
| ***CYP2W1*** | *2 | no_sv | unknown | chr7 | 1024855:G>A | rs3735684 | 195 | 0.08326 |
| ***CYP2W1*** | *DEL | cnv0 | 0 | chr7 | . |  | 97 | 0.04142 |
| ***CYP2W1*** | *4 | no_sv | unknown | chr7 | 1028279:G>A | rs78873069 | 24 | 0.01025 |
| ***CYP2W1*** | *1x2 | cnv2 | 2 | chr7 |  |  | 1 | 0.00043 |
| ***CYP2W1*** | *2x2 | cnv2 | unknown | chr7 |  |  | 1 | 0.00043 |
| ***CYP3A4*** | *1 | no_sv | 1 | chr7 | ref |  | 1902 | 0.81213 |
| ***CYP3A4*** | *1B | no_sv | unknown | chr7 | 99382096:T>C | rs2740574 | 370 | 0.15798 |
| ***CYP3A4*** | *22 | no_sv | 0.5 | chr7 | 99366316:G>A | rs35599367 | 49 | 0.02092 |
| ***CYP3A4*** | *3 | no_sv | unknown | chr7 | 99358524:A>G | rs4986910 | 7 | 0.00299 |
| ***CYP3A4*** | *1x2 | cnv2 | 2 | chr7 |  |  | 4 | 0.00171 |
| ***CYP3A4*** | *10 | no_sv | unknown | chr7 | 99367392:C>G | rs4986908 | 3 | 0.00128 |
| ***CYP3A4*** | *1Bx2 | cnv2 | unknown | chr7 |  |  | 2 | 0.00085 |
| ***CYP3A4*** | *12 | no_sv | 0.5 | chr7 | 99359800:G>A | rs12721629 | 2 | 0.00085 |
| ***CYP3A4*** | *18 | no_sv | unknown | chr7 | 99361626:A>G | rs28371759 | 1 | 0.00043 |
| ***CYP3A4*** | *20 | no_sv | 0 | chr7 | 99355806:G>GT | rs67666821 | 1 | 0.00043 |
| ***CYP3A4*** | *DEL | cnv0 | 0 | chr7 | . |  | 1 | 0.00043 |
| ***CYP3A5*** | *3 | no_sv | 0 | chr7 | 99270539:T>C | rs776746 | 1787 | 0.76302 |
| ***CYP3A5*** | *1 | no_sv | 1 | chr7 | ref |  | 427 | 0.18232 |
| ***CYP3A5*** | *6 | no_sv | 0 | chr7 | 99262835:C>T | rs10264272 | 71 | 0.03032 |
| ***CYP3A5*** | *7 | no_sv | 0 | chr7 | 99250393:T>TA | rs41303343 | 51 | 0.02178 |
| ***CYP3A5*** | *3x2 | cnv2 | 0 | chr7 |  |  | 3 | 0.00128 |
| ***CYP3A5*** | *1x2 | cnv2 | 2 | chr7 |  |  | 1 | 0.00043 |
| ***CYP3A5*** | *6x2 | cnv2 | 0 | chr7 |  |  | 1 | 0.00043 |
| ***CYP3A5*** | *DEL | cnv0 | 0 | chr7 | . |  | 1 | 0.00043 |
| ***CYP3A7*** | *1 | no_sv | 1 | chr7 | ref |  | 1822 | 0.77797 |
| ***CYP3A7*** | *2 | no_sv | unknown | chr7 | 99306685:G>C | rs2257401 | 446 | 0.19044 |
| ***CYP3A7*** | *1E | no_sv | unknown | chr7 | 99332765:C>T | rs28451617 | 53 | 0.02263 |
| ***CYP3A7*** | *1D | no_sv | unknown | chr7 | 99332807:C>T | rs55798860 | 11 | 0.0047 |
| ***CYP3A7*** | *1B | no_sv | unknown | chr7 | 99333030:G>A | rs45465393 | 4 | 0.00171 |
| ***CYP3A7*** | *1x2 | cnv2 | 2 | chr7 |  |  | 3 | 0.00128 |
| ***CYP3A7*** | *2x2 | cnv2 | unknown | chr7 |  |  | 2 | 0.00085 |
| ***CYP3A7*** | *DEL | cnv0 | 0 | chr7 | . |  | 1 | 0.00043 |
| ***CYP3A43*** | *1 | no_sv | 1 | chr7 | ref |  | 1873 | 0.79974 |
| ***CYP3A43*** | *2B | no_sv | 0 | chr7 | 99434077:TA>T, 99457605:C>G | rs61469810, rs680055 | 180 | 0.07686 |
| ***CYP3A43*** | *1B | no_sv | unknown | chr7 | 99459256:C>T | rs17342647 | 129 | 0.05508 |
| ***CYP3A43*** | *3 | no_sv | unknown | chr7 | 99457605:C>G | rs680055 | 105 | 0.04483 |
| ***CYP3A43*** | *2A | no_sv | 0 | chr7 | 99434077:TA>T | rs61469810 | 48 | 0.0205 |
| ***CYP3A43*** | *1x2 | cnv2 | 2 | chr7 |  |  | 3 | 0.00128 |
| ***CYP3A43*** | *1Bx2 | cnv2 | unknown | chr7 |  |  | 1 | 0.00043 |
| ***CYP3A43*** | *2Bx2 | cnv2 | 0 | chr7 |  |  | 1 | 0.00043 |
| ***CYP3A43*** | *3x2 | cnv2 | unknown | chr7 |  |  | 1 | 0.00043 |
| ***CYP3A43*** | *DEL | cnv0 | 0 | chr7 | . |  | 1 | 0.00043 |
| ***CYP4F2*** | *1 | no_sv | 1 | chr19 | ref |  | 1527 | 0.65201 |
| ***CYP4F2*** | *3 | no_sv | 0.5 | chr19 | 15990431:C>T | rs2108622 | 681 | 0.29078 |
| ***CYP4F2*** | *2 | no_sv | 1 | chr19 | 16008388:A>C | rs3093105 | 126 | 0.0538 |
| ***CYP4F2*** | *1x2 | cnv2 | 2 | chr19 |  |  | 3 | 0.00128 |
| ***CYP4F2*** | *3x2 | cnv2 | 1 | chr19 |  |  | 2 | 0.00085 |
| ***CYP4F2*** | *DEL | cnv0 | 0 | chr19 | . |  | 2 | 0.00085 |
| ***CYP4F2*** | *2x2 | cnv2 | 2 | chr19 |  |  | 1 | 0.00043 |
| ***CYP26A1*** | *1 | no_sv | 1 | chr10 | ref |  | 2315 | 0.98847 |
| ***CYP26A1*** | *2 | no_sv | unknown | chr10 | 94834638:C>A | rs61735552 | 27 | 0.01153 |
| ***G6PD*** | *1 | no_sv | 1 | chrX | ref |  | 1752 | 0.74808 |
| ***G6PD*** | *DEL | cnv0 | 0 | chrX | . |  | 428 | 0.18275 |
| ***G6PD*** | *2 | no_sv | 1 | chrX | 153763492:T>C | rs1050829 | 81 | 0.03459 |
| ***G6PD*** | *3 | no_sv | 0.5 | chrX | 153763492:T>C, 153764217:C>T | rs1050829, rs1050828 | 77 | 0.03288 |
| ***G6PD*** | *2x2 | cnv2 | 2 | chrX |  |  | 1 | 0.00043 |
| ***G6PD*** | *5 | no_sv | 0.5 | chrX | 153761240:A>G, 153763492:T>C | rs76723693, rs1050829 | 1 | 0.00043 |
| ***G6PD*** | *29 | no_sv | 0.5 | chrX | 153761259:C>T | rs137852339 | 1 | 0.00043 |
| ***G6PD*** | *179 | no_sv | unknown | chrX | 153763391:C>G | rs370918918 | 1 | 0.00043 |
| ***GSTM1*** | *2 | cnv0 | 0 | chr1 | . |  | 1527 | 0.65201 |
| ***GSTM1*** | *1 | no_sv | 1 | chr1 | ref |  | 549 | 0.23442 |
| ***GSTM1*** | *3 | no_sv | 1 | chr1 | 110233138:G>C | rs1065411 | 261 | 0.11144 |
| ***GSTM1*** | *1x2 | cnv2 | 2 | chr1 |  |  | 4 | 0.00171 |
| ***GSTM1*** | *3x2 | cnv2 | 2 | chr1 |  |  | 1 | 0.00043 |
| ***GSTP1*** | *1 | no_sv | 1 | chr11 | ref |  | 1454 | 0.62084 |
| ***GSTP1*** | *2 | no_sv | unknown | chr11 | 67352689:A>G | rs1695 | 766 | 0.32707 |
| ***GSTP1*** | *3 | no_sv | unknown | chr11 | 67352689:A>G, 67353579:C>T | rs1695, rs1138272 | 86 | 0.03672 |
| ***GSTP1*** | *DEL | cnv0 | 0 | chr11 | . |  | 28 | 0.01196 |
| ***GSTP1*** | *4 | no_sv | unknown | chr11 | 67353579:C>T | rs1138272 | 5 | 0.00213 |
| ***GSTP1*** | *1x2 | cnv2 | 2 | chr11 |  |  | 3 | 0.00128 |
| ***NAT1*** | *1 | no_sv | 1 | chr8 | ref |  | 1513 | 0.64603 |
| ***NAT1*** | *10 | no_sv | unknown | chr8 | 18080644:T>A, 18080651:C>A | rs1057126, rs15561 | 679 | 0.28992 |
| ***NAT1*** | *11 | no_sv | unknown | chr8 | 18079213:C>T, 18079517:A>T, 18080015:G>A, 18080196:T>G, 18080635:TAATAATAAA>T | rs4986988, rs4986989, rs4986990, rs4986783, rs367921464 | 40 | 0.01708 |
| ***NAT1*** | *3 | no_sv | unknown | chr8 | 18080651:C>A | rs15561 | 37 | 0.0158 |
| ***NAT1*** | *14 | no_sv | 0.5 | chr8 | 18080116:G>A | rs4986782 | 31 | 0.01324 |
| ***NAT1*** | *15 | no_sv | 0 | chr8 | 18080115:C>T | rs5030839 | 14 | 0.00598 |
| ***NAT1*** | *27 | no_sv | 1 | chr8 | 18079577:T>G, 18080333:T>C | rs4986992, rs4986991 | 7 | 0.00299 |
| ***NAT1*** | *17 | no_sv | 0.5 | chr8 | 18079746:C>T | rs56379106 | 5 | 0.00213 |
| ***NAT1*** | *10x2 | cnv2 | unknown | chr8 |  |  | 3 | 0.00128 |
| ***NAT1*** | *19 | no_sv | 0 | chr8 | 18079653:C>T | rs56318881 | 3 | 0.00128 |
| ***NAT1*** | *22 | no_sv | 0.5 | chr8 | 18080308:A>T | rs56172717 | 3 | 0.00128 |
| ***NAT1*** | *30 | no_sv | unknown | chr8 | 18080001:G>A | rs4987076 | 3 | 0.00128 |
| ***NAT1*** | *1x2 | cnv2 | 2 | chr8 |  |  | 2 | 0.00085 |
| ***NAT1*** | *23 | no_sv | 1 | chr8 | 18080333:T>C | rs4986991 | 1 | 0.00043 |
| ***NAT1*** | *DEL | cnv0 | 0 | chr8 | . |  | 1 | 0.00043 |
| ***NAT2*** | *5 | no_sv | 0.5 | chr8 | 18257854:T>C | rs1801280 | 969 | 0.41375 |
| ***NAT2*** | *1 | no_sv | 1 | chr8 | ref |  | 564 | 0.24082 |
| ***NAT2*** | *6 | no_sv | 0.5 | chr8 | 18258103:G>A | rs1799930 | 564 | 0.24082 |
| ***NAT2*** | *7 | no_sv | 0.5 | chr8 | 18258370:G>A | rs1799931 | 98 | 0.04184 |
| ***NAT2*** | *12 | no_sv | 1 | chr8 | 18258316:A>G | rs1208 | 65 | 0.02775 |
| ***NAT2*** | *13 | no_sv | 1 | chr8 | 18257795:C>T | rs1041983 | 37 | 0.0158 |
| ***NAT2*** | *14 | no_sv | 0.5 | chr8 | 18257704:G>A | rs1801279 | 32 | 0.01366 |
| ***NAT2*** | *1x2 | cnv2 | 2 | chr8 |  |  | 4 | 0.00171 |
| ***NAT2*** | *5x2 | cnv2 | 1 | chr8 |  |  | 3 | 0.00128 |
| ***NAT2*** | *11 | no_sv | 1 | chr8 | 18257994:C>T | rs1799929 | 2 | 0.00085 |
| ***NAT2*** | *DEL | cnv0 | 0 | chr8 | . |  | 2 | 0.00085 |
| ***NAT2*** | *6x2 | cnv2 | 1 | chr8 |  |  | 1 | 0.00043 |
| ***NAT2*** | *14x2 | cnv2 | 1 | chr8 |  |  | 1 | 0.00043 |
| ***NUDT15*** | *1 | no_sv | 1 | chr13 | ref |  | 2300 | 0.98207 |
| ***NUDT15*** | *3 | no_sv | 0.25 | chr13 | 48619855:C>T | rs116855232 | 27 | 0.01153 |
| ***NUDT15*** | *4 | no_sv | 0.25 | chr13 | 48619856:G>A | rs147390019 | 11 | 0.0047 |
| ***NUDT15*** | *DEL | cnv0 | 0 | chr13 | . |  | 3 | 0.00128 |
| ***NUDT15*** | *1x2 | cnv2 | 2 | chr13 |  |  | 1 | 0.00043 |
| ***POR*** | *1 | no_sv | 1 | chr7 | ref |  | 1773 | 0.75705 |
| ***POR*** | *28 | no_sv | 0.5 | chr7 | 75615006:C>T | rs1057868 | 563 | 0.24039 |
| ***POR*** | *DEL | cnv0 | 0 | chr7 | . |  | 4 | 0.00171 |
| ***POR*** | *1x2 | cnv2 | 2 | chr7 |  |  | 2 | 0.00085 |
| ***SLC15A2*** | *1 | no_sv | 1 | chr3 | ref |  | 1379 | 0.58881 |
| ***SLC15A2*** | *2 | no_sv | unknown | chr3 | 121643804:C>T, 121647286:C>T, 121648168:G>A | rs2257212, rs1143671, rs1143672 | 961 | 0.41033 |
| ***SLC15A2*** | *2 | no_sv | unknown | chr3 | 121643804:C>T, 121647286:C>T, 121648168:G>A | rs2257212, rs1143671, rs1143672 | 961 | 0.41033 |
| ***SLC15A2*** | *DEL | cnv0 | 0 | chr3 | . |  | 2 | 0.00085 |
| ***SLC22A2*** | *1 | no_sv | 1 | chr6 | ref |  | 935 | 0.39923 |
| ***SLC22A2*** | *2 | no_sv | unknown | chr6 | 160679400:C>A | rs624249 | 844 | 0.36038 |
| ***SLC22A2*** | *3 | no_sv | unknown | chr6 | 160645832:T>C | rs316003 | 518 | 0.22118 |
| ***SLC22A2*** | *S1 | del_i9 | unknown | chr6 | 160663420:T>G | rs8177517 | 16 | 0.00683 |
| ***SLC22A2*** | *S2 | del_e11 | unknown | chr6 | . |  | 9 | 0.00384 |
| ***SLC22A2*** | *6 | no_sv | unknown | chr6 | 160670282:C>A | rs316019 | 9 | 0.00384 |
| ***SLC22A2*** | *7 | no_sv | unknown | chr6 | 160664685:G>A | rs8177516 | 8 | 0.00342 |
| ***SLC22A2*** | . | . | . | chr6 |  |  | 2 | 0.00085 |
| ***SLC22A2*** | *4 | no_sv | unknown | chr6 | 160663469:A>G | rs8177518 | 1 | 0.00043 |
| ***SLCO1B1*** | *1 | no_sv | 1 | chr12 | ref |  | 1004 | 0.42869 |
| ***SLCO1B1*** | *1B | no_sv | 1 | chr12 | 21329738:A>G | rs2306283 | 450 | 0.19214 |
| ***SLCO1B1*** | *14 | no_sv | 1.5 | chr12 | 21329738:A>G, 21329813:C>A | rs2306283, rs11045819 | 308 | 0.13151 |
| ***SLCO1B1*** | *15 | no_sv | 0.5 | chr12 | 21329738:A>G, 21331549:T>C | rs2306283, rs4149056 | 237 | 0.1012 |
| ***SLCO1B1*** | *35 | no_sv | 1.5 | chr12 | 21329738:A>G, 21391976:A>C | rs2306283, rs34671512 | 135 | 0.05764 |
| ***SLCO1B1*** | *21 | no_sv | unknown | chr12 | 21283322:G>A, 21329738:A>G, 21331625:C>T | rs4149015, rs2306283, rs2291075 | 38 | 0.01623 |
| ***SLCO1B1*** | *5 | no_sv | 0.5 | chr12 | 21331549:T>C | rs4149056 | 37 | 0.0158 |
| ***SLCO1B1*** | *17 | no_sv | 0.5 | chr12 | 21283322:G>A, 21329738:A>G, 21331549:T>C | rs4149015, rs2306283, rs4149056 | 36 | 0.01537 |
| ***SLCO1B1*** | *S2 | no_sv | 0 | chr12 | 21329832:G>T | rs77271279 | 19 | 0.00811 |
| ***SLCO1B1*** | *27 | no_sv | unknown | chr12 | 21329738:A>G, 21355489:C>G | rs2306283, rs59113707 | 19 | 0.00811 |
| ***SLCO1B1*** | *31 | no_sv | 0.5 | chr12 | 21329738:A>G, 21358933:G>C | rs2306283, rs59502379 | 18 | 0.00769 |
| ***SLCO1B1*** | *30 | no_sv | unknown | chr12 | 21329738:A>G, 21331891:A>G | rs2306283, rs79135870 | 11 | 0.0047 |
| ***SLCO1B1*** | *1x2 | cnv2 | 2 | chr12 |  |  | 8 | 0.00342 |
| ***SLCO1B1*** | *19 | no_sv | unknown | chr12 | 21331599:T>C, 21391976:A>C | rs4149057, rs34671512 | 6 | 0.00256 |
| ***SLCO1B1*** | *DEL | cnv0 | 0 | chr12 | . |  | 6 | 0.00256 |
| ***SLCO1B1*** | *1Bx2 | cnv2 | 2 | chr12 |  |  | 2 | 0.00085 |
| ***SLCO1B1*** | *22 | no_sv | unknown | chr12 | 21391976:A>C | rs34671512 | 2 | 0.00085 |
| ***SLCO1B1*** | *24 | no_sv | unknown | chr12 | 21329738:A>G, 21349885:A>G | rs2306283, rs11045852 | 2 | 0.00085 |
| ***SLCO1B1*** | . | . | . | chr12 |  |  | 2 | 0.00085 |
| ***SLCO1B1*** | *9 | no_sv | 0.5 | chr12 | 21358933:G>C | rs59502379 | 1 | 0.00043 |
| ***SLCO1B1*** | *S2x2 | cnv2 | 0 | chr12 |  |  | 1 | 0.00043 |
| ***SLCO2B1*** | *1 | no_sv | 1 | chr11 | ref |  | 2052 | 0.87617 |
| ***SLCO2B1*** | *S464F | no_sv | unknown | chr11 | 74907582:C>T | rs2306168 | 259 | 0.11059 |
| ***SLCO2B1*** | *S1 | no_sv | 0 | chr11 | 74873754:GCACAGAAAA>G | rs72408262 | 28 | 0.01196 |
| ***SLCO2B1*** | *1x2 | cnv2 | 2 | chr11 |  |  | 2 | 0.00085 |
| ***SLCO2B1*** | *DEL | cnv0 | 0 | chr11 | . |  | 1 | 0.00043 |
| ***TPMT*** | *1 | no_sv | 1 | chr6 | ref |  | 2215 | 0.94577 |
| ***TPMT*** | *3C | no_sv | 0 | chr6 | 18130918:T>C, 18130918:T>C | rs1142345, rs1142345 | 41 | 0.01751 |
| ***TPMT*** | *3A | no_sv | 0 | chr6 | 18130918:T>C, 18130918:T>C, 18139228:C>T | rs1142345, rs1142345, rs1800460 | 36 | 0.01537 |
| ***TPMT*** | *2 | no_sv | 0 | chr6 | 18143955:C>G | rs1800462 | 18 | 0.00769 |
| ***TPMT*** | *8 | no_sv | unknown | chr6 | 18130993:C>T | rs56161402 | 17 | 0.00726 |
| ***TPMT*** | *3B | no_sv | 0 | chr6 | 18139228:C>T | rs1800460 | 9 | 0.00384 |
| ***TPMT*** | *24 | no_sv | unknown | chr6 | 18134078:C>A | rs6921269 | 6 | 0.00256 |
| ***UGT1A1*** | *60 | no_sv | 1 | chr2 | 234665659:T>G | rs4124874 | 825 | 0.35226 |
| ***UGT1A1*** | *28 | no_sv | 0.5 | chr2 | 234668879:CAT>CATAT, 234668879:CAT>CATAT, 234668879:CAT>CATAT | rs34983651, rs34983651, rs34983651 | 771 | 0.32921 |
| ***UGT1A1*** | *1 | no_sv | 1 | chr2 | ref |  | 687 | 0.29334 |
| ***UGT1A1*** | *37 | no_sv | 0.5 | chr2 | 234668879:CAT>CATATAT, 234668879:CAT>CATATAT, 234668879:CAT>CATATAT | rs34983651, rs34983651, rs34983651 | 29 | 0.01238 |
| ***UGT1A1*** | *6 | no_sv | 0.5 | chr2 | 234669144:G>A | rs4148323 | 15 | 0.0064 |
| ***UGT1A1*** | *36 | no_sv | 1.5 | chr2 | 234668879:CAT>C, 234668879:CAT>C, 234668879:CAT>C | rs34983651, rs34983651, rs34983651 | 12 | 0.00512 |
| ***UGT1A1*** | *28x2 | cnv2 | 1 | chr2 |  |  | 2 | 0.00085 |
| ***UGT1A1*** | *7 | no_sv | 0.5 | chr2 | 234681059:T>G | rs34993780 | 1 | 0.00043 |
| ***UGT2B7*** | *1 | no_sv | 1 | chr4 | ref |  | 1328 | 0.56704 |
| ***UGT2B7*** | *2 | no_sv | unknown | chr4 | 69964338:C>T | rs7439366 | 957 | 0.40863 |
| ***UGT2B7*** | *1x2 | cnv2 | 2 | chr4 |  |  | 28 | 0.01196 |
| ***UGT2B7*** | *2x2 | cnv2 | unknown | chr4 |  |  | 14 | 0.00598 |
| ***UGT2B7*** | *3 | no_sv | unknown | chr4 | 69962449:G>T | rs12233719 | 13 | 0.00555 |
| ***UGT2B7*** | *DEL | cnv0 | 0 | chr4 | . |  | 2 | 0.00085 |
| ***UGT2B15*** | *4 | no_sv | unknown | chr4 | 69512847:T>G | rs4148269 | 642 | 0.27412 |
| ***UGT2B15*** | *1 | no_sv | 1 | chr4 | ref |  | 614 | 0.26217 |
| ***UGT2B15*** | *2 | no_sv | unknown | chr4 | 69536084:C>A | rs1902023 | 585 | 0.24979 |
| ***UGT2B15*** | *5 | no_sv | unknown | chr4 | 69512847:T>G, 69536084:C>A | rs4148269, rs1902023 | 472 | 0.20154 |
| ***UGT2B15*** | *S1 | del_i3e6 | 0 | chr4 | . |  | 7 | 0.00299 |
| ***UGT2B15*** | *2x2 | cnv2 | unknown | chr4 |  |  | 6 | 0.00256 |
| ***UGT2B15*** | *DEL | cnv0 | 0 | chr4 | . |  | 6 | 0.00256 |
| ***UGT2B15*** | *5x2 | cnv2 | unknown | chr4 |  |  | 4 | 0.00171 |
| ***UGT2B15*** | *4x2 | cnv2 | unknown | chr4 |  |  | 2 | 0.00085 |
| ***UGT2B15*** | . | . | . | chr4 |  |  | 2 | 0.00085 |
| ***UGT2B15*** | *1x2 | cnv2 | 2 | chr4 |  |  | 1 | 0.00043 |
| ***UGT2B15*** | *6 | no_sv | unknown | chr4 | 69520851:G>A | rs148583958 | 1 | 0.00043 |
| ***UGT2B17*** | *1 | no_sv | 1 | chr4 | ref |  | 1538 | 0.6567 |
| ***UGT2B17*** | *2 | cnv0 | 0 | chr4 | . |  | 804 | 0.3433 |
| ***VKORC1*** | *3 | no_sv | unknown | chr16 | 31102321:C>T | rs7294 | 815 | 0.34799 |
| ***VKORC1*** | *2 | no_sv | unknown | chr16 | 31103796:G>A, 31104878:G>A, 31107689:C>T | rs2359612, rs9934438, rs9923231 | 775 | 0.33091 |
| ***VKORC1*** | *4 | no_sv | unknown | chr16 | 31105353:G>A | rs17708472 | 381 | 0.16268 |
| ***VKORC1*** | *1 | no_sv | 1 | chr16 | ref |  | 311 | 0.13279 |
| ***VKORC1*** | *DEL | cnv0 | 0 | chr16 | . |  | 55 | 0.02348 |
| ***VKORC1*** | *V66M | no_sv | unknown | chr16 | 31104720:C>T | rs72547529 | 3 | 0.00128 |
| ***VKORC1*** | *2x2 | cnv2 | unknown | chr16 |  |  | 2 | 0.00085 |

^¥^ Activity score attributed by Stargazer

^£^ Changes described according to GRCh37/hg19

**Supplemeyary Table 2.** Diplotypes frequencies identified by Stargazer 1.0.7.

| **Gene** | **Star Allele**  **Diplotype** | **Presence of SVs** | **Diplotype**  **Frequency** | **Diplotype**  **Count** | **PharmGKB Phenotype**  **Summary** | **EHR Priority**  **Result Notation** |
| --- | --- | --- | --- | --- | --- | --- |
| ***CYP1A1*** | *1, *1 | no_sv,no_sv | 53.2 | 623 | NA | NA |
| ***CYP1A1*** | *1, *2A | no_sv,no_sv | 14.86 | 174 | NA | NA |
| ***CYP1A1*** | *1, *2B | no_sv,no_sv | 13.58 | 159 | NA | NA |
| ***CYP1A1*** | *1, *4 | no_sv,no_sv | 8.45 | 99 | NA | NA |
| ***CYP1A1*** | *2B, *2A | no_sv,no_sv | 2.39 | 28 | NA | NA |
| ***CYP1A1*** | *2A, *2A | no_sv,no_sv | 1.37 | 16 | NA | NA |
| ***CYP1A1*** | *2B, *2B | no_sv,no_sv | 1.11 | 13 | NA | NA |
| ***CYP1A1*** | *2A, *4 | no_sv,no_sv | 0.94 | 11 | NA | NA |
| ***CYP1A1*** | *2C, *2A | no_sv,no_sv | 0.68 | 8 | NA | NA |
| ***CYP1A1*** | *2B, *4 | no_sv,no_sv | 0.6 | 7 | NA | NA |
| ***CYP1A1*** | *1, *DEL | no_sv,cnv0 | 0.43 | 5 | NA | NA |
| ***CYP1A1*** | *2A, *2C | no_sv,no_sv | 0.34 | 4 | NA | NA |
| ***CYP1A1*** | *4, *4 | no_sv,no_sv | 0.26 | 3 | NA | NA |
| ***CYP1A1*** | *1, *13 | no_sv,no_sv | 0.26 | 3 | NA | NA |
| ***CYP1A1*** | *2B, *13 | no_sv,no_sv | 0.26 | 3 | NA | NA |
| ***CYP1A1*** | *1, *1x2 | no_sv,cnv2 | 0.26 | 3 | NA | NA |
| ***CYP1A1*** | *2B, *DEL | no_sv,cnv0 | 0.26 | 3 | NA | NA |
| ***CYP1A1*** | *1, *5 | no_sv,no_sv | 0.17 | 2 | NA | NA |
| ***CYP1A1*** | *2A, *13 | no_sv,no_sv | 0.17 | 2 | NA | NA |
| ***CYP1A1*** | *2A, *DEL | no_sv,cnv0 | 0.17 | 2 | NA | NA |
| ***CYP1A1*** | *2A, *2B | no_sv,no_sv | 0.09 | 1 | NA | NA |
| ***CYP1A1*** | *13, *13 | no_sv,no_sv | 0.09 | 1 | NA | NA |
| ***CYP1A1*** | ., . | no_sv,cnv2 | 0.09 | 1 | NA | NA |
| ***CYP1A2*** | *1, *1F | no_sv,no_sv | 34.67 | 406 | NA | NA |
| ***CYP1A2*** | *1F, *1F | no_sv,no_sv | 29.38 | 344 | NA | NA |
| ***CYP1A2*** | *1L, *1F | no_sv,no_sv | 12.04 | 141 | NA | NA |
| ***CYP1A2*** | *1, *1 | no_sv,no_sv | 10.76 | 126 | NA | NA |
| ***CYP1A2*** | *1, *1L | no_sv,no_sv | 8.11 | 95 | NA | NA |
| ***CYP1A2*** | *1L, *1L | no_sv,no_sv | 2.99 | 35 | NA | NA |
| ***CYP1A2*** | *1F, *1L | no_sv,no_sv | 0.51 | 6 | NA | NA |
| ***CYP1A2*** | *1F, *DEL | no_sv,cnv0 | 0.51 | 6 | NA | NA |
| ***CYP1A2*** | *1L, *DEL | no_sv,cnv0 | 0.51 | 6 | NA | NA |
| ***CYP1A2*** | *1C, *1F | no_sv,no_sv | 0.17 | 2 | NA | NA |
| ***CYP1A2*** | *1, *DEL | no_sv,cnv0 | 0.09 | 1 | NA | NA |
| ***CYP1A2*** | *1F, *1Fx2 | no_sv,cnv2 | 0.09 | 1 | NA | NA |
| ***CYP1A2*** | *1F, *1x2 | no_sv,cnv2 | 0.09 | 1 | NA | NA |
| ***CYP1A2*** | *1L, *1C | no_sv,no_sv | 0.09 | 1 | NA | NA |
| ***CYP26A1*** | *1, *1 | no_sv,no_sv | 97.69 | 1144 | NA | NA |
| ***CYP26A1*** | *1, *2 | no_sv,no_sv | 2.31 | 27 | NA | NA |
| ***CYP2A13*** | *1, *1 | no_sv,no_sv | 85.4 | 1000 | NA | NA |
| ***CYP2A13*** | *1, *2 | no_sv,no_sv | 8.11 | 95 | NA | NA |
| ***CYP2A13*** | *1, *8 | no_sv,no_sv | 2.73 | 32 | NA | NA |
| ***CYP2A13*** | *1, *7 | no_sv,no_sv | 1.79 | 21 | NA | NA |
| ***CYP2A13*** | *1, *DEL | no_sv,cnv0 | 0.68 | 8 | NA | NA |
| ***CYP2A13*** | *1, *1x2 | no_sv,cnv2 | 0.26 | 3 | NA | NA |
| ***CYP2A13*** | *2, *2 | no_sv,no_sv | 0.26 | 3 | NA | NA |
| ***CYP2A13*** | *2, *DEL | no_sv,cnv0 | 0.26 | 3 | NA | NA |
| ***CYP2A13*** | *8, *8 | no_sv,no_sv | 0.26 | 3 | NA | NA |
| ***CYP2A13*** | *2, *8 | no_sv,no_sv | 0.17 | 2 | NA | NA |
| ***CYP2A13*** | *1x2, *2 | no_sv,cnv2 | 0.09 | 1 | NA | NA |
| ***CYP2A6*** | *1, *1 | no_sv,no_sv | 61.57 | 721 | NA | NA |
| ***CYP2A6*** | *1, *9 | no_sv,no_sv | 12.13 | 142 | NA | NA |
| ***CYP2A6*** | *1, *12 | no_sv,gc_e1e2 | 3.76 | 44 | NA | NA |
| ***CYP2A6*** | *1, *17 | no_sv,no_sv | 3.07 | 36 | NA | NA |
| ***CYP2A6*** | *1, *2 | no_sv,no_sv | 3.07 | 36 | NA | NA |
| ***CYP2A6*** | *1, *14 | no_sv,no_sv | 2.56 | 30 | NA | NA |
| ***CYP2A6*** | *1, *18 | no_sv,no_sv | 2.39 | 28 | NA | NA |
| ***CYP2A6*** | *1, *4 | no_sv,cnv0 | 1.71 | 20 | NA | NA |
| ***CYP2A6*** | *1, *21 | no_sv,no_sv | 1.2 | 14 | NA | NA |
| ***CYP2A6*** | *1, *31 | no_sv,no_sv | 0.6 | 7 | NA | NA |
| ***CYP2A6*** | *9, *9 | no_sv,no_sv | 0.6 | 7 | NA | NA |
| ***CYP2A6*** | *1, *1+*S2 | no_sv,gc_e9 | 0.6 | 7 | NA | NA |
| ***CYP2A6*** | *1, *1x2 | no_sv,cnv2 | 0.43 | 5 | NA | NA |
| ***CYP2A6*** | *1x2, *4 | cnv0,cnv2 | 0.34 | 4 | NA | NA |
| ***CYP2A6*** | *9, *14 | no_sv,no_sv | 0.26 | 3 | NA | NA |
| ***CYP2A6*** | *1, *20 | no_sv,no_sv | 0.26 | 3 | NA | NA |
| ***CYP2A6*** | *1, *23 | no_sv,no_sv | 0.26 | 3 | NA | NA |
| ***CYP2A6*** | *17, *17 | no_sv,no_sv | 0.26 | 3 | NA | NA |
| ***CYP2A6*** | *1+*S2, *18 | no_sv,gc_e9 | 0.26 | 3 | NA | NA |
| ***CYP2A6*** | *2, *17 | no_sv,no_sv | 0.26 | 3 | NA | NA |
| ***CYP2A6*** | *4, *4 | cnv0,cnv0 | 0.26 | 3 | NA | NA |
| ***CYP2A6*** | *12, *12 | gc_e1e2,gc_e1e2 | 0.26 | 3 | NA | NA |
| ***CYP2A6*** | *9, *12 | no_sv,gc_e1e2 | 0.26 | 3 | NA | NA |
| ***CYP2A6*** | *1, *1+*S6 | no_sv,dup7b | 0.17 | 2 | NA | NA |
| ***CYP2A6*** | *17, *18 | no_sv,no_sv | 0.17 | 2 | NA | NA |
| ***CYP2A6*** | *12, *31 | no_sv,gc_e1e2 | 0.17 | 2 | NA | NA |
| ***CYP2A6*** | *9, *17 | no_sv,no_sv | 0.17 | 2 | NA | NA |
| ***CYP2A6*** | *9, *18 | no_sv,no_sv | 0.17 | 2 | NA | NA |
| ***CYP2A6*** | *1+*S2, *2 | no_sv,gc_e9 | 0.17 | 2 | NA | NA |
| ***CYP2A6*** | *1+*S2, *4 | cnv0,gc_e9 | 0.17 | 2 | NA | NA |
| ***CYP2A6*** | *1, *25 | no_sv,no_sv | 0.17 | 2 | NA | NA |
| ***CYP2A6*** | *4, *25 | no_sv,cnv0 | 0.09 | 1 | NA | NA |
| ***CYP2A6*** | *9, *25x2 | no_sv,cnv2 | 0.09 | 1 | NA | NA |
| ***CYP2A6*** | *4, *17 | no_sv,cnv0 | 0.09 | 1 | NA | NA |
| ***CYP2A6*** | *4, *12 | cnv0,gc_e1e2 | 0.09 | 1 | NA | NA |
| ***CYP2A6*** | *20, *21 | no_sv,no_sv | 0.09 | 1 | NA | NA |
| ***CYP2A6*** | *2, *9 | no_sv,no_sv | 0.09 | 1 | NA | NA |
| ***CYP2A6*** | *2, *25 | no_sv,no_sv | 0.09 | 1 | NA | NA |
| ***CYP2A6*** | *2, *21 | no_sv,no_sv | 0.09 | 1 | NA | NA |
| ***CYP2A6*** | *9, *31 | no_sv,no_sv | 0.09 | 1 | NA | NA |
| ***CYP2A6*** | *2, *2 | no_sv,no_sv | 0.09 | 1 | NA | NA |
| ***CYP2A6*** | *1+*S2, *17 | no_sv,gc_e9 | 0.09 | 1 | NA | NA |
| ***CYP2A6*** | *2, *14 | no_sv,no_sv | 0.09 | 1 | NA | NA |
| ***CYP2A6*** | *1x2, *25x2 | cnv2,cnv2 | 0.09 | 1 | NA | NA |
| ***CYP2A6*** | *1x2, *25 | no_sv,cnv2 | 0.09 | 1 | NA | NA |
| ***CYP2A6*** | *18, *18 | no_sv,no_sv | 0.09 | 1 | NA | NA |
| ***CYP2A6*** | *17, *25 | no_sv,no_sv | 0.09 | 1 | NA | NA |
| ***CYP2A6*** | *17, *21 | no_sv,no_sv | 0.09 | 1 | NA | NA |
| ***CYP2A6*** | *17, *20 | no_sv,no_sv | 0.09 | 1 | NA | NA |
| ***CYP2A6*** | *14, *21 | no_sv,no_sv | 0.09 | 1 | NA | NA |
| ***CYP2A6*** | *14, *17 | no_sv,no_sv | 0.09 | 1 | NA | NA |
| ***CYP2A6*** | *14, *14 | no_sv,no_sv | 0.09 | 1 | NA | NA |
| ***CYP2A6*** | *12, *21 | no_sv,gc_e1e2 | 0.09 | 1 | NA | NA |
| ***CYP2A6*** | *12, *17 | no_sv,gc_e1e2 | 0.09 | 1 | NA | NA |
| ***CYP2A6*** | *12, *14 | no_sv,gc_e1e2 | 0.09 | 1 | NA | NA |
| ***CYP2A6*** | *1, *26 | no_sv,no_sv | 0.09 | 1 | NA | NA |
| ***CYP2A6*** | *1+*S2, *9 | no_sv,gc_e9 | 0.09 | 1 | NA | NA |
| ***CYP2A6*** | *14, *18 | no_sv,no_sv | 0.09 | 1 | NA | NA |
| ***CYP2B6*** | *1, *6 | no_sv,no_sv | 30.23 | 354 | Intermediate Metabolizer | Abnormal/Priority/High Risk |
| ***CYP2B6*** | *1, *1 | no_sv,no_sv | 27.16 | 318 | Normal Metabolizer | Normal/Routine/Low Risk |
| ***CYP2B6*** | *6, *6 | no_sv,no_sv | 7.94 | 93 | Poor Metabolizer | Abnormal/Priority/High Risk |
| ***CYP2B6*** | *1, *2 | no_sv,no_sv | 7 | 82 | Normal Metabolizer | Normal/Routine/Low Risk |
| ***CYP2B6*** | *1, *5 | no_sv,no_sv | 6.32 | 74 | Normal Metabolizer | Normal/Routine/Low Risk |
| ***CYP2B6*** | *5, *6 | no_sv,no_sv | 5.04 | 59 | Intermediate Metabolizer | Abnormal/Priority/High Risk |
| ***CYP2B6*** | *2, *6 | no_sv,no_sv | 2.56 | 30 | Intermediate Metabolizer | Abnormal/Priority/High Risk |
| ***CYP2B6*** | *1, *4 | no_sv,no_sv | 1.79 | 21 | Rapid Metabolizer | Normal/Routine/Low Risk |
| ***CYP2B6*** | *1, *18 | no_sv,no_sv | 1.45 | 17 | Intermediate Metabolizer | Abnormal/Priority/High Risk |
| ***CYP2B6*** | *1, *22 | no_sv,no_sv | 1.45 | 17 | Rapid Metabolizer | Normal/Routine/Low Risk |
| ***CYP2B6*** | *6, *22 | no_sv,no_sv | 0.94 | 11 | Intermediate Metabolizer | Abnormal/Priority/High Risk |
| ***CYP2B6*** | *1, *10 | no_sv,no_sv | 0.85 | 10 | Indeterminate | None |
| ***CYP2B6*** | *6, *18 | no_sv,no_sv | 0.68 | 8 | Poor Metabolizer | Abnormal/Priority/High Risk |
| ***CYP2B6*** | *4, *6 | no_sv,no_sv | 0.68 | 8 | Intermediate Metabolizer | Abnormal/Priority/High Risk |
| ***CYP2B6*** | *6, *10 | no_sv,no_sv | 0.6 | 7 | Indeterminate | None |
| ***CYP2B6*** | *5, *22 | no_sv,no_sv | 0.51 | 6 | Rapid Metabolizer | Normal/Routine/Low Risk |
| ***CYP2B6*** | *2, *5 | no_sv,no_sv | 0.51 | 6 | Normal Metabolizer | Normal/Routine/Low Risk |
| ***CYP2B6*** | *4, *9 | no_sv,no_sv | 0.43 | 5 | Intermediate Metabolizer | Abnormal/Priority/High Risk |
| ***CYP2B6*** | *6, *15 | no_sv,no_sv | 0.34 | 4 | Indeterminate | None |
| ***CYP2B6*** | *5, *5 | no_sv,no_sv | 0.34 | 4 | Normal Metabolizer | Normal/Routine/Low Risk |
| ***CYP2B6*** | *5, *18 | no_sv,no_sv | 0.26 | 3 | Intermediate Metabolizer | Abnormal/Priority/High Risk |
| ***CYP2B6*** | *1, *15 | no_sv,no_sv | 0.17 | 2 | Indeterminate | None |
| ***CYP2B6*** | *10, *18 | no_sv,no_sv | 0.17 | 2 | Indeterminate | None |
| ***CYP2B6*** | *1, *9 | no_sv,no_sv | 0.17 | 2 | Intermediate Metabolizer | Abnormal/Priority/High Risk |
| ***CYP2B6*** | *2, *2 | no_sv,no_sv | 0.17 | 2 | Normal Metabolizer | Normal/Routine/Low Risk |
| ***CYP2B6*** | *2, *18 | no_sv,no_sv | 0.17 | 2 | Intermediate Metabolizer | Abnormal/Priority/High Risk |
| ***CYP2B6*** | *1, *11 | no_sv,no_sv | 0.17 | 2 | Indeterminate | None |
| ***CYP2B6*** | *1, *13 | no_sv,no_sv | 0.09 | 1 | Intermediate Metabolizer | Abnormal/Priority/High Risk |
| ***CYP2B6*** | *6, *29 | no_sv,gc_i4e9 | 0.09 | 1 | Poor Metabolizer | Abnormal/Priority/High Risk |
| ***CYP2B6*** | *6, *13 | no_sv,no_sv | 0.09 | 1 | Poor Metabolizer | Abnormal/Priority/High Risk |
| ***CYP2B6*** | *6, *11 | no_sv,no_sv | 0.09 | 1 | Indeterminate | None |
| ***CYP2B6*** | *1, *36 | no_sv,no_sv | 0.09 | 1 | Intermediate Metabolizer | Abnormal/Priority/High Risk |
| ***CYP2B6*** | *5, *9 | no_sv,no_sv | 0.09 | 1 | Intermediate Metabolizer | Abnormal/Priority/High Risk |
| ***CYP2B6*** | *1, *8 | no_sv,no_sv | 0.09 | 1 | Intermediate Metabolizer | Abnormal/Priority/High Risk |
| ***CYP2B6*** | *18, *18 | no_sv,no_sv | 0.09 | 1 | Poor Metabolizer | Abnormal/Priority/High Risk |
| ***CYP2B6*** | *5, *13 | no_sv,no_sv | 0.09 | 1 | Intermediate Metabolizer | Abnormal/Priority/High Risk |
| ***CYP2B6*** | *5, *15 | no_sv,no_sv | 0.09 | 1 | Indeterminate | None |
| ***CYP2B6*** | *18, *22 | no_sv,no_sv | 0.09 | 1 | Intermediate Metabolizer | Abnormal/Priority/High Risk |
| ***CYP2B6*** | *5, *10 | no_sv,no_sv | 0.09 | 1 | Indeterminate | None |
| ***CYP2B6*** | *10, *15 | no_sv,no_sv | 0.09 | 1 | Indeterminate | None |
| ***CYP2B6*** | *4, *5 | no_sv,no_sv | 0.09 | 1 | Rapid Metabolizer | Normal/Routine/Low Risk |
| ***CYP2B6*** | *4, *13 | no_sv,no_sv | 0.09 | 1 | Intermediate Metabolizer | Abnormal/Priority/High Risk |
| ***CYP2B6*** | *4, *10 | no_sv,no_sv | 0.09 | 1 | Indeterminate | None |
| ***CYP2B6*** | *22, *22 | no_sv,no_sv | 0.09 | 1 | Ultrarapid Metabolizer | Normal/Routine/Low Risk |
| ***CYP2B6*** | *10, *22x2 | no_sv,cnv2 | 0.09 | 1 | Indeterminate | None |
| ***CYP2B6*** | *2, *4 | no_sv,no_sv | 0.09 | 1 | Rapid Metabolizer | Normal/Routine/Low Risk |
| ***CYP2B6*** | *2, *22 | no_sv,no_sv | 0.09 | 1 | Rapid Metabolizer | Normal/Routine/Low Risk |
| ***CYP2B6*** | *18, *29 | no_sv,gc_i4e9 | 0.09 | 1 | Poor Metabolizer | Abnormal/Priority/High Risk |
| ***CYP2B6*** | *6, *9 | no_sv,no_sv | 0.09 | 1 | Poor Metabolizer | Abnormal/Priority/High Risk |
| ***CYP2C19*** | *1, *1 | no_sv,no_sv | 38.94 | 456 | Normal Metabolizer | Normal/Routine/Low Risk |
| ***CYP2C19*** | *1, *17 | no_sv,no_sv | 24.34 | 285 | Rapid Metabolizer | Abnormal/Priority/High Risk |
| ***CYP2C19*** | *1, *2 | no_sv,no_sv | 19.56 | 229 | Intermediate Metabolizer | Abnormal/Priority/High Risk |
| ***CYP2C19*** | *2, *17 | no_sv,no_sv | 6.4 | 75 | Intermediate Metabolizer | Abnormal/Priority/High Risk |
| ***CYP2C19*** | *17, *17 | no_sv,no_sv | 3.84 | 45 | Ultrarapid Metabolizer | Abnormal/Priority/High Risk |
| ***CYP2C19*** | *2, *2 | no_sv,no_sv | 1.54 | 18 | Poor Metabolizer | Abnormal/Priority/High Risk |
| ***CYP2C19*** | *1, *35 | no_sv,no_sv | 1.02 | 12 | Intermediate Metabolizer | Abnormal/Priority/High Risk |
| ***CYP2C19*** | *1, *9 | no_sv,no_sv | 0.85 | 10 | Likely Intermediate Metabolizer | Abnormal/Priority/High Risk |
| ***CYP2C19*** | *1, *8 | no_sv,no_sv | 0.51 | 6 | Intermediate Metabolizer | Abnormal/Priority/High Risk |
| ***CYP2C19*** | *1, *4 | no_sv,no_sv | 0.43 | 5 | Intermediate Metabolizer | Abnormal/Priority/High Risk |
| ***CYP2C19*** | *1, *15 | no_sv,no_sv | 0.34 | 4 | Normal Metabolizer | Normal/Routine/Low Risk |
| ***CYP2C19*** | *1, *3 | no_sv,no_sv | 0.34 | 4 | Intermediate Metabolizer | Abnormal/Priority/High Risk |
| ***CYP2C19*** | *2, *35 | no_sv,no_sv | 0.26 | 3 | Poor Metabolizer | Abnormal/Priority/High Risk |
| ***CYP2C19*** | *2, *3 | no_sv,no_sv | 0.26 | 3 | Poor Metabolizer | Abnormal/Priority/High Risk |
| ***CYP2C19*** | *2, *13 | no_sv,no_sv | 0.17 | 2 | Intermediate Metabolizer | Abnormal/Priority/High Risk |
| ***CYP2C19*** | *15, *17 | no_sv,no_sv | 0.17 | 2 | Rapid Metabolizer | Abnormal/Priority/High Risk |
| ***CYP2C19*** | *1, *12 | no_sv,no_sv | 0.17 | 2 | Indeterminate | none |
| ***CYP2C19*** | *9, *17 | no_sv,no_sv | 0.17 | 2 | Likely Intermediate Metabolizer | Abnormal/Priority/High Risk |
| ***CYP2C19*** | *17, *35 | no_sv,no_sv | 0.09 | 1 | Intermediate Metabolizer | Abnormal/Priority/High Risk |
| ***CYP2C19*** | *2, *15 | no_sv,no_sv | 0.09 | 1 | Intermediate Metabolizer | Abnormal/Priority/High Risk |
| ***CYP2C19*** | *10, *35 | no_sv,no_sv | 0.09 | 1 | Likely Poor Metabolizer | Abnormal/Priority/High Risk |
| ***CYP2C19*** | *1, *5 | no_sv,no_sv | 0.09 | 1 | Intermediate Metabolizer | Abnormal/Priority/High Risk |
| ***CYP2C19*** | *1, *13 | no_sv,no_sv | 0.09 | 1 | Normal Metabolizer | Normal/Routine/Low Risk |
| ***CYP2C19*** | *2, *9 | no_sv,no_sv | 0.09 | 1 | Likely Poor Metabolizer | Abnormal/Priority/High Risk |
| ***CYP2C19*** | *3, *17 | no_sv,no_sv | 0.09 | 1 | Intermediate Metabolizer | Abnormal/Priority/High Risk |
| ***CYP2C19*** | *4, *35 | no_sv,no_sv | 0.09 | 1 | Poor Metabolizer | Abnormal/Priority/High Risk |
| ***CYP2C8*** | *1, *1 | no_sv,no_sv | 65.84 | 771 | NA | NA |
| ***CYP2C8*** | *1, *3 | no_sv,no_sv | 16.74 | 196 | NA | NA |
| ***CYP2C8*** | *1, *4 | no_sv,no_sv | 6.92 | 81 | NA | NA |
| ***CYP2C8*** | *1, *2 | no_sv,no_sv | 5.98 | 70 | NA | NA |
| ***CYP2C8*** | *3, *3 | no_sv,no_sv | 1.62 | 19 | NA | NA |
| ***CYP2C8*** | *1, *1x2 | no_sv,cnv2 | 1.02 | 12 | NA | NA |
| ***CYP2C8*** | *3, *4 | no_sv,no_sv | 0.77 | 9 | NA | NA |
| ***CYP2C8*** | *2, *2 | no_sv,no_sv | 0.43 | 5 | NA | NA |
| ***CYP2C8*** | *2, *3 | no_sv,no_sv | 0.17 | 2 | NA | NA |
| ***CYP2C8*** | *1, *2x2 | no_sv,cnv2 | 0.09 | 1 | NA | NA |
| ***CYP2C8*** | *1, *3x2 | no_sv,cnv2 | 0.09 | 1 | NA | NA |
| ***CYP2C8*** | *1, *7 | no_sv,no_sv | 0.09 | 1 | NA | NA |
| ***CYP2C8*** | *1, *DEL | no_sv,cnv0 | 0.09 | 1 | NA | NA |
| ***CYP2C8*** | *1x2, *3 | no_sv,cnv2 | 0.09 | 1 | NA | NA |
| ***CYP2C8*** | *2, *4 | no_sv,no_sv | 0.09 | 1 | NA | NA |
| ***CYP2C9*** | *1, *1 | no_sv,no_sv | 62.51 | 732 | Normal Metabolizer | Normal/Routine/ Low Risk |
| ***CYP2C9*** | *1, *2 | no_sv,no_sv | 17.51 | 205 | Intermediate Metabolizer | Abnormal/Priority/High Risk |
| ***CYP2C9*** | *1, *3 | no_sv,no_sv | 8.37 | 98 | Intermediate Metabolizer | Abnormal/Priority/High Risk |
| ***CYP2C9*** | *1, *9 | no_sv,no_sv | 3.16 | 37 | Normal Metabolizer | Normal/Routine/ Low Risk |
| ***CYP2C9*** | *2, *2 | no_sv,no_sv | 1.62 | 19 | Intermediate Metabolizer | Abnormal/Priority/High Risk |
| ***CYP2C9*** | *1, *8 | no_sv,no_sv | 1.54 | 18 | Intermediate Metabolizer | Abnormal/Priority/High Risk |
| ***CYP2C9*** | *1, *11 | no_sv,no_sv | 1.02 | 12 | Intermediate Metabolizer | Abnormal/Priority/High Risk |
| ***CYP2C9*** | *2, *3 | no_sv,no_sv | 0.94 | 11 | Poor Metabolizer | Abnormal/Priority/High Risk |
| ***CYP2C9*** | *1, *5 | no_sv,no_sv | 0.6 | 7 | Intermediate Metabolizer | Abnormal/Priority/High Risk |
| ***CYP2C9*** | *1, *1x2 | no_sv,cnv2 | 0.51 | 6 | Indeterminate | none |
| ***CYP2C9*** | *3, *3 | no_sv,no_sv | 0.43 | 5 | Poor Metabolizer | Abnormal/Priority/High Risk |
| ***CYP2C9*** | *2, *8 | no_sv,no_sv | 0.34 | 4 | Intermediate Metabolizer | Abnormal/Priority/High Risk |
| ***CYP2C9*** | *2, *9 | no_sv,no_sv | 0.34 | 4 | Intermediate Metabolizer | Abnormal/Priority/High Risk |
| ***CYP2C9*** | *3, *9 | no_sv,no_sv | 0.09 | 1 | Intermediate Metabolizer | Abnormal/Priority/High Risk |
| ***CYP2C9*** | *5, *11 | no_sv,no_sv | 0.09 | 1 | Intermediate Metabolizer | Abnormal/Priority/High Risk |
| ***CYP2C9*** | *5, *36 | no_sv,no_sv | 0.09 | 1 | Indeterminate | none |
| ***CYP2C9*** | *8, *9 | no_sv,no_sv | 0.09 | 1 | Intermediate Metabolizer | Abnormal/Priority/High Risk |
| ***CYP2C9*** | *1x2, *3 | no_sv,cnv2 | 0.09 | 1 | Indeterminate | none |
| ***CYP2C9*** | *2, *36 | no_sv,no_sv | 0.09 | 1 | Indeterminate | none |
| ***CYP2C9*** | *2, *11 | no_sv,no_sv | 0.09 | 1 | Intermediate Metabolizer | Abnormal/Priority/High Risk |
| ***CYP2C9*** | *1, *DEL | no_sv,cnv0 | 0.09 | 1 | Indeterminate | none |
| ***CYP2C9*** | *1, *6 | no_sv,no_sv | 0.09 | 1 | Intermediate Metabolizer | Abnormal/Priority/High Risk |
| ***CYP2C9*** | *1, *3x2 | no_sv,cnv2 | 0.09 | 1 | Indeterminate | none |
| ***CYP2C9*** | *1, *1x3 | cnv2,cnv2 | 0.09 | 1 | Indeterminate | none |
| ***CYP2C9*** | *1, *12 | no_sv,no_sv | 0.09 | 1 | Intermediate Metabolizer | Abnormal/Priority/High Risk |
| ***CYP2C9*** | *9, *9 | no_sv,no_sv | 0.09 | 1 | Normal Metabolizer | Normal/Routine/ Low Risk |
| ***CYP2D6*** | *1, *1 | no_sv,no_sv | 13.66 | 160 | Normal Metabolizer | Normal/Routine/ Low Risk |
| ***CYP2D6*** | *1, *2 | no_sv,no_sv | 9.56 | 112 | Normal Metabolizer | Normal/Routine/ Low Risk |
| ***CYP2D6*** | *1, *4 | no_sv,no_sv | 6.15 | 72 | Intermediate Metabolizer | Abnormal/Priority/High Risk |
| ***CYP2D6*** | *1, *41 | no_sv,no_sv | 5.12 | 60 | Normal Metabolizer | Normal/Routine/ Low Risk |
| ***CYP2D6*** | *2, *2 | no_sv,no_sv | 4.1 | 48 | Normal Metabolizer | Normal/Routine/ Low Risk |
| ***CYP2D6*** | *1, *68+*4 | no_sv,gc_i1e9 | 3.59 | 42 | Intermediate Metabolizer | Abnormal/Priority/High Risk |
| ***CYP2D6*** | *1, *17 | no_sv,no_sv | 2.99 | 35 | Normal Metabolizer | Normal/Routine/ Low Risk |
| ***CYP2D6*** | *2, *41 | no_sv,no_sv | 2.9 | 34 | Normal Metabolizer | Normal/Routine/ Low Risk |
| ***CYP2D6*** | *1, *35 | no_sv,no_sv | 2.82 | 33 | Normal Metabolizer | Normal/Routine/ Low Risk |
| ***CYP2D6*** | *1, *5 | no_sv,cnv0 | 2.56 | 30 | Intermediate Metabolizer | Abnormal/Priority/High Risk |
| ***CYP2D6*** | *2, *4 | no_sv,no_sv | 2.56 | 30 | Intermediate Metabolizer | Abnormal/Priority/High Risk |
| ***CYP2D6*** | *2, *17 | no_sv,no_sv | 1.79 | 21 | Normal Metabolizer | Normal/Routine/ Low Risk |
| ***CYP2D6*** | *1, *9 | no_sv,no_sv | 1.45 | 17 | Normal Metabolizer | Normal/Routine/ Low Risk |
| ***CYP2D6*** | *2, *68+*4 | no_sv,gc_i1e9 | 1.45 | 17 | Intermediate Metabolizer | Abnormal/Priority/High Risk |
| ***CYP2D6*** |  | no_sv,no_sv | 1.37 | 16 | Normal Metabolizer | Normal/Routine/ Low Risk |
| ***CYP2D6*** | *2, *35 | no_sv,no_sv | 1.37 | 16 | Normal Metabolizer | Normal/Routine/ Low Risk |
| ***CYP2D6*** | *4, *41 | no_sv,no_sv | 1.37 | 16 | Intermediate Metabolizer | Abnormal/Priority/High Risk |
| ***CYP2D6*** | *1, *29 | no_sv,no_sv | 1.37 | 16 | Normal Metabolizer | Normal/Routine/ Low Risk |
| ***CYP2D6*** | *2, *29 | no_sv,no_sv | 1.28 | 15 | Normal Metabolizer | Normal/Routine/ Low Risk |
| ***CYP2D6*** | *1, *2x2 | no_sv,cnv2 | 1.02 | 12 | Ultrarapid Metabolizer | Abnormal/Priority/High Risk |
| ***CYP2D6*** | *4, *68+*4 | no_sv,gc_i1e9 | 0.94 | 11 | Poor Metabolizer | Abnormal/Priority/High Risk |
| ***CYP2D6*** | *4, *5 | no_sv,cnv0 | 0.94 | 11 | Poor Metabolizer | Abnormal/Priority/High Risk |
| ***CYP2D6*** | *2, *9 | no_sv,no_sv | 0.85 | 10 | Normal Metabolizer | Normal/Routine/ Low Risk |
| ***CYP2D6*** | *4, *10 | no_sv,no_sv | 0.85 | 10 | Intermediate Metabolizer | Abnormal/Priority/High Risk |
| ***CYP2D6*** | *4, *4 | no_sv,no_sv | 0.85 | 10 | Poor Metabolizer | Abnormal/Priority/High Risk |
| ***CYP2D6*** | *35, *41 | no_sv,no_sv | 0.77 | 9 | Normal Metabolizer | Normal/Routine/ Low Risk |
| ***CYP2D6*** | *1, *36+*10 | no_sv,gc_e9 | 0.68 | 8 | Normal Metabolizer | Normal/Routine/ Low Risk |
| ***CYP2D6*** | *4, *35 | no_sv,no_sv | 0.68 | 8 | Intermediate Metabolizer | Abnormal/Priority/High Risk |
| ***CYP2D6*** | *41, *41 | no_sv,no_sv | 0.68 | 8 | Intermediate Metabolizer | Abnormal/Priority/High Risk |
| ***CYP2D6*** | *68+*4, *68+*4 | gc_i1e9,gc_i1e9 | 0.68 | 8 | Poor Metabolizer | Abnormal/Priority/High Risk |
| ***CYP2D6*** | *2, *2x2 | no_sv,cnv2 | 0.68 | 8 | Ultrarapid Metabolizer | Abnormal/Priority/High Risk |
| ***CYP2D6*** | *1, *3 | no_sv,no_sv | 0.6 | 7 | Intermediate Metabolizer | Abnormal/Priority/High Risk |
| ***CYP2D6*** | *2, *10 | no_sv,no_sv | 0.6 | 7 | Normal Metabolizer | Normal/Routine/ Low Risk |
| ***CYP2D6*** | *1, *1x2 | no_sv,cnv2 | 0.51 | 6 | Ultrarapid Metabolizer | Abnormal/Priority/High Risk |
| ***CYP2D6*** | *4, *17 | no_sv,no_sv | 0.51 | 6 | Intermediate Metabolizer | Abnormal/Priority/High Risk |
| ***CYP2D6*** | *1, *28 | no_sv,no_sv | 0.51 | 6 | Indeterminate | none |
| ***CYP2D6*** | *41, *68+*4 | no_sv,gc_i1e9 | 0.51 | 6 | Intermediate Metabolizer | Abnormal/Priority/High Risk |
| ***CYP2D6*** | *5, *17 | no_sv,cnv0 | 0.43 | 5 | Intermediate Metabolizer | Abnormal/Priority/High Risk |
| ***CYP2D6*** | *1, *20 | no_sv,no_sv | 0.43 | 5 | Intermediate Metabolizer | Abnormal/Priority/High Risk |
| ***CYP2D6*** | *5, *41 | no_sv,cnv0 | 0.43 | 5 | Intermediate Metabolizer | Abnormal/Priority/High Risk |
| ***CYP2D6*** | *17, *17 | no_sv,no_sv | 0.43 | 5 | Intermediate Metabolizer | Abnormal/Priority/High Risk |
| ***CYP2D6*** | *17, *41 | no_sv,no_sv | 0.43 | 5 | Intermediate Metabolizer | Abnormal/Priority/High Risk |
| ***CYP2D6*** | *1, *4x2 | no_sv,cnv2 | 0.43 | 5 | Intermediate Metabolizer | Abnormal/Priority/High Risk |
| ***CYP2D6*** | *2, *5 | no_sv,cnv0 | 0.34 | 4 | Intermediate Metabolizer | Abnormal/Priority/High Risk |
| ***CYP2D6*** | *1, *6 | no_sv,no_sv | 0.34 | 4 | Intermediate Metabolizer | Abnormal/Priority/High Risk |
| ***CYP2D6*** | *1, *43 | no_sv,no_sv | 0.34 | 4 | Indeterminate | none |
| ***CYP2D6*** | *4, *29 | no_sv,no_sv | 0.34 | 4 | Intermediate Metabolizer | Abnormal/Priority/High Risk |
| ***CYP2D6*** | *4, *9 | no_sv,no_sv | 0.34 | 4 | Intermediate Metabolizer | Abnormal/Priority/High Risk |
| ***CYP2D6*** | *2x2, *41 | no_sv,cnv2 | 0.34 | 4 | Ultrarapid Metabolizer | Abnormal/Priority/High Risk |
| ***CYP2D6*** | *2x2, *4 | no_sv,cnv2 | 0.34 | 4 | Normal Metabolizer | Normal/Routine/ Low Risk |
| ***CYP2D6*** | *4x2, *41 | no_sv,cnv2 | 0.26 | 3 | Intermediate Metabolizer | Abnormal/Priority/High Risk |
| ***CYP2D6*** | *S1+*1, *4 | no_sv,gc_i1e9 | 0.26 | 3 | Intermediate Metabolizer | Abnormal/Priority/High Risk |
| ***CYP2D6*** | *2, *2x3 | no_sv,cnv3 | 0.26 | 3 | Ultrarapid Metabolizer | Abnormal/Priority/High Risk |
| ***CYP2D6*** | *4, *28 | no_sv,no_sv | 0.26 | 3 | Indeterminate | none |
| ***CYP2D6*** | *10, *36+*10 | no_sv,gc_e9 | 0.26 | 3 | Intermediate Metabolizer | Abnormal/Priority/High Risk |
| ***CYP2D6*** | *2x2, *17 | no_sv,cnv2 | 0.26 | 3 | Ultrarapid Metabolizer | Abnormal/Priority/High Risk |
| ***CYP2D6*** | *2x2, *35 | no_sv,cnv2 | 0.26 | 3 | Ultrarapid Metabolizer | Abnormal/Priority/High Risk |
| ***CYP2D6*** | *1x2, *2 | no_sv,cnv2 | 0.26 | 3 | Ultrarapid Metabolizer | Abnormal/Priority/High Risk |
| ***CYP2D6*** | *9, *10 | no_sv,no_sv | 0.26 | 3 | Intermediate Metabolizer | Abnormal/Priority/High Risk |
| ***CYP2D6*** | *10, *41 | no_sv,no_sv | 0.26 | 3 | Intermediate Metabolizer | Abnormal/Priority/High Risk |
| ***CYP2D6*** | *17, *35 | no_sv,no_sv | 0.26 | 3 | Normal Metabolizer | Normal/Routine/ Low Risk |
| ***CYP2D6*** | *1, *2D | no_sv,no_sv | 0.17 | 2 | Normal Metabolizer | Normal/Routine/ Low Risk |
| ***CYP2D6*** | *5, *35 | no_sv,cnv0 | 0.17 | 2 | Intermediate Metabolizer | Abnormal/Priority/High Risk |
| ***CYP2D6*** | *3, *41 | no_sv,no_sv | 0.17 | 2 | Intermediate Metabolizer | Abnormal/Priority/High Risk |
| ***CYP2D6*** | *9, *35 | no_sv,no_sv | 0.17 | 2 | Normal Metabolizer | Normal/Routine/ Low Risk |
| ***CYP2D6*** | *5, *28 | no_sv,cnv0 | 0.17 | 2 | Indeterminate | none |
| ***CYP2D6*** | *9, *41 | no_sv,no_sv | 0.17 | 2 | Intermediate Metabolizer | Abnormal/Priority/High Risk |
| ***CYP2D6*** | *29, *106 | no_sv,no_sv | 0.17 | 2 | Indeterminate | none |
| ***CYP2D6*** | *29, *35 | no_sv,no_sv | 0.17 | 2 | Normal Metabolizer | Normal/Routine/ Low Risk |
| ***CYP2D6*** | *4N+*4, *68+*4 | gc_i1e9,gc_e9 | 0.17 | 2 | Poor Metabolizer | Abnormal/Priority/High Risk |
| ***CYP2D6*** | *1, *84 | no_sv,no_sv | 0.17 | 2 | Indeterminate | none |
| ***CYP2D6*** | *5, *36+*10 | cnv0,gc_e9 | 0.17 | 2 | Intermediate Metabolizer | Abnormal/Priority/High Risk |
| ***CYP2D6*** | *5, *20 | no_sv,cnv0 | 0.17 | 2 | Poor Metabolizer | Abnormal/Priority/High Risk |
| ***CYP2D6*** | *1, *33 | no_sv,no_sv | 0.17 | 2 | Normal Metabolizer | Normal/Routine/ Low Risk |
| ***CYP2D6*** | *10, *17 | no_sv,no_sv | 0.17 | 2 | Intermediate Metabolizer | Abnormal/Priority/High Risk |
| ***CYP2D6*** | *36+*10, *36+*10 | gc_e9,gc_e9 | 0.17 | 2 | Intermediate Metabolizer | Abnormal/Priority/High Risk |
| ***CYP2D6*** | *17, *29 | no_sv,no_sv | 0.17 | 2 | Intermediate Metabolizer | Abnormal/Priority/High Risk |
| ***CYP2D6*** | *10, *68+*4 | no_sv,gc_i1e9 | 0.17 | 2 | Intermediate Metabolizer | Abnormal/Priority/High Risk |
| ***CYP2D6*** | *17, *68+*4 | no_sv,gc_i1e9 | 0.17 | 2 | Intermediate Metabolizer | Abnormal/Priority/High Risk |
| ***CYP2D6*** | *1, *59 | no_sv,no_sv | 0.17 | 2 | Normal Metabolizer | Normal/Routine/ Low Risk |
| ***CYP2D6*** | *6, *41 | no_sv,no_sv | 0.17 | 2 | Intermediate Metabolizer | Abnormal/Priority/High Risk |
| ***CYP2D6*** | *1x2, *41 | no_sv,cnv2 | 0.17 | 2 | Ultrarapid Metabolizer | Abnormal/Priority/High Risk |
| ***CYP2D6*** | *6, *35 | no_sv,no_sv | 0.17 | 2 | Intermediate Metabolizer | Abnormal/Priority/High Risk |
| ***CYP2D6*** | *2, *13B | no_sv,gc_7to6_i1 | 0.17 | 2 | Intermediate Metabolizer | Abnormal/Priority/High Risk |
| ***CYP2D6*** | *3, *4 | no_sv,no_sv | 0.17 | 2 | Poor Metabolizer | Abnormal/Priority/High Risk |
| ***CYP2D6*** | *10, *35 | no_sv,no_sv | 0.17 | 2 | Normal Metabolizer | Normal/Routine/ Low Risk |
| ***CYP2D6*** | *10, *29 | no_sv,no_sv | 0.17 | 2 | Intermediate Metabolizer | Abnormal/Priority/High Risk |
| ***CYP2D6*** | *2, *3 | no_sv,no_sv | 0.17 | 2 | Intermediate Metabolizer | Abnormal/Priority/High Risk |
| ***CYP2D6*** | *5, *9 | no_sv,cnv0 | 0.17 | 2 | Intermediate Metabolizer | Abnormal/Priority/High Risk |
| ***CYP2D6*** | ., . | cnv0,gc_e9 | 0.17 | 2 | Indeterminate | none |
| ***CYP2D6*** | *4, *6 | no_sv,no_sv | 0.17 | 2 | Poor Metabolizer | Abnormal/Priority/High Risk |
| ***CYP2D6*** | *4, *46 | no_sv,no_sv | 0.09 | 1 | Intermediate Metabolizer | Abnormal/Priority/High Risk |
| ***CYP2D6*** | *4, *4x2 | no_sv,cnv2 | 0.09 | 1 | Poor Metabolizer | Abnormal/Priority/High Risk |
| ***CYP2D6*** | *S1+*1, *17 | no_sv,gc_i1e9 | 0.09 | 1 | Normal Metabolizer | Normal/Routine/ Low Risk |
| ***CYP2D6*** | *41, *106 | no_sv,no_sv | 0.09 | 1 | Indeterminate | none |
| ***CYP2D6*** | *41, *41x3 | no_sv,cnv3 | 0.09 | 1 | Normal Metabolizer | Normal/Routine/ Low Risk |
| ***CYP2D6*** | *4, *7 | no_sv,no_sv | 0.09 | 1 | Poor Metabolizer | Abnormal/Priority/High Risk |
| ***CYP2D6*** | *9, *17 | no_sv,no_sv | 0.09 | 1 | Intermediate Metabolizer | Abnormal/Priority/High Risk |
| ***CYP2D6*** | *41, *43 | no_sv,no_sv | 0.09 | 1 | Indeterminate | none |
| ***CYP2D6*** | *9, *68+*4 | no_sv,gc_i1e9 | 0.09 | 1 | Poor Metabolizer | Abnormal/Priority/High Risk |
| ***CYP2D6*** | *6, *68+*4 | no_sv,gc_i1e9 | 0.09 | 1 | Poor Metabolizer | Abnormal/Priority/High Risk |
| ***CYP2D6*** | ., . | gc_e9,gc_7to6_i4 | 0.09 | 1 | Indeterminate | none |
| ***CYP2D6*** | *6, *28 | no_sv,no_sv | 0.09 | 1 | Indeterminate | none |
| ***CYP2D6*** | *9, *33 | no_sv,no_sv | 0.09 | 1 | Normal Metabolizer | Normal/Routine/ Low Risk |
| ***CYP2D6*** | *S1+*1, *2 | no_sv,gc_i1e9 | 0.09 | 1 | Normal Metabolizer | Normal/Routine/ Low Risk |
| ***CYP2D6*** | *5, *84 | no_sv,cnv0 | 0.09 | 1 | Indeterminate | none |
| ***CYP2D6*** | *S1+*1, *20 | no_sv,gc_i1e9 | 0.09 | 1 | Normal Metabolizer | Normal/Routine/ Low Risk |
| ***CYP2D6*** | *5, *83+*2 | cnv0,gc_e9 | 0.09 | 1 | Indeterminate | none |
| ***CYP2D6*** | *9, *9 | no_sv,no_sv | 0.09 | 1 | Intermediate Metabolizer | Abnormal/Priority/High Risk |
| ***CYP2D6*** | *5, *43 | no_sv,cnv0 | 0.09 | 1 | Indeterminate | none |
| ***CYP2D6*** | *9, *29 | no_sv,no_sv | 0.09 | 1 | Intermediate Metabolizer | Abnormal/Priority/High Risk |
| ***CYP2D6*** | *5, *106 | no_sv,cnv0 | 0.09 | 1 | Indeterminate | none |
| ***CYP2D6*** | *S2+*1, *4 | no_sv,del1 | 0.09 | 1 | Intermediate Metabolizer | Abnormal/Priority/High Risk |
| ***CYP2D6*** | *5, *10 | no_sv,cnv0 | 0.09 | 1 | Intermediate Metabolizer | Abnormal/Priority/High Risk |
| ***CYP2D6*** | *46, *68+*4 | no_sv,gc_i1e9 | 0.09 | 1 | Intermediate Metabolizer | Abnormal/Priority/High Risk |
| ***CYP2D6*** | *43, *43 | no_sv,no_sv | 0.09 | 1 | Indeterminate | none |
| ***CYP2D6*** | *41, *83+*2 | no_sv,gc_e9 | 0.09 | 1 | Indeterminate | none |
| ***CYP2D6*** | *5, *68+*4 | cnv0,gc_i1e9 | 0.09 | 1 | Poor Metabolizer | Abnormal/Priority/High Risk |
| ***CYP2D6*** | *29, *68+*4 | no_sv,gc_i1e9 | 0.09 | 1 | Intermediate Metabolizer | Abnormal/Priority/High Risk |
| ***CYP2D6*** | *4, *31 | no_sv,no_sv | 0.09 | 1 | Poor Metabolizer | Abnormal/Priority/High Risk |
| ***CYP2D6*** | *10, *33 | no_sv,no_sv | 0.09 | 1 | Normal Metabolizer | Normal/Routine/ Low Risk |
| ***CYP2D6*** | *2, *36+*10 | no_sv,gc_e9 | 0.09 | 1 | Normal Metabolizer | Normal/Routine/ Low Risk |
| ***CYP2D6*** | *2, *33 | no_sv,no_sv | 0.09 | 1 | Normal Metabolizer | Normal/Routine/ Low Risk |
| ***CYP2D6*** | *2, *28 | no_sv,no_sv | 0.09 | 1 | Indeterminate | none |
| ***CYP2D6*** | *1x3, *17 | no_sv,cnv3 | 0.09 | 1 | Ultrarapid Metabolizer | Abnormal/Priority/High Risk |
| ***CYP2D6*** | *1x2, *4 | no_sv,cnv2 | 0.09 | 1 | Normal Metabolizer | Normal/Routine/ Low Risk |
| ***CYP2D6*** | *1x2, *29x2 | no_sv,cnv3 | 0.09 | 1 | Ultrarapid Metabolizer | Abnormal/Priority/High Risk |
| ***CYP2D6*** | *1x2, *20 | no_sv,cnv2 | 0.09 | 1 | Normal Metabolizer | Normal/Routine/ Low Risk |
| ***CYP2D6*** | *17, *43 | no_sv,no_sv | 0.09 | 1 | Indeterminate | none |
| ***CYP2D6*** | *17, *106 | no_sv,no_sv | 0.09 | 1 | Indeterminate | none |
| ***CYP2D6*** | *13C, *17 | no_sv,gc_e1e7 | 0.09 | 1 | Intermediate Metabolizer | Abnormal/Priority/High Risk |
| ***CYP2D6*** | *10, *10 | no_sv,no_sv | 0.09 | 1 | Intermediate Metabolizer | Abnormal/Priority/High Risk |
| ***CYP2D6*** | *2, *41x2 | no_sv,cnv2 | 0.09 | 1 | Normal Metabolizer | Normal/Routine/ Low Risk |
| ***CYP2D6*** | *1, *S2+*1 | no_sv,del1 | 0.09 | 1 | Normal Metabolizer | Normal/Routine/ Low Risk |
| ***CYP2D6*** | *1, *S1+*1 | no_sv,gc_i1e9 | 0.09 | 1 | Normal Metabolizer | Normal/Routine/ Low Risk |
| ***CYP2D6*** | *1, *4N+*4 | no_sv,gc_e9 | 0.09 | 1 | Intermediate Metabolizer | Abnormal/Priority/High Risk |
| ***CYP2D6*** | *1, *46 | no_sv,no_sv | 0.09 | 1 | Normal Metabolizer | Normal/Routine/ Low Risk |
| ***CYP2D6*** | *1, *35x2 | no_sv,cnv2 | 0.09 | 1 | Ultrarapid Metabolizer | Abnormal/Priority/High Risk |
| ***CYP2D6*** | *1, *34 | no_sv,no_sv | 0.09 | 1 | Normal Metabolizer | Normal/Routine/ Low Risk |
| ***CYP2D6*** | *1, *31 | no_sv,no_sv | 0.09 | 1 | Intermediate Metabolizer | Abnormal/Priority/High Risk |
| ***CYP2D6*** | *1, *2x3 | no_sv,cnv3 | 0.09 | 1 | Ultrarapid Metabolizer | Abnormal/Priority/High Risk |
| ***CYP2D6*** | *1, *29x2 | no_sv,cnv2 | 0.09 | 1 | Normal Metabolizer | Normal/Routine/ Low Risk |
| ***CYP2D6*** | *1, *106 | no_sv,no_sv | 0.09 | 1 | Indeterminate | none |
| ***CYP2D6*** | *2, *40 | no_sv,no_sv | 0.09 | 1 | Intermediate Metabolizer | Abnormal/Priority/High Risk |
| ***CYP2D6*** | *2, *43 | no_sv,no_sv | 0.09 | 1 | Indeterminate | none |
| ***CYP2D6*** | *4, *21x2 | no_sv,cnv2 | 0.09 | 1 | Poor Metabolizer | Abnormal/Priority/High Risk |
| ***CYP2D6*** | *2x2, *9 | no_sv,cnv2 | 0.09 | 1 | Ultrarapid Metabolizer | Abnormal/Priority/High Risk |
| ***CYP2D6*** | *4, *10x2 | no_sv,cnv2 | 0.09 | 1 | Intermediate Metabolizer | Abnormal/Priority/High Risk |
| ***CYP2D6*** | *36+*10, *41 | no_sv,gc_e9 | 0.09 | 1 | Intermediate Metabolizer | Abnormal/Priority/High Risk |
| ***CYP2D6*** | *35, *68+*4 | no_sv,gc_i1e9 | 0.09 | 1 | Intermediate Metabolizer | Abnormal/Priority/High Risk |
| ***CYP2D6*** | *33, *41x3 | no_sv,cnv3 | 0.09 | 1 | Ultrarapid Metabolizer | Abnormal/Priority/High Risk |
| ***CYP2D6*** | *33, *41 | no_sv,no_sv | 0.09 | 1 | Normal Metabolizer | Normal/Routine/ Low Risk |
| ***CYP2D6*** | *3, *68+*4 | no_sv,gc_i1e9 | 0.09 | 1 | Poor Metabolizer | Abnormal/Priority/High Risk |
| ***CYP2D6*** | *3, *6 | no_sv,no_sv | 0.09 | 1 | Poor Metabolizer | Abnormal/Priority/High Risk |
| ***CYP2D6*** | *3, *35 | no_sv,no_sv | 0.09 | 1 | Intermediate Metabolizer | Abnormal/Priority/High Risk |
| ***CYP2D6*** | *3, *33 | no_sv,no_sv | 0.09 | 1 | Intermediate Metabolizer | Abnormal/Priority/High Risk |
| ***CYP2D6*** | *3, *3 | no_sv,no_sv | 0.09 | 1 | Poor Metabolizer | Abnormal/Priority/High Risk |
| ***CYP2D6*** | *2x2, *6 | no_sv,cnv2 | 0.09 | 1 | Normal Metabolizer | Normal/Routine/ Low Risk |
| ***CYP2D6*** | *2, *4N+*4 | no_sv,gc_e9 | 0.09 | 1 | Intermediate Metabolizer | Abnormal/Priority/High Risk |
| ***CYP2D6*** | *2x2, *43 | no_sv,cnv2 | 0.09 | 1 | Indeterminate | none |
| ***CYP2D6*** | *2x2, *41x2 | no_sv,cnv3 | 0.09 | 1 | Ultrarapid Metabolizer | Abnormal/Priority/High Risk |
| ***CYP2D6*** | *2x2, *33 | no_sv,cnv2 | 0.09 | 1 | Ultrarapid Metabolizer | Abnormal/Priority/High Risk |
| ***CYP2D6*** | *2x2, *29 | no_sv,cnv2 | 0.09 | 1 | Ultrarapid Metabolizer | Abnormal/Priority/High Risk |
| ***CYP2D6*** | *29, *59 | no_sv,no_sv | 0.09 | 1 | Intermediate Metabolizer | Abnormal/Priority/High Risk |
| ***CYP2D6*** | *29, *29x2 | no_sv,cnv2 | 0.09 | 1 | Normal Metabolizer | Normal/Routine/ Low Risk |
| ***CYP2D6*** | *28, *43 | no_sv,no_sv | 0.09 | 1 | Indeterminate | none |
| ***CYP2D6*** | *28, *35 | no_sv,no_sv | 0.09 | 1 | Indeterminate | none |
| ***CYP2D6*** | *2, *6 | no_sv,no_sv | 0.09 | 1 | Intermediate Metabolizer | Abnormal/Priority/High Risk |
| ***CYP2D6*** | *2, *4x2 | no_sv,cnv2 | 0.09 | 1 | Intermediate Metabolizer | Abnormal/Priority/High Risk |
| ***CYP2D6*** | ., . | no_sv,gc_e9 | 0.09 | 1 | Indeterminate | none |
| ***CYP2E1*** | *1, *1 | no_sv,no_sv | 47.14 | 552 | NA | NA |
| ***CYP2E1*** | *1, *7 | no_sv,no_sv | 33.13 | 388 | NA | NA |
| ***CYP2E1*** | *7, *7 | no_sv,no_sv | 10.67 | 125 | NA | NA |
| ***CYP2E1*** | *1x2, *7 | no_sv,cnv2 | 2.22 | 26 | NA | NA |
| ***CYP2E1*** | *1, *7x2 | no_sv,cnv2 | 1.96 | 23 | NA | NA |
| ***CYP2E1*** | ., . | no_sv,dup_e7e9 | 1.28 | 15 | NA | NA |
| ***CYP2E1*** | *1, *S1 | no_sv,dup_e7e9 | 0.6 | 7 | NA | NA |
| ***CYP2E1*** | *7, *7x2 | no_sv,cnv2 | 0.51 | 6 | NA | NA |
| ***CYP2E1*** | ., . | cnv2,dup_e7e9 | 0.51 | 6 | NA | NA |
| ***CYP2E1*** | *S1, *7 | no_sv,dup_e7e9 | 0.43 | 5 | NA | NA |
| ***CYP2E1*** | *4, *7 | no_sv,no_sv | 0.34 | 4 | NA | NA |
| ***CYP2E1*** | *1, *7x3 | no_sv,cnv3 | 0.26 | 3 | NA | NA |
| ***CYP2E1*** | *1x2, *7x2 | no_sv,cnv3 | 0.17 | 2 | NA | NA |
| ***CYP2E1*** | *7, *7x3 | no_sv,cnv3 | 0.17 | 2 | NA | NA |
| ***CYP2E1*** | *7, *DEL | no_sv,cnv0 | 0.17 | 2 | NA | NA |
| ***CYP2E1*** | *1, *DEL | no_sv,cnv0 | 0.09 | 1 | NA | NA |
| ***CYP2E1*** | *1x3, *7 | no_sv,cnv3 | 0.09 | 1 | NA | NA |
| ***CYP2E1*** | *1, *4 | no_sv,no_sv | 0.09 | 1 | NA | NA |
| ***CYP2E1*** | ., . | dup_e7e9,dup_e7e9 | 0.09 | 1 | NA | NA |
| ***CYP2E1*** | ., . | no_sv,cnv2 | 0.09 | 1 | NA | NA |
| ***CYP2F1*** | *1, *1 | no_sv,no_sv | 39.71 | 465 | NA | NA |
| ***CYP2F1*** | *1, *2 | no_sv,no_sv | 28.27 | 331 | NA | NA |
| ***CYP2F1*** | *1, *5 | no_sv,no_sv | 12.04 | 141 | NA | NA |
| ***CYP2F1*** | *1, *4 | no_sv,no_sv | 6.23 | 73 | NA | NA |
| ***CYP2F1*** | *2, *2 | no_sv,no_sv | 5.47 | 64 | NA | NA |
| ***CYP2F1*** | *1, *6 | no_sv,no_sv | 1.54 | 18 | NA | NA |
| ***CYP2F1*** | *2, *DEL | no_sv,cnv0 | 1.45 | 17 | NA | NA |
| ***CYP2F1*** | *2, *4 | no_sv,no_sv | 1.28 | 15 | NA | NA |
| ***CYP2F1*** | *4, *4 | no_sv,no_sv | 0.94 | 11 | NA | NA |
| ***CYP2F1*** | *4, *5 | no_sv,no_sv | 0.77 | 9 | NA | NA |
| ***CYP2F1*** | *5, *5 | no_sv,no_sv | 0.51 | 6 | NA | NA |
| ***CYP2F1*** | *2, *5 | no_sv,no_sv | 0.43 | 5 | NA | NA |
| ***CYP2F1*** | *1, *DEL | no_sv,cnv0 | 0.43 | 5 | NA | NA |
| ***CYP2F1*** | *5, *DEL | no_sv,cnv0 | 0.34 | 4 | NA | NA |
| ***CYP2F1*** | *5, *6 | no_sv,no_sv | 0.26 | 3 | NA | NA |
| ***CYP2F1*** | *1x2, *5 | no_sv,cnv2 | 0.09 | 1 | NA | NA |
| ***CYP2F1*** | *1, *1x2 | no_sv,cnv2 | 0.09 | 1 | NA | NA |
| ***CYP2F1*** | *4, *6 | no_sv,no_sv | 0.09 | 1 | NA | NA |
| ***CYP2F1*** | *4, *DEL | no_sv,cnv0 | 0.09 | 1 | NA | NA |
| ***CYP2J2*** | *1, *1 | no_sv,no_sv | 85.4 | 1000 | NA | NA |
| ***CYP2J2*** | *1, *7 | no_sv,no_sv | 12.81 | 150 | NA | NA |
| ***CYP2J2*** | *1, *1x2 | no_sv,cnv2 | 0.85 | 10 | NA | NA |
| ***CYP2J2*** | *7, *7 | no_sv,no_sv | 0.51 | 6 | NA | NA |
| ***CYP2J2*** | *1, *5 | no_sv,no_sv | 0.26 | 3 | NA | NA |
| ***CYP2J2*** | *5, *7 | no_sv,no_sv | 0.09 | 1 | NA | NA |
| ***CYP2J2*** | *7, *7x2 | no_sv,cnv2 | 0.09 | 1 | NA | NA |
| ***CYP2R1*** | *1, *1 | no_sv,no_sv | 99.83 | 1169 | NA | NA |
| ***CYP2R1*** | *1, *2 | no_sv,no_sv | 0.09 | 1 | NA | NA |
| ***CYP2R1*** | *1, *DEL | no_sv,cnv0 | 0.09 | 1 | NA | NA |
| ***CYP2S1*** | *1, *1 | no_sv,no_sv | 93.68 | 1097 | NA | NA |
| ***CYP2S1*** | *1, *3 | no_sv,no_sv | 4.36 | 51 | NA | NA |
| ***CYP2S1*** | *1, *DEL | no_sv,cnv0 | 1.45 | 17 | NA | NA |
| ***CYP2S1*** | *1, *1x2 | no_sv,cnv2 | 0.26 | 3 | NA | NA |
| ***CYP2S1*** | *3, *DEL | no_sv,cnv0 | 0.17 | 2 | NA | NA |
| ***CYP2S1*** | *3, *3 | no_sv,no_sv | 0.09 | 1 | NA | NA |
| ***CYP2W1*** | *1, *1 | no_sv,no_sv | 53.2 | 623 | NA | NA |
| ***CYP2W1*** | *1, *6 | no_sv,no_sv | 19.9 | 233 | NA | NA |
| ***CYP2W1*** | *1, *2 | no_sv,no_sv | 11.44 | 134 | NA | NA |
| ***CYP2W1*** | *1, *DEL | no_sv,cnv0 | 3.67 | 43 | NA | NA |
| ***CYP2W1*** | *6, *DEL | no_sv,cnv0 | 2.65 | 31 | NA | NA |
| ***CYP2W1*** | *6, *6 | no_sv,no_sv | 2.48 | 29 | NA | NA |
| ***CYP2W1*** | *2, *DEL | no_sv,cnv0 | 1.88 | 22 | NA | NA |
| ***CYP2W1*** | *2, *6 | no_sv,no_sv | 1.79 | 21 | NA | NA |
| ***CYP2W1*** | *1, *4 | no_sv,no_sv | 1.54 | 18 | NA | NA |
| ***CYP2W1*** | *2, *2 | no_sv,no_sv | 0.77 | 9 | NA | NA |
| ***CYP2W1*** | *4, *6 | no_sv,no_sv | 0.43 | 5 | NA | NA |
| ***CYP2W1*** | *1x2, *6 | no_sv,cnv2 | 0.09 | 1 | NA | NA |
| ***CYP2W1*** | *2x2, *6 | no_sv,cnv2 | 0.09 | 1 | NA | NA |
| ***CYP2W1*** | *4, *DEL | no_sv,cnv0 | 0.09 | 1 | NA | NA |
| ***CYP3A4*** | *1, *1 | no_sv,no_sv | 68.06 | 797 | NA | NA |
| ***CYP3A4*** | *1, *1B | no_sv,no_sv | 21.43 | 251 | NA | NA |
| ***CYP3A4*** | *1B, *1B | no_sv,no_sv | 4.78 | 56 | NA | NA |
| ***CYP3A4*** | *1, *22 | no_sv,no_sv | 3.42 | 40 | NA | NA |
| ***CYP3A4*** | *1, *3 | no_sv,no_sv | 0.51 | 6 | NA | NA |
| ***CYP3A4*** | *1, *1x2 | no_sv,cnv2 | 0.26 | 3 | NA | NA |
| ***CYP3A4*** | *1B, *22 | no_sv,no_sv | 0.26 | 3 | NA | NA |
| ***CYP3A4*** | *22, *22 | no_sv,no_sv | 0.26 | 3 | NA | NA |
| ***CYP3A4*** | *1, *10 | no_sv,no_sv | 0.17 | 2 | NA | NA |
| ***CYP3A4*** | *1, *12 | no_sv,no_sv | 0.17 | 2 | NA | NA |
| ***CYP3A4*** | *1, *1Bx2 | no_sv,cnv2 | 0.09 | 1 | NA | NA |
| ***CYP3A4*** | *1, *20 | no_sv,no_sv | 0.09 | 1 | NA | NA |
| ***CYP3A4*** | *1, *18 | no_sv,no_sv | 0.09 | 1 | NA | NA |
| ***CYP3A4*** | *1, *DEL | no_sv,cnv0 | 0.09 | 1 | NA | NA |
| ***CYP3A4*** | *1B, *10 | no_sv,no_sv | 0.09 | 1 | NA | NA |
| ***CYP3A4*** | *1B, *1Bx2 | no_sv,cnv2 | 0.09 | 1 | NA | NA |
| ***CYP3A4*** | *1B, *1x2 | no_sv,cnv2 | 0.09 | 1 | NA | NA |
| ***CYP3A4*** | *1B, *3 | no_sv,no_sv | 0.09 | 1 | NA | NA |
| ***CYP3A43*** | *1, *1 | no_sv,no_sv | 64.05 | 750 | NA | NA |
| ***CYP3A43*** | *1, *2B | no_sv,no_sv | 12.04 | 141 | NA | NA |
| ***CYP3A43*** | *1, *1B | no_sv,no_sv | 8.88 | 104 | NA | NA |
| ***CYP3A43*** | *1, *3 | no_sv,no_sv | 7.09 | 83 | NA | NA |
| ***CYP3A43*** | *1, *2A | no_sv,no_sv | 3.42 | 40 | NA | NA |
| ***CYP3A43*** | *1B, *2B | no_sv,no_sv | 0.94 | 11 | NA | NA |
| ***CYP3A43*** | *2B, *3 | no_sv,no_sv | 0.68 | 8 | NA | NA |
| ***CYP3A43*** | *1B, *3 | no_sv,no_sv | 0.6 | 7 | NA | NA |
| ***CYP3A43*** | *2B, *2B | no_sv,no_sv | 0.6 | 7 | NA | NA |
| ***CYP3A43*** | *2A, *2B | no_sv,no_sv | 0.43 | 5 | NA | NA |
| ***CYP3A43*** | *1B, *1B | no_sv,no_sv | 0.26 | 3 | NA | NA |
| ***CYP3A43*** | *2A, *3 | no_sv,no_sv | 0.17 | 2 | NA | NA |
| ***CYP3A43*** | *3, *3 | no_sv,no_sv | 0.17 | 2 | NA | NA |
| ***CYP3A43*** | *1, *1x2 | no_sv,cnv2 | 0.17 | 2 | NA | NA |
| ***CYP3A43*** | *1x2, *3 | no_sv,cnv2 | 0.09 | 1 | NA | NA |
| ***CYP3A43*** | *1, *3x2 | no_sv,cnv2 | 0.09 | 1 | NA | NA |
| ***CYP3A43*** | *1, *2Bx2 | no_sv,cnv2 | 0.09 | 1 | NA | NA |
| ***CYP3A43*** | *1, *1Bx2 | no_sv,cnv2 | 0.09 | 1 | NA | NA |
| ***CYP3A43*** | *2B, *DEL | no_sv,cnv0 | 0.09 | 1 | NA | NA |
| ***CYP3A43*** | *1B, *2A | no_sv,no_sv | 0.09 | 1 | NA | NA |
| ***CYP3A5*** | *3, *3 | no_sv,no_sv | 60.97 | 714 | Poor Metabolizer | Normal/Routine/Low Risk |
| ***CYP3A5*** | *1, *3 | no_sv,no_sv | 24.34 | 285 | Intermediate Metabolizer | Abnormal/Priority/High Risk |
| ***CYP3A5*** | *1, *1 | no_sv,no_sv | 4.53 | 53 | Normal Metabolizer | Abnormal/Priority/High Risk |
| ***CYP3A5*** | *3, *6 | no_sv,no_sv | 3.5 | 41 | Poor Metabolizer | Normal/Routine/Low Risk |
| ***CYP3A5*** | *3, *7 | no_sv,no_sv | 2.39 | 28 | Poor Metabolizer | Normal/Routine/Low Risk |
| ***CYP3A5*** | *1, *6 | no_sv,no_sv | 1.71 | 20 | Intermediate Metabolizer | Abnormal/Priority/High Risk |
| ***CYP3A5*** | *1, *7 | no_sv,no_sv | 1.37 | 16 | Intermediate Metabolizer | Abnormal/Priority/High Risk |
| ***CYP3A5*** | *6, *6 | no_sv,no_sv | 0.34 | 4 | Poor Metabolizer | Normal/Routine/Low Risk |
| ***CYP3A5*** | *3, *3x2 | no_sv,cnv2 | 0.26 | 3 | Indeterminate | None |
| ***CYP3A5*** | *6, *7 | no_sv,no_sv | 0.17 | 2 | Poor Metabolizer | Normal/Routine/Low Risk |
| ***CYP3A5*** | *7, *7 | no_sv,no_sv | 0.17 | 2 | Poor Metabolizer | Normal/Routine/Low Risk |
| ***CYP3A5*** | *1x2, *3 | no_sv,cnv2 | 0.09 | 1 | Indeterminate | None |
| ***CYP3A5*** | *3, *DEL | no_sv,cnv0 | 0.09 | 1 | Indeterminate | None |
| ***CYP3A5*** | *6x2, *7 | no_sv,cnv2 | 0.09 | 1 | Indeterminate | None |
| ***CYP3A7*** | *1, *1 | no_sv,no_sv | 62 | 726 | NA | NA |
| ***CYP3A7*** | *1, *2 | no_sv,no_sv | 27.07 | 317 | NA | NA |
| ***CYP3A7*** | *2, *2 | no_sv,no_sv | 4.87 | 57 | NA | NA |
| ***CYP3A7*** | *1, *1E | no_sv,no_sv | 3.07 | 36 | NA | NA |
| ***CYP3A7*** | *1E, *2 | no_sv,no_sv | 1.11 | 13 | NA | NA |
| ***CYP3A7*** | *1, *1D | no_sv,no_sv | 0.77 | 9 | NA | NA |
| ***CYP3A7*** | *1, *1B | no_sv,no_sv | 0.26 | 3 | NA | NA |
| ***CYP3A7*** | *1, *1x2 | no_sv,cnv2 | 0.26 | 3 | NA | NA |
| ***CYP3A7*** | *1E, *1E | no_sv,no_sv | 0.17 | 2 | NA | NA |
| ***CYP3A7*** | *1, *2x2 | no_sv,cnv2 | 0.09 | 1 | NA | NA |
| ***CYP3A7*** | *1, *DEL | no_sv,cnv0 | 0.09 | 1 | NA | NA |
| ***CYP3A7*** | *1B, *2 | no_sv,no_sv | 0.09 | 1 | NA | NA |
| ***CYP3A7*** | *1D, *1D | no_sv,no_sv | 0.09 | 1 | NA | NA |
| ***CYP3A7*** | *2, *2x2 | no_sv,cnv2 | 0.09 | 1 | NA | NA |
| ***CYP4F2*** | *1, *1 | no_sv,no_sv | 43.38 | 508 | Lower warfarin dose phenotype^¥^ | NA |
| ***CYP4F2*** | *1, *3 | no_sv,no_sv | 36.64 | 429 | Higher warfarin dose phenotype^¥^ | NA |
| ***CYP4F2*** | *3, *3 | no_sv,no_sv | 9.14 | 107 | Higher warfarin dose phenotype^¥^ | NA |
| ***CYP4F2*** | *1, *2 | no_sv,no_sv | 6.58 | 77 | Lower warfarin dose phenotype^¥^ | NA |
| ***CYP4F2*** | *2, *3 | no_sv,no_sv | 3.07 | 36 | Higher warfarin dose phenotype^¥^ | NA |
| ***CYP4F2*** | *2, *2 | no_sv,no_sv | 0.51 | 6 | Lower warfarin dose phenotype^¥^ | NA |
| ***CYP4F2*** | *1, *1x2 | no_sv,cnv2 | 0.17 | 2 | Indeterminate | NA |
| ***CYP4F2*** | *1, *3x2 | no_sv,cnv2 | 0.17 | 2 | Indeterminate | NA |
| ***CYP4F2*** | *1, *DEL | no_sv,cnv0 | 0.09 | 1 | Indeterminate | NA |
| ***CYP4F2*** | *1x2, *2 | no_sv,cnv2 | 0.09 | 1 | Indeterminate | NA |
| ***CYP4F2*** | *2x2, *3 | no_sv,cnv2 | 0.09 | 1 | Indeterminate | NA |
| ***CYP4F2*** | *3, *DEL | no_sv,cnv0 | 0.09 | 1 | Indeterminate | NA |
| ***G6PD*** | *1, *1 | no_sv,no_sv | 53.71 | 629 | NA | NA |
| ***G6PD*** | *1, *DEL | no_sv,cnv0 | 33.65 | 394 | NA | NA |
| ***G6PD*** | *1, *2 | no_sv,no_sv | 4.87 | 57 | NA | NA |
| ***G6PD*** | *1, *3 | no_sv,no_sv | 3.42 | 40 | NA | NA |
| ***G6PD*** | *3, *DEL | no_sv,cnv0 | 1.54 | 18 | NA | NA |
| ***G6PD*** | *2, *DEL | no_sv,cnv0 | 1.37 | 16 | NA | NA |
| ***G6PD*** | *2, *3 | no_sv,no_sv | 0.51 | 6 | NA | NA |
| ***G6PD*** | *3, *3 | no_sv,no_sv | 0.51 | 6 | NA | NA |
| ***G6PD*** | *1, *179 | no_sv,no_sv | 0.09 | 1 | NA | NA |
| ***G6PD*** | *1, *29 | no_sv,no_sv | 0.09 | 1 | NA | NA |
| ***G6PD*** | *1, *2x2 | no_sv,cnv2 | 0.09 | 1 | NA | NA |
| ***G6PD*** | *2, *2 | no_sv,no_sv | 0.09 | 1 | NA | NA |
| ***G6PD*** | *3, *5 | no_sv,no_sv | 0.09 | 1 | NA | NA |
| ***GSTM1*** | *2, *2 | cnv0,cnv0 | 43.72 | 512 | NA | NA |
| ***GSTM1*** | *1, *2 | no_sv,cnv0 | 28.35 | 332 | NA | NA |
| ***GSTM1*** | *2, *3 | no_sv,cnv0 | 14.6 | 171 | NA | NA |
| ***GSTM1*** | *1, *1 | no_sv,no_sv | 6.83 | 80 | NA | NA |
| ***GSTM1*** | *1, *3 | no_sv,no_sv | 4.61 | 54 | NA | NA |
| ***GSTM1*** | *3, *3 | no_sv,no_sv | 1.45 | 17 | NA | NA |
| ***GSTM1*** | *1, *1x2 | no_sv,cnv2 | 0.17 | 2 | NA | NA |
| ***GSTM1*** | *1x2, *3 | no_sv,cnv2 | 0.17 | 2 | NA | NA |
| ***GSTM1*** | *1, *3x2 | no_sv,cnv2 | 0.09 | 1 | NA | NA |
| ***GSTP1*** | *1, *2 | no_sv,no_sv | 41.84 | 490 | NA | NA |
| ***GSTP1*** | *1, *1 | no_sv,no_sv | 38.43 | 450 | NA | NA |
| ***GSTP1*** | *2, *2 | no_sv,no_sv | 9.56 | 112 | NA | NA |
| ***GSTP1*** | *1, *3 | no_sv,no_sv | 4.01 | 47 | NA | NA |
| ***GSTP1*** | *2, *3 | no_sv,no_sv | 2.9 | 34 | NA | NA |
| ***GSTP1*** | *1, *DEL | no_sv,cnv0 | 1.28 | 15 | NA | NA |
| ***GSTP1*** | *2, *DEL | no_sv,cnv0 | 1.02 | 12 | NA | NA |
| ***GSTP1*** | *2, *4 | no_sv,no_sv | 0.43 | 5 | NA | NA |
| ***GSTP1*** | *1, *1x2 | no_sv,cnv2 | 0.17 | 2 | NA | NA |
| ***GSTP1*** | *3, *3 | no_sv,no_sv | 0.17 | 2 | NA | NA |
| ***GSTP1*** | *1x2, *2 | no_sv,cnv2 | 0.09 | 1 | NA | NA |
| ***GSTP1*** | *3, *DEL | no_sv,cnv0 | 0.09 | 1 | NA | NA |
| ***NAT1*** | *1, *1 | no_sv,no_sv | 42.02 | 492 | NA | NA |
| ***NAT1*** | *1, *10 | no_sv,no_sv | 35.95 | 421 | NA | NA |
| ***NAT1*** | *10, *10 | no_sv,no_sv | 9.39 | 110 | NA | NA |
| ***NAT1*** | *1, *3 | no_sv,no_sv | 2.48 | 29 | NA | NA |
| ***NAT1*** | *1, *14 | no_sv,no_sv | 2.22 | 26 | NA | NA |
| ***NAT1*** | *1, *11 | no_sv,no_sv | 2.13 | 25 | NA | NA |
| ***NAT1*** | *10, *11 | no_sv,no_sv | 0.94 | 11 | NA | NA |
| ***NAT1*** | *1, *15 | no_sv,no_sv | 0.94 | 11 | NA | NA |
| ***NAT1*** | *3, *10 | no_sv,no_sv | 0.68 | 8 | NA | NA |
| ***NAT1*** | *1, *27 | no_sv,no_sv | 0.43 | 5 | NA | NA |
| ***NAT1*** | *10, *14 | no_sv,no_sv | 0.43 | 5 | NA | NA |
| ***NAT1*** | *1, *22 | no_sv,no_sv | 0.26 | 3 | NA | NA |
| ***NAT1*** | *1, *19 | no_sv,no_sv | 0.26 | 3 | NA | NA |
| ***NAT1*** | *10, *15 | no_sv,no_sv | 0.26 | 3 | NA | NA |
| ***NAT1*** | *10, *17 | no_sv,no_sv | 0.26 | 3 | NA | NA |
| ***NAT1*** | *10, *30 | no_sv,no_sv | 0.26 | 3 | NA | NA |
| ***NAT1*** | *1, *17 | no_sv,no_sv | 0.17 | 2 | NA | NA |
| ***NAT1*** | *1, *10x2 | no_sv,cnv2 | 0.17 | 2 | NA | NA |
| ***NAT1*** | *10, *27 | no_sv,no_sv | 0.17 | 2 | NA | NA |
| ***NAT1*** | *11, *11 | no_sv,no_sv | 0.17 | 2 | NA | NA |
| ***NAT1*** | *1, *1x2 | no_sv,cnv2 | 0.09 | 1 | NA | NA |
| ***NAT1*** | *10, *10x2 | no_sv,cnv2 | 0.09 | 1 | NA | NA |
| ***NAT1*** | *10, *23 | no_sv,no_sv | 0.09 | 1 | NA | NA |
| ***NAT1*** | *1x2, *10 | no_sv,cnv2 | 0.09 | 1 | NA | NA |
| ***NAT1*** | *1, *DEL | no_sv,cnv0 | 0.09 | 1 | NA | NA |
| ***NAT2*** | *5, *6 | no_sv,no_sv | 19.39 | 227 | NA | NA |
| ***NAT2*** | *5, *5 | no_sv,no_sv | 18.45 | 216 | NA | NA |
| ***NAT2*** | *1, *5 | no_sv,no_sv | 18.1 | 212 | NA | NA |
| ***NAT2*** | *1, *6 | no_sv,no_sv | 10.33 | 121 | NA | NA |
| ***NAT2*** | *1, *1 | no_sv,no_sv | 7.09 | 83 | NA | NA |
| ***NAT2*** | *6, *6 | no_sv,no_sv | 6.49 | 76 | NA | NA |
| ***NAT2*** | *5, *7 | no_sv,no_sv | 3.42 | 40 | NA | NA |
| ***NAT2*** | *5, *12 | no_sv,no_sv | 2.9 | 34 | NA | NA |
| ***NAT2*** | *1, *7 | no_sv,no_sv | 2.22 | 26 | NA | NA |
| ***NAT2*** | *6, *7 | no_sv,no_sv | 2.13 | 25 | NA | NA |
| ***NAT2*** | *1, *12 | no_sv,no_sv | 1.2 | 14 | NA | NA |
| ***NAT2*** | *1, *13 | no_sv,no_sv | 1.11 | 13 | NA | NA |
| ***NAT2*** | *5, *14 | no_sv,no_sv | 1.02 | 12 | NA | NA |
| ***NAT2*** | *6, *12 | no_sv,no_sv | 1.02 | 12 | NA | NA |
| ***NAT2*** | *6, *14 | no_sv,no_sv | 0.94 | 11 | NA | NA |
| ***NAT2*** | *6, *13 | no_sv,no_sv | 0.94 | 11 | NA | NA |
| ***NAT2*** | *5, *13 | no_sv,no_sv | 0.77 | 9 | NA | NA |
| ***NAT2*** | *1, *14 | no_sv,no_sv | 0.77 | 9 | NA | NA |
| ***NAT2*** | *5, *11 | no_sv,no_sv | 0.17 | 2 | NA | NA |
| ***NAT2*** | *1x2, *6 | no_sv,cnv2 | 0.17 | 2 | NA | NA |
| ***NAT2*** | *5x2, *6 | no_sv,cnv2 | 0.17 | 2 | NA | NA |
| ***NAT2*** | *12, *13 | no_sv,no_sv | 0.17 | 2 | NA | NA |
| ***NAT2*** | *1, *1x2 | no_sv,cnv2 | 0.17 | 2 | NA | NA |
| ***NAT2*** | *7, *13 | no_sv,no_sv | 0.17 | 2 | NA | NA |
| ***NAT2*** | *7, *7 | no_sv,no_sv | 0.17 | 2 | NA | NA |
| ***NAT2*** | *5, *5x2 | no_sv,cnv2 | 0.09 | 1 | NA | NA |
| ***NAT2*** | *12, *14x2 | no_sv,cnv2 | 0.09 | 1 | NA | NA |
| ***NAT2*** | *12, *12 | no_sv,no_sv | 0.09 | 1 | NA | NA |
| ***NAT2*** | *1, *DEL | no_sv,cnv0 | 0.09 | 1 | NA | NA |
| ***NAT2*** | *6, *DEL | no_sv,cnv0 | 0.09 | 1 | NA | NA |
| ***NAT2*** | *6x2, *7 | no_sv,cnv2 | 0.09 | 1 | NA | NA |
| ***NUDT15*** | *1, *1 | no_sv,no_sv | 96.5 | 1130 | Normal Metabolizer | Normal/Routine/Low risk |
| ***NUDT15*** | *1, *3 | no_sv,no_sv | 2.13 | 25 | Intermediate Metabolizer | Abnormal/Priority/High Risk |
| ***NUDT15*** | *1, *4 | no_sv,no_sv | 0.94 | 11 | Indeterminate | Abnormal/Priority/High Risk |
| ***NUDT15*** | *1, *DEL | no_sv,cnv0 | 0.26 | 3 | Indeterminate | None |
| ***NUDT15*** | *1, *1x2 | no_sv,cnv2 | 0.09 | 1 | Indeterminate | None |
| ***NUDT15*** | *3, *3 | no_sv,no_sv | 0.09 | 1 | Poor Metabolizer | Abnormal/Priority/High Risk |
| ***POR*** | *1, *1 | no_sv,no_sv | 57.64 | 675 | NA | NA |
| ***POR*** | *1, *28 | no_sv,no_sv | 35.87 | 420 | NA | NA |
| ***POR*** | *28, *28 | no_sv,no_sv | 5.98 | 70 | NA | NA |
| ***POR*** | *28, *DEL | no_sv,cnv0 | 0.26 | 3 | NA | NA |
| ***POR*** | *1, *1x2 | no_sv,cnv2 | 0.17 | 2 | NA | NA |
| ***POR*** | *1, *DEL | no_sv,cnv0 | 0.09 | 1 | NA | NA |
| ***SLC15A2*** | *1, *2 | no_sv,no_sv | 47.14 | 552 | NA | NA |
| ***SLC15A2*** | *1, *1 | no_sv,no_sv | 35.27 | 413 | NA | NA |
| ***SLC15A2*** | *2, *2 | no_sv,no_sv | 17.42 | 204 | NA | NA |
| ***SLC15A2*** | *1, *DEL | no_sv,cnv0 | 0.09 | 1 | NA | NA |
| ***SLC15A2*** | *2, *DEL | no_sv,cnv0 | 0.09 | 1 | NA | NA |
| ***SLC22A2*** | *1, *2 | no_sv,no_sv | 27.07 | 317 | NA | NA |
| ***SLC22A2*** | *1, *3 | no_sv,no_sv | 18.19 | 213 | NA | NA |
| ***SLC22A2*** | *1, *1 | no_sv,no_sv | 16.74 | 196 | NA | NA |
| ***SLC22A2*** | *2, *3 | no_sv,no_sv | 16.05 | 188 | NA | NA |
| ***SLC22A2*** | *2, *2 | no_sv,no_sv | 13.75 | 161 | NA | NA |
| ***SLC22A2*** | *3, *3 | no_sv,no_sv | 4.53 | 53 | NA | NA |
| ***SLC22A2*** | *S1, *2 | no_sv,del_i9 | 0.68 | 8 | NA | NA |
| ***SLC22A2*** | *S2, *3 | no_sv,del_e11 | 0.6 | 7 | NA | NA |
| ***SLC22A2*** | *1, *S1 | no_sv,del_i9 | 0.51 | 6 | NA | NA |
| ***SLC22A2*** | *1, *6 | no_sv,no_sv | 0.34 | 4 | NA | NA |
| ***SLC22A2*** | *2, *7 | no_sv,no_sv | 0.34 | 4 | NA | NA |
| ***SLC22A2*** | *3, *6 | no_sv,no_sv | 0.26 | 3 | NA | NA |
| ***SLC22A2*** | *1, *7 | no_sv,no_sv | 0.26 | 3 | NA | NA |
| ***SLC22A2*** | *2, *6 | no_sv,no_sv | 0.17 | 2 | NA | NA |
| ***SLC22A2*** | *2, *S2 | no_sv,del_e11 | 0.17 | 2 | NA | NA |
| ***SLC22A2*** | *S1, *3 | no_sv,del_i9 | 0.09 | 1 | NA | NA |
| ***SLC22A2*** | *S1, *7 | no_sv,del_i9 | 0.09 | 1 | NA | NA |
| ***SLC22A2*** | *2, *4 | no_sv,no_sv | 0.09 | 1 | NA | NA |
| ***SLC22A2*** | ., . | no_sv,del_i9 | 0.09 | 1 | NA | NA |
| ***SLCO1B1*** | *1, *1 | no_sv,no_sv | 20.07 | 235 | Normal Function | Normal/Routine/Low Risk |
| ***SLCO1B1*** | *1, *1B | no_sv,no_sv | 14.01 | 164 | Normal Function | Normal/Routine/Low Risk |
| ***SLCO1B1*** | *1, *14 | no_sv,no_sv | 11.1 | 130 | Possible Increased Function | None |
| ***SLCO1B1*** | *1, *15 | no_sv,no_sv | 8.88 | 104 | Decreased Function | Abnormal/Priority/High Risk |
| ***SLCO1B1*** | *1B, *1B | no_sv,no_sv | 5.21 | 61 | Normal Function | Normal/Routine/Low Risk |
| ***SLCO1B1*** | *1, *35 | no_sv,no_sv | 4.78 | 56 | Possible Increased Function | None |
| ***SLCO1B1*** | *1B, *14 | no_sv,no_sv | 4.44 | 52 | Possible Increased Function | None |
| ***SLCO1B1*** | *14, *15 | no_sv,no_sv | 3.59 | 42 | Indeterminate | None |
| ***SLCO1B1*** | *1B, *15 | no_sv,no_sv | 3.16 | 37 | Decreased Function | Abnormal/Priority/High Risk |
| ***SLCO1B1*** | *1B, *35 | no_sv,no_sv | 2.48 | 29 | Possible Increased Function | None |
| ***SLCO1B1*** | *14, *14 | no_sv,no_sv | 1.79 | 21 | Possible Increased Function | None |
| ***SLCO1B1*** | *1, *21 | no_sv,no_sv | 1.45 | 17 | Indeterminate | None |
| ***SLCO1B1*** | *1, *5 | no_sv,no_sv | 1.37 | 16 | Decreased Function | Abnormal/Priority/High Risk |
| ***SLCO1B1*** | *14, *35 | no_sv,no_sv | 1.28 | 15 | Indeterminate | None |
| ***SLCO1B1*** | *1, *17 | no_sv,no_sv | 1.11 | 13 | Decreased Function | Abnormal/Priority/High Risk |
| ***SLCO1B1*** | *15, *35 | no_sv,no_sv | 0.94 | 11 | Indeterminate | None |
| ***SLCO1B1*** | *14, *17 | no_sv,no_sv | 0.77 | 9 | Indeterminate | None |
| ***SLCO1B1*** | *15, *15 | no_sv,no_sv | 0.77 | 9 | Poor Function | Abnormal/Priority/High Risk |
| ***SLCO1B1*** | *1, *27 | no_sv,no_sv | 0.68 | 8 | Indeterminate | None |
| ***SLCO1B1*** | *1, *S2 | no_sv,no_sv | 0.68 | 8 | Indeterminate | None |
| ***SLCO1B1*** | *1B, *17 | no_sv,no_sv | 0.6 | 7 | Decreased Function | Abnormal/Priority/High Risk |
| ***SLCO1B1*** | *1B, *5 | no_sv,no_sv | 0.6 | 7 | Decreased Function | Abnormal/Priority/High Risk |
| ***SLCO1B1*** | *1, *31 | no_sv,no_sv | 0.6 | 7 | Possible Decreased Function | Abnormal/Priority/High Risk |
| ***SLCO1B1*** | *35, *35 | no_sv,no_sv | 0.6 | 7 | Possible Increased Function | None |
| ***SLCO1B1*** | *1B, *21 | no_sv,no_sv | 0.51 | 6 | Indeterminate | None |
| ***SLCO1B1*** | *5, *15 | no_sv,no_sv | 0.51 | 6 | Poor Function | Abnormal/Priority/High Risk |
| ***SLCO1B1*** | *1B, *S2 | no_sv,no_sv | 0.43 | 5 | Indeterminate | None |
| ***SLCO1B1*** | *1B, *27 | no_sv,no_sv | 0.43 | 5 | Indeterminate | None |
| ***SLCO1B1*** | *15, *17 | no_sv,no_sv | 0.43 | 5 | Poor Function | Abnormal/Priority/High Risk |
| ***SLCO1B1*** | *14, *21 | no_sv,no_sv | 0.43 | 5 | Indeterminate | None |
| ***SLCO1B1*** | *1B, *31 | no_sv,no_sv | 0.34 | 4 | Possible Decreased Function | Abnormal/Priority/High Risk |
| ***SLCO1B1*** | *1B, *30 | no_sv,no_sv | 0.34 | 4 | Indeterminate | None |
| ***SLCO1B1*** | *15, *21 | no_sv,no_sv | 0.34 | 4 | Indeterminate | None |
| ***SLCO1B1*** | *5, *14 | no_sv,no_sv | 0.34 | 4 | Indeterminate | None |
| ***SLCO1B1*** | *1B, *19 | no_sv,no_sv | 0.34 | 4 | Indeterminate | None |
| ***SLCO1B1*** | *1, *1x2 | no_sv,cnv2 | 0.26 | 3 | Indeterminate | None |
| ***SLCO1B1*** | *14, *27 | no_sv,no_sv | 0.26 | 3 | Indeterminate | None |
| ***SLCO1B1*** | *15, *31 | no_sv,no_sv | 0.26 | 3 | Possible Poor Function | Abnormal/Priority/High Risk |
| ***SLCO1B1*** | *15, *27 | no_sv,no_sv | 0.26 | 3 | Indeterminate | None |
| ***SLCO1B1*** | *1, *30 | no_sv,no_sv | 0.26 | 3 | Indeterminate | None |
| ***SLCO1B1*** | *14, *30 | no_sv,no_sv | 0.17 | 2 | Indeterminate | None |
| ***SLCO1B1*** | *1x2, *35 | no_sv,cnv2 | 0.17 | 2 | Indeterminate | None |
| ***SLCO1B1*** | *5, *35 | no_sv,no_sv | 0.17 | 2 | Indeterminate | None |
| ***SLCO1B1*** | *1B, *DEL | no_sv,cnv0 | 0.17 | 2 | Indeterminate | None |
| ***SLCO1B1*** | *S2, *15 | no_sv,no_sv | 0.17 | 2 | Indeterminate | None |
| ***SLCO1B1*** | *21, *35 | no_sv,no_sv | 0.17 | 2 | Indeterminate | None |
| ***SLCO1B1*** | *14, *31 | no_sv,no_sv | 0.17 | 2 | Indeterminate | None |
| ***SLCO1B1*** | *S2, *21 | no_sv,no_sv | 0.17 | 2 | Indeterminate | None |
| ***SLCO1B1*** | *S2, *30 | no_sv,no_sv | 0.09 | 1 | Indeterminate | None |
| ***SLCO1B1*** | *9, *31 | no_sv,no_sv | 0.09 | 1 | Possible Poor Function | Abnormal/Priority/High Risk |
| ***SLCO1B1*** | *5, *DEL | no_sv,cnv0 | 0.09 | 1 | Indeterminate | None |
| ***SLCO1B1*** | *S2, *35 | no_sv,no_sv | 0.09 | 1 | Indeterminate | None |
| ***SLCO1B1*** | *S2x2, *14 | no_sv,cnv2 | 0.09 | 1 | Indeterminate | None |
| ***SLCO1B1*** | *5, *21 | no_sv,no_sv | 0.09 | 1 | Indeterminate | None |
| ***SLCO1B1*** | *1, *22 | no_sv,no_sv | 0.09 | 1 | Indeterminate | None |
| ***SLCO1B1*** | *1, *24 | no_sv,no_sv | 0.09 | 1 | Indeterminate | None |
| ***SLCO1B1*** | *35, *DEL | no_sv,cnv0 | 0.09 | 1 | Indeterminate | None |
| ***SLCO1B1*** | *1, *19 | no_sv,no_sv | 0.09 | 1 | Indeterminate | None |
| ***SLCO1B1*** | *30, *35 | no_sv,no_sv | 0.09 | 1 | Indeterminate | None |
| ***SLCO1B1*** | *24, *31 | no_sv,no_sv | 0.09 | 1 | Indeterminate | None |
| ***SLCO1B1*** | *22, *35 | no_sv,no_sv | 0.09 | 1 | Indeterminate | None |
| ***SLCO1B1*** | *17, *21 | no_sv,no_sv | 0.09 | 1 | Indeterminate | None |
| ***SLCO1B1*** | *1x2, *17 | no_sv,cnv2 | 0.09 | 1 | Indeterminate | None |
| ***SLCO1B1*** | *1x2, *15 | no_sv,cnv2 | 0.09 | 1 | Indeterminate | None |
| ***SLCO1B1*** | *1, *1Bx2 | no_sv,cnv2 | 0.09 | 1 | Indeterminate | None |
| ***SLCO1B1*** | *1, *DEL | no_sv,cnv0 | 0.09 | 1 | Indeterminate | None |
| ***SLCO1B1*** | *14, *DEL | no_sv,cnv0 | 0.09 | 1 | Indeterminate | None |
| ***SLCO1B1*** | *15, *19 | no_sv,no_sv | 0.09 | 1 | Indeterminate | None |
| ***SLCO1B1*** | *1B, *1x2 | no_sv,cnv2 | 0.09 | 1 | Indeterminate | None |
| ***SLCO1B1*** | *1B, *1Bx2 | no_sv,cnv2 | 0.09 | 1 | Indeterminate | None |
| ***SLCO1B1*** | ., . | no_sv,cnv2 | 0.09 | 1 | Indeterminate | None |
| ***SLCO2B1*** | *1, *1 | no_sv,no_sv | 77.2 | 904 | NA | NA |
| ***SLCO2B1*** | *1, *S464F | no_sv,no_sv | 18.53 | 217 | NA | NA |
| ***SLCO2B1*** | *1, *S1 | no_sv,no_sv | 2.13 | 25 | NA | NA |
| ***SLCO2B1*** | *S464F, *S464F | no_sv,no_sv | 1.62 | 19 | NA | NA |
| ***SLCO2B1*** | *S1, *S464F | no_sv,no_sv | 0.26 | 3 | NA | NA |
| ***SLCO2B1*** | *1, *1x2 | no_sv,cnv2 | 0.09 | 1 | NA | NA |
| ***SLCO2B1*** | *1, *DEL | no_sv,cnv0 | 0.09 | 1 | NA | NA |
| ***SLCO2B1*** | *1x2, *S464F | no_sv,cnv2 | 0.09 | 1 | NA | NA |
| ***TPMT*** | *1, *1 | no_sv,no_sv | 90.26 | 1057 | Normal Metabolizer | Normal/Routine/Low Risk |
| ***TPMT*** | *1, *3A | no_sv,no_sv | 2.82 | 33 | Intermediate Metabolizer | Abnormal/Priority/High Risk |
| ***TPMT*** | *1, *3C | no_sv,no_sv | 2.65 | 31 | Intermediate Metabolizer | Abnormal/Priority/High Risk |
| ***TPMT*** | *1, *2 | no_sv,no_sv | 1.45 | 17 | Intermediate Metabolizer | Abnormal/Priority/High Risk |
| ***TPMT*** | *1, *8 | no_sv,no_sv | 1.37 | 16 | Indeterminate | Abnormal/Priority/High Risk |
| ***TPMT*** | *3C, *3B | no_sv,no_sv | 0.77 | 9 | Poor Metabolizer | Abnormal/Priority/High Risk |
| ***TPMT*** | *1, *24 | no_sv,no_sv | 0.34 | 4 | Indeterminate | Abnormal/Priority/High Risk |
| ***TPMT*** | *2, *3A | no_sv,no_sv | 0.09 | 1 | Poor Metabolizer | Abnormal/Priority/High Risk |
| ***TPMT*** | *3A, *24 | no_sv,no_sv | 0.09 | 1 | Possible Intermediate Metabolizer | Abnormal/Priority/High Risk |
| ***TPMT*** | *3A, *3C | no_sv,no_sv | 0.09 | 1 | Poor Metabolizer | Abnormal/Priority/High Risk |
| ***TPMT*** | *8, *24 | no_sv,no_sv | 0.09 | 1 | Indeterminate | Abnormal/Priority/High Risk |
| ***UGT1A1*** | *28, *60 | no_sv,no_sv | 42.95 | 503 | Intermediate Metabolizer | Normal/Routine/Low Risk |
| ***UGT1A1*** | *1, *1 | no_sv,no_sv | 20.67 | 242 | Normal Metabolizer | Normal/Routine/Low Risk |
| ***UGT1A1*** | *1, *60 | no_sv,no_sv | 15.97 | 187 | Normal Metabolizer | Normal/Routine/Low Risk |
| ***UGT1A1*** | *28, *28 | no_sv,no_sv | 11.27 | 132 | Poor Metabolizer | Abnormal/Priority/High Risk |
| ***UGT1A1*** | *60, *60 | no_sv,no_sv | 3.93 | 46 | Normal Metabolizer | Normal/Routine/Low Risk |
| ***UGT1A1*** | *37, *60 | no_sv,no_sv | 2.31 | 27 | Intermediate Metabolizer | Normal/Routine/Low Risk |
| ***UGT1A1*** | *36, *60 | no_sv,no_sv | 0.77 | 9 | Normal Metabolizer | Normal/Routine/Low Risk |
| ***UGT1A1*** | *1, *6 | no_sv,no_sv | 0.68 | 8 | Intermediate Metabolizer | Normal/Routine/Low Risk |
| ***UGT1A1*** | *6, *60 | no_sv,no_sv | 0.6 | 7 | Intermediate Metabolizer | Normal/Routine/Low Risk |
| ***UGT1A1*** | *1, *36 | no_sv,no_sv | 0.26 | 3 | Normal Metabolizer | Normal/Routine/Low Risk |
| ***UGT1A1*** | *1, *28 | no_sv,no_sv | 0.17 | 2 | Intermediate Metabolizer | Normal/Routine/Low Risk |
| ***UGT1A1*** | *1, *37 | no_sv,no_sv | 0.17 | 2 | Intermediate Metabolizer | Normal/Routine/Low Risk |
| ***UGT1A1*** | *28, *28x2 | no_sv,cnv2 | 0.17 | 2 | Indeterminate | none |
| ***UGT1A1*** | *1, *7 | no_sv,no_sv | 0.09 | 1 | Indeterminate | none |
| ***UGT2B15*** | *1, *2 | no_sv,no_sv | 14.09 | 165 | NA | NA |
| ***UGT2B15*** | *1, *4 | no_sv,no_sv | 13.49 | 158 | NA | NA |
| ***UGT2B15*** | *2, *4 | no_sv,no_sv | 12.47 | 146 | NA | NA |
| ***UGT2B15*** | *4, *5 | no_sv,no_sv | 12.21 | 143 | NA | NA |
| ***UGT2B15*** | *2, *5 | no_sv,no_sv | 9.74 | 114 | NA | NA |
| ***UGT2B15*** | *1, *5 | no_sv,no_sv | 9.31 | 109 | NA | NA |
| ***UGT2B15*** | *4, *4 | no_sv,no_sv | 8.11 | 95 | NA | NA |
| ***UGT2B15*** | *1, *1 | no_sv,no_sv | 7.51 | 88 | NA | NA |
| ***UGT2B15*** | *2, *2 | no_sv,no_sv | 6.4 | 75 | NA | NA |
| ***UGT2B15*** | *5, *5 | no_sv,no_sv | 4.27 | 50 | NA | NA |
| ***UGT2B15*** | *2x2, *4 | no_sv,cnv2 | 0.17 | 2 | NA | NA |
| ***UGT2B15*** | *S1, *5 | no_sv,del_i3e6 | 0.17 | 2 | NA | NA |
| ***UGT2B15*** | *S1, *4 | no_sv,del_i3e6 | 0.17 | 2 | NA | NA |
| ***UGT2B15*** | *S1, *2 | no_sv,del_i3e6 | 0.17 | 2 | NA | NA |
| ***UGT2B15*** | *5, *DEL | no_sv,cnv0 | 0.17 | 2 | NA | NA |
| ***UGT2B15*** | *2x2, *5 | no_sv,cnv2 | 0.17 | 2 | NA | NA |
| ***UGT2B15*** | *2, *5x2 | no_sv,cnv2 | 0.17 | 2 | NA | NA |
| ***UGT2B15*** | *2, *4x2 | no_sv,cnv2 | 0.17 | 2 | NA | NA |
| ***UGT2B15*** | *1, *DEL | no_sv,cnv0 | 0.17 | 2 | NA | NA |
| ***UGT2B15*** | *1, *5x2 | no_sv,cnv2 | 0.17 | 2 | NA | NA |
| ***UGT2B15*** | *2, *DEL | no_sv,cnv0 | 0.09 | 1 | NA | NA |
| ***UGT2B15*** | *2, *6 | no_sv,no_sv | 0.09 | 1 | NA | NA |
| ***UGT2B15*** | *2, *2x2 | no_sv,cnv2 | 0.09 | 1 | NA | NA |
| ***UGT2B15*** | *4, *DEL | no_sv,cnv0 | 0.09 | 1 | NA | NA |
| ***UGT2B15*** | *1x2, *2 | no_sv,cnv2 | 0.09 | 1 | NA | NA |
| ***UGT2B15*** | *1, *S1 | no_sv,del_i3e6 | 0.09 | 1 | NA | NA |
| ***UGT2B15*** | *1, *2x2 | no_sv,cnv2 | 0.09 | 1 | NA | NA |
| ***UGT2B15*** | ., . | cnv0,del_i3e6 | 0.09 | 1 | NA | NA |
| ***UGT2B17*** | *1, *1 | no_sv,no_sv | 44.83 | 525 | NA | NA |
| ***UGT2B17*** | *1, *2 | no_sv,cnv0 | 41.67 | 488 | NA | NA |
| ***UGT2B17*** | *2, *2 | cnv0,cnv0 | 13.49 | 158 | NA | NA |
| ***UGT2B7*** | *1, *2 | no_sv,no_sv | 46.54 | 545 | NA | NA |
| ***UGT2B7*** | *1, *1 | no_sv,no_sv | 31.85 | 373 | NA | NA |
| ***UGT2B7*** | *2, *2 | no_sv,no_sv | 16.82 | 197 | NA | NA |
| ***UGT2B7*** | *1, *1x2 | no_sv,cnv2 | 1.88 | 22 | NA | NA |
| ***UGT2B7*** | *1, *2x2 | no_sv,cnv2 | 0.68 | 8 | NA | NA |
| ***UGT2B7*** | *1, *3 | no_sv,no_sv | 0.6 | 7 | NA | NA |
| ***UGT2B7*** | *1x2, *2 | no_sv,cnv2 | 0.51 | 6 | NA | NA |
| ***UGT2B7*** | *2, *2x2 | no_sv,cnv2 | 0.51 | 6 | NA | NA |
| ***UGT2B7*** | *2, *3 | no_sv,no_sv | 0.34 | 4 | NA | NA |
| ***UGT2B7*** | *2, *DEL | no_sv,cnv0 | 0.17 | 2 | NA | NA |
| ***UGT2B7*** | *3, *3 | no_sv,no_sv | 0.09 | 1 | NA | NA |
| ***VKORC1*** | *2, *3 | no_sv,no_sv | 19.73 | 231 | Decreased warfarin dose phenotype^¥^ | NA |
| ***VKORC1*** | *2, *2 | no_sv,no_sv | 14.18 | 166 | Decreased warfarin dose phenotype^¥^ | NA |
| ***VKORC1*** | *3, *3 | no_sv,no_sv | 13.15 | 154 | Indeterminate | NA |
| ***VKORC1*** | *3, *4 | no_sv,no_sv | 11.27 | 132 | Indeterminate | NA |
| ***VKORC1*** | *1, *3 | no_sv,no_sv | 10.25 | 120 | Indeterminate | NA |
| ***VKORC1*** | *2, *4 | no_sv,no_sv | 9.74 | 114 | Decreased warfarin dose phenotype^¥^ | NA |
| ***VKORC1*** | *1, *2 | no_sv,no_sv | 6.15 | 72 | Decreased warfarin dose phenotype^¥^ | NA |
| ***VKORC1*** | *1, *4 | no_sv,no_sv | 4.87 | 57 | Indeterminate | NA |
| ***VKORC1*** | *4, *4 | no_sv,no_sv | 3.07 | 36 | Indeterminate | NA |
| ***VKORC1*** | *1, *1 | no_sv,no_sv | 2.48 | 29 | Indeterminate | NA |
| ***VKORC1*** | *2, *DEL | no_sv,cnv0 | 2.13 | 25 | Indeterminate | NA |
| ***VKORC1*** | *3, *DEL | no_sv,cnv0 | 1.88 | 22 | Indeterminate | NA |
| ***VKORC1*** | *4, *DEL | no_sv,cnv0 | 0.43 | 5 | Indeterminate | NA |
| ***VKORC1*** | *1, *DEL | no_sv,cnv0 | 0.26 | 3 | Indeterminate | NA |
| ***VKORC1*** | *2x2, *3 | no_sv,cnv2 | 0.09 | 1 | Indeterminate | NA |
| ***VKORC1*** | *2, *2x2 | no_sv,cnv2 | 0.09 | 1 | Indeterminate | NA |
| ***VKORC1*** | *1, *V66M | no_sv,no_sv | 0.09 | 1 | Indeterminate | NA |
| ***VKORC1*** | *3, *V66M | no_sv,no_sv | 0.09 | 1 | Indeterminate | NA |
| ***VKORC1*** | *4, *V66M | no_sv,no_sv | 0.09 | 1 | Indeterminate | NA |

^¥^ Predicted phenotypes for VKORC1 and CYP4F2 were interpreted from Warfarin dosing guideline (Johnson et al., 2017a)

**Supplementary table 3.** Allele frequencies extracted from the WGS database (ABraOM).

| **gene** | **name** | **sv** | **hap_score** | **chr** | **snv** | **id** | **count** | **percentage** |
| --- | --- | --- | --- | --- | --- | --- | --- | --- |
| **CFTR** | ref | no_sv | Normal function |  |  | ref | 2322 | 0.99146 |
| **CFTR** | NA | no_sv | CFTR Class IV | 7 | 117614699G>C | rs75541969 | 2 | 0.000854 |
| **CFTR** | NA | no_sv | CFTR Class IV | 7 | 117530974C>T | rs77834169 | 2 | 0.000854 |
| **CFTR** | NA | no_sv | CFTR Class IV | 7 | 117530975G>A | rs78655421 | 2 | 0.000854 |
| **DPYD** | ref | no_sv | Normal function |  |  | ref | 791 | 0.343775 |
| **DPYD** | c.1905+1G>A (*2A) | no_sv | No function | 1 | 97450058C>T | rs3918290 | 3 | 0.001281 |
| **DPYD** | c.1129-5923C>G(HapB3) | no_sv | Decreased function | 1 | 97579893G>C | rs75017182 | 10 | 0.00427 |
| **DPYD** | c.1236G>A (HapB3) | no_sv | Decreased function | 1 | 97573863C>T | rs56038477 | 10 | 0.00427 |
| **DPYD** | c.2846A>T | no_sv | Decreased function | 1 | 97082391T>A | rs67376798 | 9 | 0.003843 |
| **DPYD** | c.557A>G | no_sv | Decreased function | 1 | 97699474T>C | rs115232898 | 6 | 0.002562 |
| **IFNL3** | ref | no_sv | Normal function |  |  | ref | 1479 | 0.631512 |
| **IFNL3** | NA | no_sv | Unfavorable Response | 19 | 39248147C>T | rs12979860 | 863 | 0.368488 |

**Supplementary Table 4.** Cohort medication use and predicted high-risk individuals.

| **Gene** | **Drug** | **ATC** | **CPIC Level** | **CPIC Level Status** | **PharmGKB**  **Level of Evidence** | **PGx on FDA Label** | **% taking drugs (n)** | **% taking drugs**  **at high risk (n)^¥^** |
| --- | --- | --- | --- | --- | --- | --- | --- | --- |
| ***SLCO1B1*** | simvastatin | C10AA01, C10BA02 | A | Final | 1A | Informative PGx | 20.14 (223) | 2.98 (33) |
| ***CYP2C19*** | omeprazole | A02BC01, A02BD06,  A02BD05 | A | Final | 1A | Actionable PGx | 19.51 (216) | 11.56 (128) |
| ***CYP2C19*** | amitriptyline | N06AA09, N06CA01 | A | Final | 1A |  | 3.25 (36) | 1.63 (18) |
| ***CYP2D6*** | amitriptyline | N06AA09, N06CA01 | A | Final | 1A | Actionable PGx | 3.25 (36) | 1.26 (14) |
| ***CYP2C19*** | sertraline | N06AB06 | B | Final | 1A |  | 2.26 (25) | 1.17 (13) |
| ***CYP2C9*** | ibuprofen | M01AE01 | A | Final | 1A |  | 2.17 (24) | 0.45 (5) |
| ***CYP2C9*** | warfarin | B01AA03 | A | Final | 1A | Actionable PGx | 1.36 (15) | 0.81 (9) |
| ***CYP4F2*** | warfarin | B01AA03 | A | Final | 1A |  | 1.36 (15) | 0.45 (5) |
| ***VKORC1*** | warfarin | B01AA03 | A | Final | 1A | Actionable PGx | 1.36 (15) | 0.54 (6) |
| ***CYP2C19*** | citalopram | N06AB04 | A | Final | 1A | Actionable PGx | 0.9 (10) | 0.54 (6) |
| ***CYP2C19*** | clopidogrel | B01AC04 | A | Final | 1A | Actionable PGx | 0.81 (9) | 0.27 (3) |
| ***CYP2D6*** | metoprolol | C07AB02, C07FX03,  C07FB13, C07FB02,  C07FX05, C07CB02,  C07BB02, C07BB52 | B/C | Provisional | 1A | Informative PGx | 0.63 (7) | 0.27 (3) |
| ***CYP2D6*** | tramadol | N02AX02, N02AJ13 | A | Final | 1A | Actionable PGx | 0.63 (7) | 0.18 (2) |
| ***CYP2C19*** | pantoprazole | A02BC02 | A | Final | 1A | Actionable PGx | 0.54 (6) | 0.36 (4) |
| ***CYP2D6*** | paroxetine | N06AB05 | A | Final | 1A | Informative PGx | 0.54 (6) | 0 (0) |
| ***CYP2C9*** | phenytoin | N03AB02 | A | Final | 1A | Actionable PGx | 0.54 (6) | 0.09 (1) |
| ***CYP2D6*** | propafenone | C01BC03 | B/C | Provisional | 1A | Actionable PGx | 0.54 (6) | 0.18 (2) |
| ***CYP2C9*** | meloxicam | M01AC06 | A | Final | 1A | Actionable PGx | 0.36 (4) | 0.18 (2) |
| ***CYP2C19*** | clomipramine | N06AA04 | B | Final | 1A |  | 0.27 (3) | 0.18 (2) |
| ***CYP2D6*** | clomipramine | N06AA04 | B | Final | 1A | Actionable PGx | 0.27 (3) | 0.18 (2) |
| ***CYP2C19*** | lansoprazole | A02BC03 | A | Final | 1A | Informative PGx | 0.27 (3) | 0.18 (2) |
| ***CYP2D6*** | nortriptyline | N06AA10 | A | Final | 1A | Actionable PGx | 0.27 (3) | 0.09 (1) |
| ***CYP2D6*** | codeine | N02AJ07, R05DA04,  N02AJ06 | A | Final | 1A | Actionable PGx | 0.18 (2) | 0.18 (2 |
| ***CYP2B6*** | efavirenz | J05AG03, J05AR06,  J05AR11 | A | Final | 1A | Actionable PGx | 0.18 (2) | 0.09 (1) |
| ***CYP2C19*** | escitalopram | N06AB10 | A | Final | 1A | Actionable PGx | 0.18 (2) | 0.09 (1) |
| ***CYP2C19*** | imipramine | N06AA02 | B | Final | 1A |  | 0.18 (2) | 0.18 (2) |
| ***CYP2D6*** | imipramine | N06AA02 | B | Final | 1A | Actionable PGx | 0.18 (2) | 0.09 (1) |
| ***CYP2D6*** | risperidone | N05AX08 | B | Provisional | 1A | Informative PGx | 0.18 (2) | 0 (0) |
| ***CYP2D6*** | venlafaxine | N06AX16 | A/B | Provisional | 1A | Actionable PGx | 0.18 (2) | 0.09 (1) |
| ***CYP2C9*** | celecoxib | L01XX33, M01AH01,  C08CA51 | A | Final | 1A | Actionable PGx | 0.09 (1) | 0 (0) |
| ***CYP2D6*** | fluvoxamine | N06AB08 | B | Final | 1A | Actionable PGx | 0.09 (1) | 0 (0) |
| ***CYP2C9*** | piroxicam | M01AC01, R01AD53 | A | Final | 1A | Actionable PGx | 0.09 (1) | 0 (0) |
| ***CYP2C9*** | tenoxicam | M01AC02 | A | Final | 1A |  | 0.09 (1) | 0 (0) |

^€^ Drugs identified in the cohort among 45 PharmGKB evidence level 1A drug-gene pairs (Supplementary Figure 1).

**^¥^**Individuals whose predicted phenotype was designated as high-risk following the EHR Priority Result Notation and has also taken any specific drug associated with the high-risk phenotype.
